# Comparative proteomic investigation of metastatic and non-metastatic osteosarcoma cells of human and canine origin

**DOI:** 10.1371/journal.pone.0183930

**Published:** 2017-09-14

**Authors:** Jahnabi Roy, Kathryn L. Wycislo, Holly Pondenis, Timothy M. Fan, Aditi Das

**Affiliations:** 1 Department of Chemistry, University of Illinois Urbana–Champaign, Urbana, Illinois, United States of America; 2 Department of Pathobiology, University of Illinois Urbana–Champaign, Urbana, Illinois, United States of America; 3 Department of Veterinary Clinical Medicine, University of Illinois Urbana–Champaign, Urbana, Illinois, United States of America; 4 Department of Comparative Biosciences, Department of Biochemistry, Beckman Institute for Advanced Science, Division of Nutritional Sciences, Neuroscience Program and Department of Bioengineering, University of Illinois Urbana–Champaign, Urbana, Illinois, United States of America; University of South Alabama Mitchell Cancer Institute, UNITED STATES

## Abstract

Osteosarcoma is the most common bone cancer in dogs and people. In order to improve clinical outcomes, it is necessary to identify proteins that are differentially expressed by metastatic cells. Membrane bound proteins are responsible for multiple pro-metastatic functions. Therefore characterizing the differential expression of membranous proteins between metastatic and non-metastatic clonal variants will allow the discovery of druggable targets and consequently improve treatment methodology. The objective of this investigation was to systemically identify the membrane-associated proteomics of metastatic and non-metastatic variants of human and canine origin. Two clonal variants of divergent *in vivo* metastatic potential from human and canine origins were used. The plasma membranes were isolated and peptide fingerprinting was used to identify differentially expressed proteins. Selected proteins were further validated using western blotting, flow cytometry, confocal microscopy and immunohistochemistry. Over 500 proteins were identified for each cell line with nearly 40% of the proteins differentially regulated. Conserved between both species, metastatic variants demonstrated significant differences in expression of membrane proteins that are responsible for pro-metastatic functions. Additionally, CD147, CD44 and vimentin were validated using various biochemical techniques. Taken together, through a comparative proteomic approach we have identified several differentially expressed cell membrane proteins that will help in the development of future therapeutics.

## Introduction

Osteosarcoma (OS) is the most common form of primary bone cancer among children and young adults. The treatment options for OS include a combination of multi-agent induction chemotherapy, and radical excision of the tumor followed by adjuvant chemotherapy. Despite the aggressive treatment course, the survival rates are poor. For instance, in patients with localized disease, 5-year survival rates are approximately 65%; however, in the case of metastatic disease at diagnosis or recurrence, the 5-year survival rates are only 20% [1,2]. Although progress has been made towards improving treatment options, the early detection and subsequent control of metastasis have been challenging in OS.

The current approach towards the discovery of drug targets has focused on using high throughput peptide fingerprinting techniques to identify plasma membrane (PM) biomarkers in various cancers. Cell membranes are a dynamic and selective gatekeeper that controls the influx and efflux of multiple signaling molecules, and is responsible for multiple functions including adhesion, proliferation, migration and intercellular communication. Given their key roles in diverse, yet critical cellular functions, perturbations in plasma membrane proteins are associated with pathological states including cancer. Hence, the characterization of the membrane proteins in the cell surface of tumor cells can aid not only in early diagnosis, but also lead to the development of novel therapeutics. Recent evidence demonstrates the fluidity of cancer cell proteomic profiles with distinct classes of proteins being differentially expressed by tumor cells during metastatic progression [3]. Therefore, the current approach in this work is to identify and characterize the differential PM biomarkers of metastatic OS. This will be facilitated through the use of high throughput peptide fingerprinting that has been employed to identify targetable receptors in various cancers [4,5] as well as to identify differentially regulated markers in OS [6,7]. The thorough annotation of PM proteins differentially expressed by metastatic and non-metastatic OS cells holds promise to identify surrogate biomarkers of aggressive OS leading to earlier disease detection, as well as illuminate the biochemistry of metastasis.

A major limiting factor in the development of novel therapeutics in OS is the lack of suitable comparative animal models. While mouse models are commonly used for studying OS, they lack the degree of genetic heterogeneity as humans, making the study of OS oversimplified. Dogs are companion animals, which share the same environment with their human counterparts and have a greater genetic diversity than mice bred for research. Importantly, pet dogs also spontaneously develop OS that is genetically indistinguishable from human OS [8]. Hence dogs are more reliable comparative animal models that can aid in the discovery of novel OS therapeutics that can benefit both human and dogs. Currently there are no studies that show the correlation in cell surface receptors between human and canine OS. The identification of receptors that are upregulated in both humans as well as dogs, will allow for the development of novel therapeutics for both species in a parallel manner.

This will provide an opportunity to employ high throughput peptide fingerprinting to profile the global membrane proteome of human and canine OS and perform a cross-species analysis. For our study, we chose isogenic human and canine metastatic and non-metastatic cells. The availability of these cell lines allowed us to examine the differences in proteomic profiles that affect a metastatic phenotype without a significant genetic difference. Additionally, the comparison of metastatic versus non-metastatic cells, as opposed to tumorigenic and non-tumorigenic species, provides additional insight into factors that not only promote initial tumor formation but also those that promote metastasis.

Herein, we isolated the plasma membrane proteins and characterized the proteins by high-throughput peptide fingerprinting. We identified membrane proteins that were differentially regulated in both canine and human OS cell lines. We validated several targetable receptors using biochemical methodologies and immunohistochemistry studies in paired canine primary and metastatic tissues. Finally, overall changes in GPCR activity were measured that corroborated with the changes in the GPCR distribution in metastatic and non-metastatic samples as identified from the peptide fingerprinting data. Taken together, in this work we identify cross-species trends in expression of cell surface receptors between metastatic and non-metastatic OS that were consistent between both human and canine species. Several of these receptors are known targets in various cancer types and therefore drugs targeting of these receptors can be repurposed to develop OS therapeutics.

## Materials and methods

### Materials

Antibodies- CD44 (Santa Cruz Biotechnology sc-18849), Vimentin (Abcam ab16700) and CD147 (Abcam ab108317). Secondary antibodies for confocal microscopy and flow cytometry- Goat anti-Rabbit IgG (H+L) Secondary Antibody, Alexa Fluor^®^ 488 conjugate (ThermoFischer Scientific A-11034) and Goat anti rat (Santa Cruz Biotechnology sc-2011). Image IT Fx Signal Enhancer (R37107), ProLong Gold Antifade Mount (P10144), 4’, 6-diamidino-2-phenylindoleg (DAPI) were obtained from Thermo Fischer Scientific. IHC- all IHC reagents, except primary antibodies and 61–9520 are from Biocare Medical (biocare.net). 61–9520 is from Fisher Scientific (thermofisher.com)

### Cell culture

Human cell lines HOS and 143B and canine cells POS and HMPOS were cultured in DMEM (Gibco, Invitrogen, Carlsbad, CA, USA) supplemented with 10% fetal bovine serum (FBS) and 1mgml^–1^ penicillin–streptomycin (Gibco, Invitrogen) at 37°C and 5% CO_2_ in a humidified incubator. All cell lines were verified via STR analysis. The human cell lines HOS and 143B were verified at The University of Arizona Genetics Core and the canine cell lines POS and HMPOS were verified at Colorado State University Flint Animal Cancer Center.

### Extraction of plasma membranes

Membrane extraction was done as previously published (7, 58). All steps were carried out at 4°C. Briefly, about 60 million adherent cells were washed with phosphate-buffered saline (PBS), scraped using a plastic cell lifter, and lysed in 1 mL solution containing 0.2 mM EDTA and 1 mM NaHCO_3_ and 20 μL protease inhibitor (Nacalai USA, Inc.), using a glass homogenizer. The nuclear and unbroken cells were removed through centrifugation a 200×g, and the supernatant was collected and centrifuged for 30 min at 25,000 rpm. The cell pellets were resuspended in 1 mM NaHCO_3_ in an approximate ratio of 1 mL per 5×10^8^ cells and used for PM separation by two-phase systems. Suspended cell pellets were added to the top of 14 g of the dextran–poly (ethylene glycol) mixture (6.6% Dextran T500, 6.6% PEG 3350, 0.1 M sucrose, 5 mM K_3_PO_4_, pH = 7.2). After mixing up and down for 40 times, the tube was centrifuged for 5 min at 750×g. The PM-enriched up phase was collected and purified again as described above. The up phase was diluted with 1 mM sodium bicarbonate and centrifuged at 100,000×g for 2 h in a SW32 rotor.

### Peptide fingerprinting

#### Protein identifications using CID mass spectrometry

Sample preparation: Sample cleanup was done using G-Biosciences (St. Louis, MO) Perfect Focus according to manufacturer’s instruction. 1–100μl of protein solution (containing 10μg protein per sample) was transferred into a 1.5ml microfuge tube. 300μl of reagent UPPA-I was added and mixed well. This was incubated at 4°C for 15 minutes. 300μl of reagent UPPA-II was added to the protein mixture and the tube was vortexed. The tube was then centrifuged at 15,000x g for 5 minutes to form a protein pellet. Carefully, without disturbing the pellet, a pipette tip was used to remove & discard the entire supernatant. The tube was centrifuged again for 30 seconds. A pipette tip was used to remove the remaining supernatant. 40μl of FOCUS-Wash was added on top of the pellet. The tube was centrifuged again for 5 minutes and the wash was discarded. 25μl of milliQ water was added on top of the pellet and the tube was vortexed. 1ml OrgoSol Buffer, pre-chilled at –20°C, was added to the tube containing protein suspension. The tube was vortexed to suspend the pellet. The tube was incubated at –20°C for 30 minutes. Periodically the tube was vortexed for 20–30 seconds. The tube was centrifuged at 15,000xg for 5 minutes to form a pellet and the supernatant was removed and discarded. The pellet was air dried and the white pellet turned translucent on air drying.

Digestion with trypsin: Sample was digested with MSG-Trypsin (G-Biosciences, St. Louis, MO) at a w/w ratio of 1:10 using a CEM Discover Microwave Digestor (Mathews, SC) at 55°C and maximum power of 60 watts for 30 minutes. Digested peptides were lyophilized in a Virtis -55 lyophilizer to dryness. The digested peptides were dissolved in 5% acetonitrile + 0.1% formic acid for LC/MS.

LC/MS: LC/MS was performed using a Thermo Dionex Ultimate RSLC3000 operating in nano mode at 300 microliters/min with a gradient from 0.1% formic acid to 100% acetonitrile + 0.1% formic acid in 120 minutes. The trap column used was a Thermo Acclaim PepMap 100 (100 μm x 2 cm) and the analytical column was a Thermo Acclaim PepMap RSLC (75 μm x 15 cm). 2 micrograms of the digested peptides were loaded per injection.

Mass spectrometry was performed on a Thermo LTQ Velos ETD Pro Mass spectrometer using data dependent MS/MS analysis on the top five most intense ions detected in the precursor scan mode. Previously detected ions were automatically excluded for 60 seconds to allow deeper coverage of the less abundant ions.

### Data analysis

The Xcalibur raw file was converted by Mascot Distiller into peaklists that were submitted to an in-house Mascot Server and searched against specific NCBI-NR protein databases. The list of proteins obtained was filtered by a p < 0.05 for a 95% confidence value. Additional data about peptide fingerprinting parameters can be found in SI. The peak lists have been uploaded into external repository as indicated at the end of this section. This list of proteins was then classified by their subcellular location (to filter out non- membrane contaminants) and plasma membrane candidates were classified into various functional classes. The emPAI values were normalized to the total emPAI value and compared as a percentage to the overall composition.

### Quantitation: emPAI protocol

The **E**xponentially **M**odified **P**rotein **A**bundance **I**ndex (emPAI) offers approximate, label-free, relative quantitation of the proteins in a mixture based on protein coverage by the peptide matches in a database search result. Developed by Ishihama and colleagues,Exponentially modified protein abundance index (emPAI) is a quantification unit for estimation of absolute protein amount in proteomics as calculated from the number of sequenced peptides per protein [9].

Unlike the other quantitation protocols, the information required for emPAI is always present in a search result, and there are no parameter settings, as long as the MS/MS search contains at least 100 spectra.

$$emPAI=10^{\frac{Nobserved}{Nobservable}}-1$$

Where *N*_*observed*_ is the number of experimentally observed peptides and *N*_*observable*_ is the calculated number of observable peptides for each protein. The tricky bit is deciding what to include and what to exclude in these two counts.

### Western blot

Based on protein concentration measurements, equal amounts of plasma membrane preparations generated from all cells were resolved by 4–20% SDS-PAGE (Pierce) and transferred onto a PVDF membrane. The membranes were blocked in PBS, 0.1% Tween 20, 5% nonfat dry milk powder for 1 h at room temperature and incubated with primary antibody for 16 h at 4°C followed by washing and incubation with HRP-conjugated secondary antibody for 1 h at room temperature. All antibody incubations and washing steps were carried out in PBS, 0.1% Tween 20. The immunoreactive bands were visualized using an ECL Western blot kit (Amersham Biosciences). The western blots were normalized by Ponceau staining [10].

### Confocal microscopy

Cells were grown in 8 well chamber slides (Ibidi, Verona, WI), overnight. The cells were washed with warm phenol red free DMEM free of FBS. The cells were fixed with warm phenol red free DMEM free of FBS containing 4% methanol free paraformaldehyde at room temperature for 10 minutes. The wells were washed 3X with PBS, and permeabilized with 0.1% Triton-X 100 in PBS for 5 minutes. The cells were then blocked with 5- drops of IT signal FX solution (SFX kit, Invitrogen) for 30 min. at room temperature. After 3 washes with PBS, the slides were blocked with 3% BSA in PBS for 30 min at room temperature. The primary antibody, which was diluted in PBS containing 3% BSA (1:100), was added to the cells, and samples were incubated overnight at 4°C. Cells were rinsed 3X with PBS, secondary antibody was added in PBS containing 3% BSA (1:200), and cultures were incubated for 1 hour at room temperature. After 3 washes with PBS, nuclei were counterstained with DAPI for 15 minutes at room temperature, samples were washed 3Xwith PBS, and cells were mounted with 5 drops of ProLong Gold solution and allowed to dry in a chemical fume hood for 24 hours before imaging. Imaging was performed through the bottom of the chamber slides in a Nikon A1R confocal laser microscope system. The data was analyzed using ImageJ and NIS Elements Confocal Microscope Imaging Software. Laser power and other parameters indicated in Supporting Information.

### GPCR activity assay

Intracellular cAMP levels were measured as an indicator of GPCR activity by a competitive immunoassay based on time-resolved fluorescence resonance energy transfer (TR-FRET) between the fluorescent reporter cAMP-d2 and anti-cAMP-cryptate using a cAMP dynamic 2 kit (Cisbio Bioassays). Cells were cultured in 96-well cell culture plates with a concentration at 26000 cells/well overnight at 37°C with forskolin or with DMSO control. The medium was removed and lysis buffer (50 μl/well) supplied in the kit was added to lyse cells in the presence of 3-isobutyl-1-methylxanthine (IBMX- phosphodiesterase inhibitor). Then, 10 μl cell lysate/well was transferred to 384-well microplates followed by the addition of cAMP-d2 and anti cAMP- cryptate (5 μl each). Each cell line was measured in triplicate to account for variability as well as two separate experiments weeks apart to ensure no batch-to-batch variability. Incubation for 1 h at room temperature was followed by a read out with Analyst HT (Molecular Devices, LLC). Data processing and quantification of cAMP were performed according to the manufacturer’s instructions.

### Flow cytometry

Cells were washed with PBS, scraped and pelleted at 2000 RPM for 5 min and divided into flow cytometry tubes. 0.1 mL of Fixation/ Permeabilization solution was added to the cells (BD Bioscience Fixation/Permeabilization Solution Kit catalog no. 554714) and pulse vortexed. The cells were incubated for 1h at room temperature. Cells were washed with 0.1 mL of Permeabilization/Wash Buffer followed by centrifugation and decanting of supernatant. Cells were suspended in 0.2 mL of Permeabilization/Wash Buffer and the primary antibody was incubated (1:100) overnight at 4°C. The cells were pelleted and washed 3X with 0.2 mL of Permeabilization/Wash Buffer and secondary antibody was incubated (1:200) at room temperature in the dark for 1h. The cells were pelleted and washed 3X with 0.2 mL of Permeabilization/Wash Buffer and resuspended in Permeabilization/Wash Buffer for flow cytometry in Accuri C6 instrument.

### Immunohistochemistry in cell pellets

Confluent cell cultures were collected, washed in PBS, and pelleted by centrifugation. Supernatant PBS was discarded and the washed cells were suspended in 1 mL of 10% formalin for 1 hour, followed by centrifugation and formalin removal. 1 mL 4% melted agarose gel was then added to the fixed, pelleted cells, which were immediately vortexed to create a uniform cell suspension and then briefly centrifuged to create an agarose-embedded cell pellet.

### Canine tumor samples: Immunohistochemistry in paired metastatic and primary canine tumor samples

All tissue controls for antibody optimization and specificity of staining were obtained from canine necropsy specimens at the Veterinary Diagnostic Lab at the College of Veterinary Medicine, University of Illinois, Urbana-Champaign. The skin samples (negative control) were taken from a necropsy specimen without skin disease. The kidney samples (positive control) were taken from a necropsy specimen without kidney disease. Canine appendicular OS tissue blocks and their corresponding lung metastases tissue blocks were also retrieved from the University of Illinois Veterinary Diagnostic Laboratory for immunohistochemistry (IHC). Formalin-fixed, agarose-embedded cell pellets from the previously utilized cell lines were also processed into paraffin-embedded tissue blocks for IHC. Slides cut from the tissue blocks were deparaffinized in xylene and rehydrated in ethanol. Antigen retrieval was performed using Diva Decloacker (DV2004). Endogenous peroxidase activity was blocked using Peroxidazed 1 (PX968) for 5 minutes, followed by blocking of non-specific background staining using Background Punisher (BP974) for 10 minutes. Blocked slides were incubated with either anti-human CD147 (ab108317) for 1 hour and 15 minutes or anti-mouse CD44 (sc-18849) for 1 hour at dilutions of 1:100 and 1:50, respectively. CD147 slides were then incubated with a rabbit-on-canine horseradish peroxidase (HRP) secondary antibody (RC542) for 30 minutes and CD44 slides were incubated with a rabbit-on-rat HRP secondary antibody (61–9520) for 30 minutes at 1:100. The chromogen 3–3’-diaminobenzidine (DAB) (IPK5010) was applied for 5 minutes to develop slides, followed by counterstaining with Cat hematoxylin (CATHE). All steps were followed by washing in either wash buffer or DI water, as appropriate. Negative controls for the samples were processed identically in the absence of primary antibody. Using ImageJ software, immunohistochemical-staining positivity was expressed as normalized pixel intensity per positive cell.

## Results

We chose two human and two canine isogenic cell lines for proteomic profiling. The human cell lines HOS (non-metastatic) and 143B (metastatic) have been established as clinically relevant orthotopic models for human OS [11]. Both HOS and 143B cells were derived from parent TE-85 cells through methylnitronitrosoguanidine (MNNG) or Ki-ras transformation respectively. The canine cells POS (non-metastatic) was derived from spontaneous canine osteosarcoma and HMPOS (metastatic) were established from five subcutaneous implantation cycles of lung tumor deposits [12]. We choose cell lines over tumor tissue in order to avoid heterogeneity in tissue samples obtained from tumor tissue [13]. The cell lines chosen here have been previously shown to be appropriate models for OS [11,12]. Additionally, since the cell lines are isogenic, it reduces the possibility of differential proteomic patterns arising due to different cellular origins. Finally, to avoid differences due to nutritional treatment, all cells were harvested at 80–90% confluency, and the samples utilized for different methods of detection as described below.

The overall workflow is outlined in Fig A in S1 File. Briefly, the cells were scraped instead of trypsinizing to maintain the integrity of cell surface proteins. The plasma membrane fraction was enriched by aqueous two phase extraction protocol in order to remove cytoplasmic contaminants that may overwhelm the signal from low-population PM proteins. Subsequently peptide fingerprinting was done that provided a list of proteins that was then classified by function to identify global proteomic changes (Table A- Table D in S1 File).

### Proteomic profiles from peptide fingerprinting

**Fig 1:**
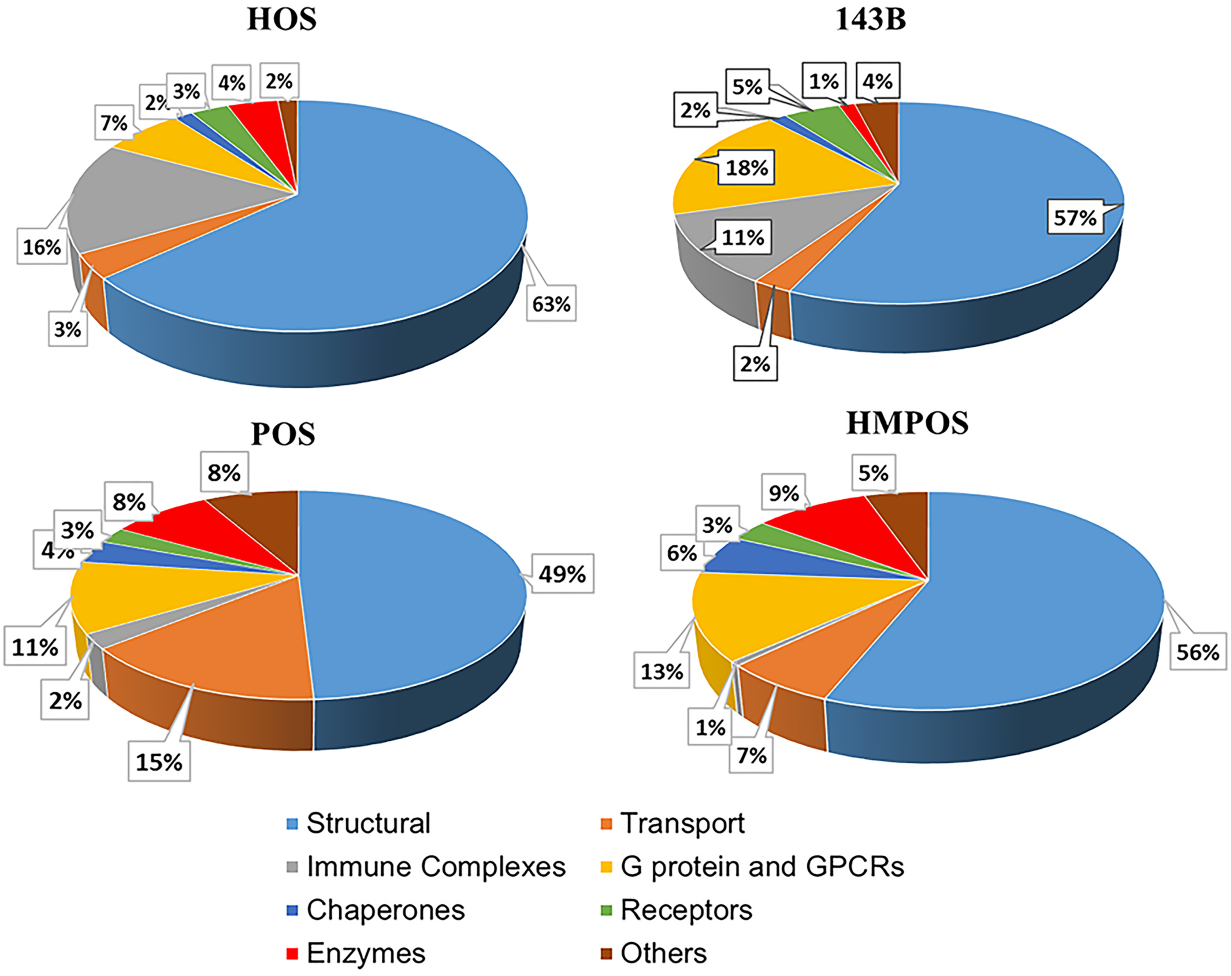
Fig shows global membrane proteomic composition across various cell lines classified according to function. The colors are as mentioned in the key. HOS and POS are the non- metastatic human and canine cell lines and 143B and HMPOS are the metastatic human and canine cell lines respectively.

Peptide fingerprinting is a high throughput method that provides a list of proteins and their relative abundances. For each sample set, 10 μg of protein from each set was taken to analyze as indicated in the materials and methods section to remove any differences that may arise due to different quantities of proteins used for each sample. This is the first control towards standardizing the protein abundance. Peptide fingerprinting provided a list of over ~500 proteins per cell line. It is a semi-quantitative technique and provides the relative amount of specific proteins in a given mixture through a measure of the exponentially modified protein abundance index (emPAI) [9] as explained in the materials and methods section. Other similar measures include spectral counts and peptide counts. The emPAI value can help understand the overall protein composition of the membrane. However, it is not a consistent measure across various samples. Thus, two different methods were used for standardizing the emPAI aside from loading the same amount of protein initially. Each proteins individual emPAI was normalized to the total emPAI of that set. Furthermore, trends of proteomic distribution can be compared in the human and canine sets across the two species. It is important to note that many proteins were undetectable as they present in low abundance and is below p value of 0.05, which was set as a significance cut-off for matching the peptide fingerprinting results to the MASCOT database (Table A in S1 File). The analysis of the peptide fingerprinting data, as seen in Fig 1, revealed various interesting trends that are discussed below. The corresponding calculations are represented in Table 1. The peptide fingerprinting protocol was used only to generate a list of potential candidates and therefore not replicated. Statistical significance from peptide fingerprinting was only utilized to filter out targets that have questionable peptide match and therefore may not be valid targets. The candidates of interest were taken forward for downstream validations where each biochemical validation was replicated for at least n = 3.

**Table 1:** Table shows the percentage of proteins in each category in each cell membrane. Table 1 represents the calculations reflected in Fig 1. HOS and POS are the non-metastatic human and canine cell lines and 143B and HMPOS are the metastatic human and canine cell lines respectively.

	HOS	143B	POS	HMPOS
**Structure**	63.48	56.68	49.11	56.01
**Transport**	3.41	2.46	14.82	6.59
**Immune Complexes**	15.69	11.09	2.26	0.62
**G protein and GPCRs**	6.74	18.3	10.99	13.02
**Chaperones**	1.6	1.47	3.61	5.89
**Receptors**	3.19	4.88	2.57	3.24
**Enzymes**	4.23	1.35	8.52	9.4
**Others**	1.66	3.77	8.11	5.24
**Total**	100	100	100	100

#### Structural proteins

The structural proteins form the largest class of proteins in the membrane proteome (Fig 1 and Table 1). They play various roles including maintenance of cell shape, adhesion, and migration. In our proteomic data, we observed several different structural proteins that are differentially regulated as discussed below.

#### Intermediate filaments

We observe a significant increase in the total content of intermediate filaments (Fig B in S1 File and Table 1), which comprises of mainly keratins and vimentin. Vimentin is a marker for the epithelial to mesenchymal transition in various epithelial cancers and has been discussed in further detail in the validation of targets section below.

#### Collagens

Collagens are important constituents of the extracellular matrix. The increase of collagen expression in the tumor microenvironment has been correlated with tumor progression [14]. We observed an overall increase in the amount of collagens in both metastatic cell lines as compared to the non-metastatic cell lines. In human cells, there was a 48% increase in the collagen content of metastatic cells versus non-metastatic cells. Likewise, in the canine cell lines, the metastatic cells expressed 46% more collagen than the non-metastatic cells (Fig B in S1 File).

#### Annexins

Annexins A1, A2 and A5 have been found on the cell surface and are involved in membrane scaffolding and are responsible for anchoring several membrane proteins. In our studies, in both cell lines the metastatic cells produce only about half the annexin A1 as the non-metastatic (Table 2). This agrees with previous literature where reduced Annexin A1 expression was significantly associated with advanced breast cancer [15].

**Table 2:** Table shows the relative emPAI of selected differentially regulated proteins in the human and canine osteosarcoma cells. HOS and POS are the non-metastatic human and canine cell lines and 143B and HMPOS are the metastatic human and canine cell lines respectively. emPAI of each protein is normalized to total emPAI. The ratios 143B/HOS and HMPOS/POS show fold change in expression.

Protein	HOS	143B	143B/ HOS Fold Change	POS	HMPOS	HMPOS/ POS Fold Change
**Keratin Type 1**	2.31	4.31	1.87	2.99	3.80	1.27
**Keratin Type 2**	0.30	2.48	8.30	2.48	3.41	1.38
**Vimentin**	6.06	7.20	1.19	4.34	13.69	3.16
**Annexin A1**	0.86	0.45	0.52	1.91	1.01	0.53
**Annexin A2**	36.85	17.11	0.46	5.74	8.63	1.50
**MHC Class I**	2.73	0.28	0.10	0.15	0.00	0.00
**KRAS**	0.40	1.53	3.80	0.05	0.24	4.48
**CD44**	0.17	0.33	1.30	0.00	0.16	
**CD 147 (Basigin)**	0.42	0.67	1.6	0.22	0.46	2.12
**Monocarboxylate transporter I**	0.07	0.11	1.46	0.00	0.09	
**CD 98**	0.15	0.18	1.20	0.00	0.07	
**Integrin β1**	0.21	0.30	1.47	0.00	0.00	0.00

Annexin A2 is a calcium-dependent, phospholipid-binding protein. It plays multiple roles in regulating cellular functions, including angiogenesis, proliferation, apoptosis, cell migration, invasion and adhesion. It has been implicated in several cancers [16–19]. The upregulation of annexin A2 in various cancers is 5.74, the metastatic HMPOS show values of 8.63 (Table 2). However, with the human cell lines, a reverse trend is observed. The non-metastatic HOS line has an emPAI value of 36.85, whereas the metastatic 143B cells show 17.11 emPAI (Table 2).

#### Immune markers

Immunological eradication of cancer is an important function of the immune system. This is evident from literature that indicates that cancer cells downregulate their immune markers to evade this response [20]. The major histocompatibilty complex (MHC) is a key component of the immune system. Our findings through peptide-fingerprinting mass spectrometry show a significant change in the expression levels of MHCs and immunoglobulins within the metastatic and non-metastatic human cells. The overall expression of immune-related proteins reduces approximately 80% in human metastatic cell line and about 50% in the canine metastatic cell line (Fig CB in S1 File). A much lower density of MHC class I is also observed in the metastatic versus the non-metastatic cells. In the human lines, 143B expresses only 0.10 times MHC Class I than HOS, and in the canine lines the expression of MHC Class I in HMPOS was not detectable for p<0.05 whereas POS expression was detected. The down regulation of MHC class of molecules has been intrinsically linked to cancer as it assists in tumor immune escape [21]. Specifically in OS, the down regulation of MHC class I has been shown in patient samples and it is suggested that it restricts cytotoxic T lymphocyte pathway, which plays a major role in immune surveillance of patients with OS [22].

### G proteins and GPCRs

G-protein coupled receptors (GPCRs) are a large family of cell-surface receptors that are responsible for signal transduction in a large number of cellular processes. Malignant cancer cells are known to hijack the normal physiological functions of GPCRs to survive, proliferate autonomously, evade the immune system, increase blood supply, invade their surrounding tissues and disseminate to other organs. Increased GPCR expression has been observed in various cancers including breast cancer [23]. Herein we observe an overall increase in percentage of GPCRs from 7% of total proteins identified in HOS cells to 18% of total in the human metastatic143B cells (Fig CA in S1 File). A similar trend, albeit of smaller magnitude, is observed for the canine lines, where 11% of total proteins identified are GPCRs in POS cells and 13% of total identified in HMPOS (expression difference in the human lines and canine lines is depicted in Fig CA in S1 File).

A class of small G-proteins, the RAS family, has also been implicated in several oncogenic processes [24] and is the most common gene mutation observed in various cancers [25]. This is supported by our proteomics results where a significant overexpression of KRAS is observed in the metastatic cells versus the non-metastatic cells in both human and canine lines (Table 2). KRAS expression was also validated for the four cell lines by western blotting (Fig E in S1 File), which shows significant overexpression in the 143B cells versus the HOS cells. However, the differences between the canine cell lines in KRAS by western blotting were not significant.

Finally, a specific class of GPCRs that has been aberrantly been linked to cancer are olfactory receptors (ORs). Initially thought to be an anomaly, genes responsible for ORs showing significant mutation have been observed in various cancers [26–28]. Our findings also suggest an increase in the overall expression of ORs in the metastatic cell line as compared to the non-metastatic cell lines. In our peptide fingerprinting results OR7D2 and OR4N4 were observed in the 143B cell membrane proteome and OR4C11 and OR4C16 were identified in HMPOS membrane proteome. There were no ORs identified in the corresponding non-metastatic cells HOS and POS at p<0.05, which was set for our peptide fingerprinting data.

### GPCR activity assay

**Fig 2:**
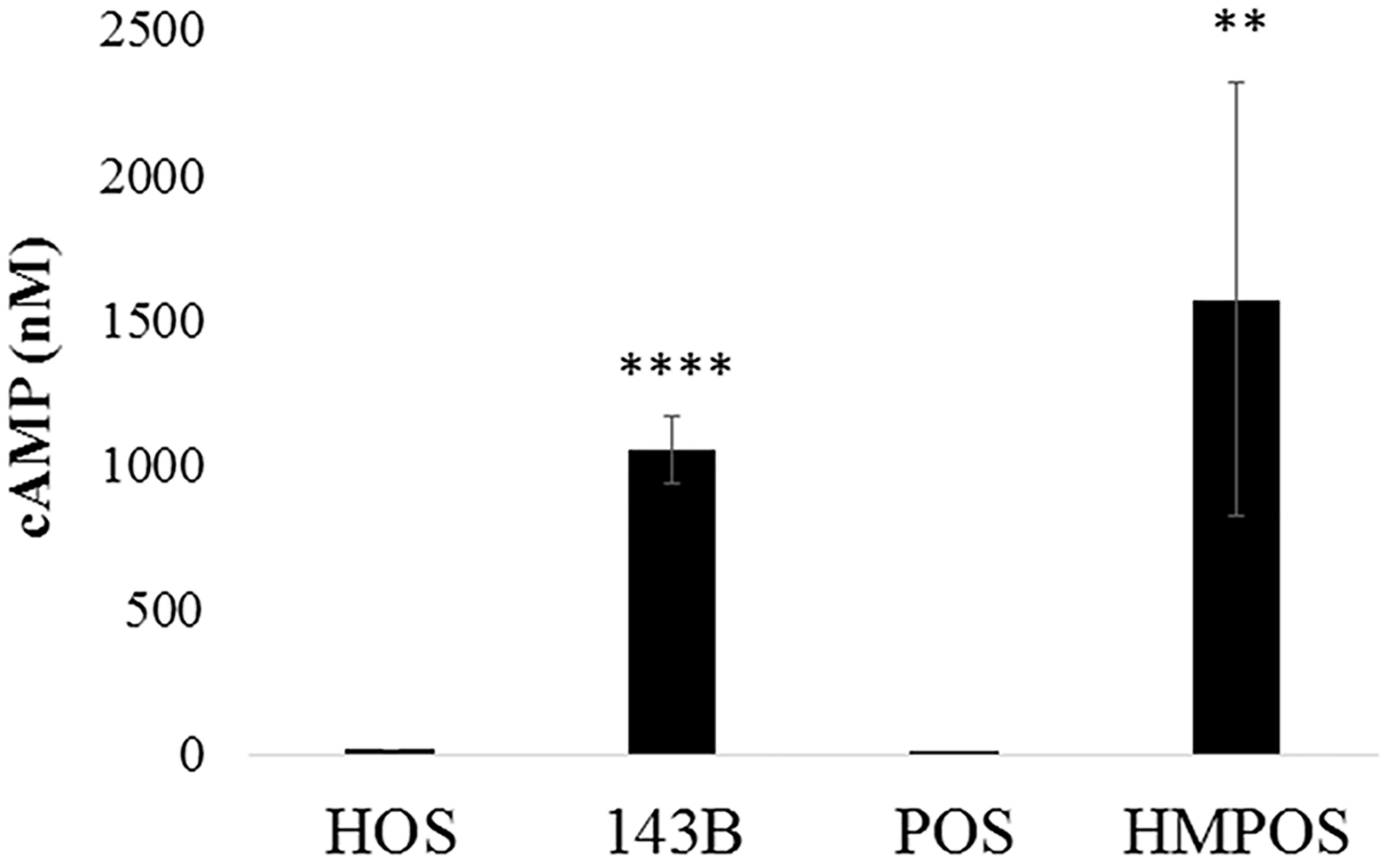
Fig shows the relative GPCR activity as measured by cAMP concentration. The experiment was performed in biological triplicates (and technical duplicates) with n = 2 (total separate trials). **** p <0.0001 and ** p<0.01.

Peptide fingerprinting indicated that GPCR levels are overall upregulated in metastatic vs. non-metastatic cells. To investigate whether the expression levels correspond with a concomitant increase in activity, a GPCR activity assay was performed. Cyclic AMP (cAMP) is a secondary messenger of several GPCR pathways- the G_i_ and G_s_, and thus cAMP levels were measured as an indicator of GPCR activity. Briefly, cells were stimulated with forskolin (stimulates G_s_ class of G-proteins) or DMSO and then lysed, and cAMP was measured by a TR-FRET-based method. As seen in Fig 2, the overall levels of cAMP were significantly higher in 143B and HMPOS cells as compared to their non-metastatic counterparts, HOS and POS, respectively, indicating higher metastatic activity is accompanied by increase in GPCR activity.

### Receptors and transporters

#### Integrin β1

Integrins are a family of transmembrane glycoprotein receptors that mediate cell-matrix and intercellular interactions [29]. Integrins play a key role in the invasion process by inducing various matrix metalloproteinases (MMPs) and lead to the breakdown of extracellular matrix (ECM). We observed an increase in integrin β1 expression in the metastatic human cell line by nearly 1.5 times as compared to the non-metastatic line (Table 2). No integrin β1 was observed in the canine lines at p< 0.05 in the peptide fingerprinting data. CD98 is known to associate with integrin signaling and thereby increase cell growth and proliferation [30,31].

#### CD147

Tumors interact with their microenvironment through enzymes known as matrix metalloproteinases (MMP). CD147, also known as Basigin, is an MMP inducer that is overexpressed and promotes tumor progression, invasion, and metastasis by stimulating MMP secretion [32,33]. Due to the pivotal role of CD147 in cancer, this molecule has been termed a cancer-associated biomarker [34] and serves as a target for cancer therapy [35]. In our studies we also observed an increase in CD147 expression in the metastatic lines for both species. In the human cells, there was a 1.6-fold increase in CD147 expression, whereas in the canine cells there was a greater than twofold increase in expression which was further validated by western blotting and confocal microscopy. Interestingly, CD147 is known to be associated with increased expression of monocarboxylate transporter (MCT) expression in various cancers including cervical carcinoma [33,36]. Our results corroborate this, with the increased expression of MCT1 by over twofold for the human lines. In the canine lines, POS expression of MCT1 was not detectable for p<0.05; however, the metastatic HMPOS line shows expression. These insights can lead towards targeting not only specific proteins but also cellular pathways that are involved in increasing the metastatic potential of the cells. Due to potential implication of CD147 in therapy, this target was further validated by biochemical methods as indicated in the validation section.

#### CD44

CD44 is a multifunctional cell surface glycoprotein involved in cell proliferation, cell differentiation and cell migration. The crucial involvement of this protein in cell processes makes it highly important in the pathologic state of cancer cells. In our studies, we observed CD44 to be upregulated in metastatic cell lines. In human cells, it was 1.3 times in 143B cells versus HOS cells. In canine cells, while no CD44 was detected for POS at p<0.05, a value of 0.16 was observed for HMPOS cells. Previously in OS, a higher level of CD44 has been shown to correlate with poor prognosis (42).

#### CD98

CD98 or 4F2 antigen is a large neutral amino acid transport protein that has been implicated in various cancers [37,38]. In our results, we observed a 1.2-fold increase in the metastatic human cell line for CD98 expression. In the canine lines, CD98 was not detected in the non-metastatic line but it was detected in the metastatic line indicating an increase. In OS, increased expression of CD98 has been observed in patient samples [39].

### Validation of selected targets using western blots and confocal microscopy

Several differentially regulated targets were further validated as seen in Table 2. Three targets that were validated by western blotting and confocal microscopy are: CD44, CD147 and vimentin. These targets were chosen due to their implications in various other cancers. For western blotting samples, since the samples are membrane fractions and not whole cells, housekeeping proteins like GADPH and beta-actin do not present themselves as ideal controls. Thus Ponceau staining was used as a loading control as has been indicated in materials and methods and has been shown in Fig D in S1 File. In general 5 μg of total protein was loaded for CD44 and CD147 in each trial and 1 μg was loaded for vimentin in each trial. A total of n = 3 was performed for all western blotting samples and n = 2 was performed for confocal microscopy. The quantification for confocal microscopy images is shown in Fig F in S1 File.

#### CD44

**Fig 3:**
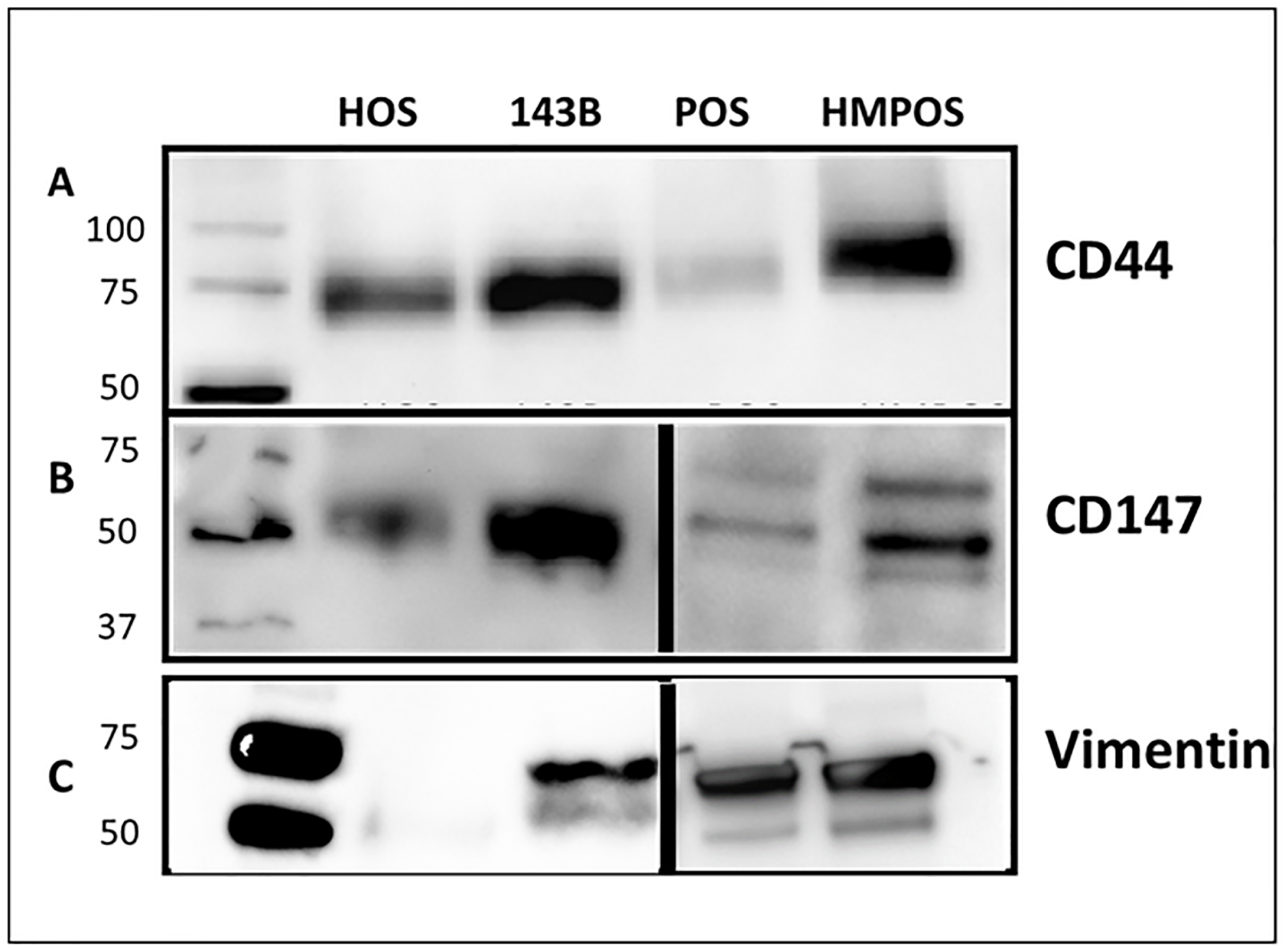
Western blot showing the expression of (A) CD44, (B) CD147 and (C) Vimentin. Lanes from left to right: ladder, HOS, 143B, POS and HMPOS. HOS and POS are the non-metastatic human and canine cell lines and 143B and HMPOS are the metastatic human and canine cell lines respectively.

**Fig 4:**
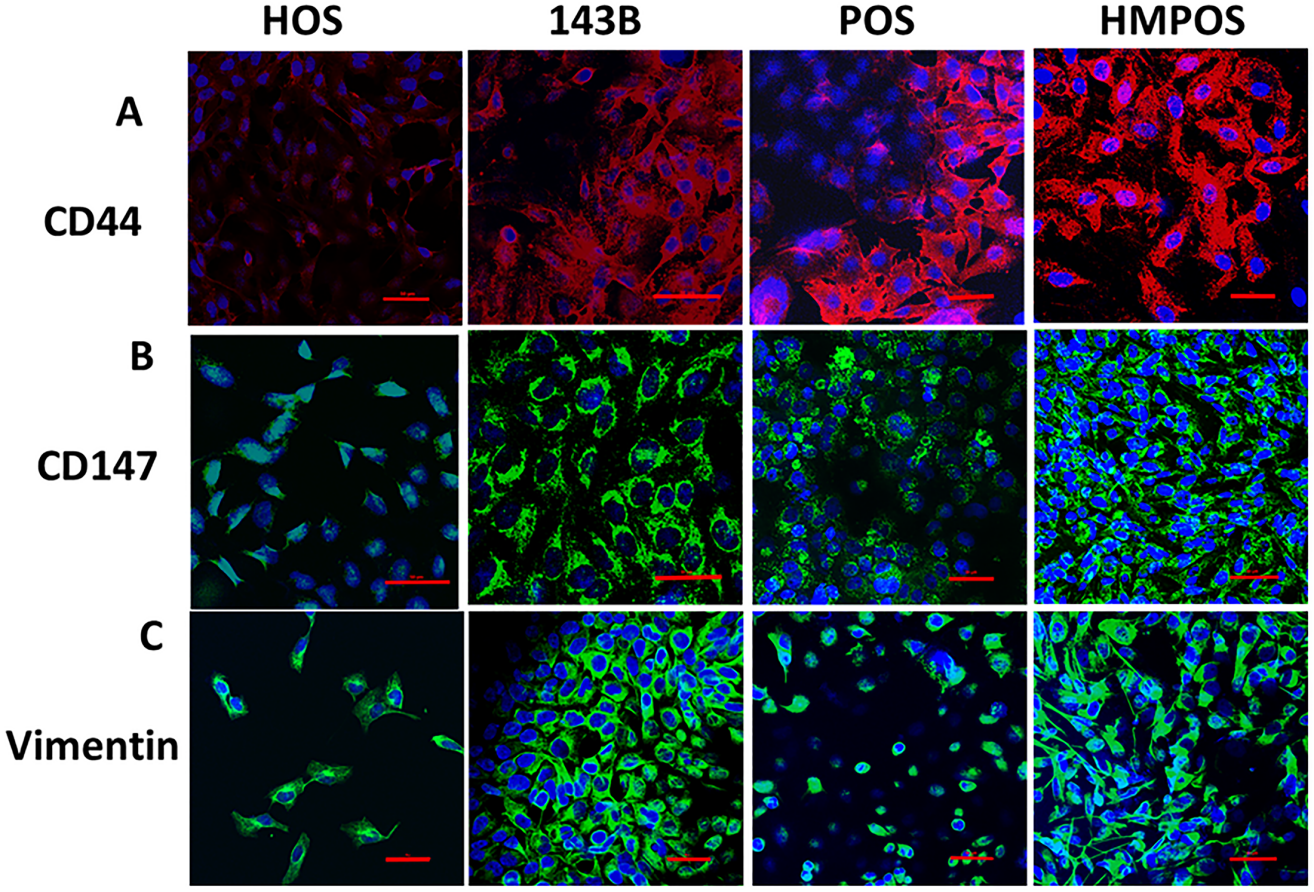
Confocal microscopy images showing the expression of (A) CD44, (B) CD147 and (C) Vimentin. HOS and POS are the non- metastatic human and canine cell lines and 143B and HMPOS are the metastatic human and canine cell lines respectively. Blue staining of nuclei with DAPI and red staining of CD44 with phycoerythrin secondary and green staining of Alexa Fluor 488 for CD147 and vimentin. Red bar in each section corresponds to 50 μm. Details of settings and quantification are indicated in S1 File.

In our studies, we observed CD44 to be upregulated in metastatic cell lines. In human cells, it was 1.89 times in 143B cells versus HOS cells. In canine cells, while no CD44 was detected for POS at p<0.05, a value of 0.12 was observed for HMPOS cells. We validated CD44 in both human and canine cells by western blotting and confocal microscopy. As observed in Figs 3A and 4A, a strong upregulation of CD44 is observed when evaluated using the two detection methods.

#### CD147

In our peptide fingerprinting data we observed an increase in CD147 expression in the metastatic lines for both species. In the human cells, there was a 1.6-fold increase in CD147 expression, whereas in the canine cells there was a greater than twofold increase in expression which was further validated by western blotting and confocal microscopy. As seen in Figs 3B and 4B, an increased expression of CD147 is observed in the metastatic versus non-metastatic cell lines.

#### Vimentin

Vimentin is an intermediate filament expressed in mesenchymal cells and is responsible for maintenance of cell shape and integrity, migration, and adhesion. In our results, we observed a 1.2-fold increase in vimentin expression in the human cell lines and an over threefold increase in vimentin expression in canine metastatic cell lines. This target was validated by western blotting and confocal microscopy and as shown in Figs 3C and 4C, these results corroborate our findings from peptide fingerprinting analysis.

### Validation of selected targets using immunohistochemistry in paired primary and metastatic canine tumor samples

**Fig 5:**
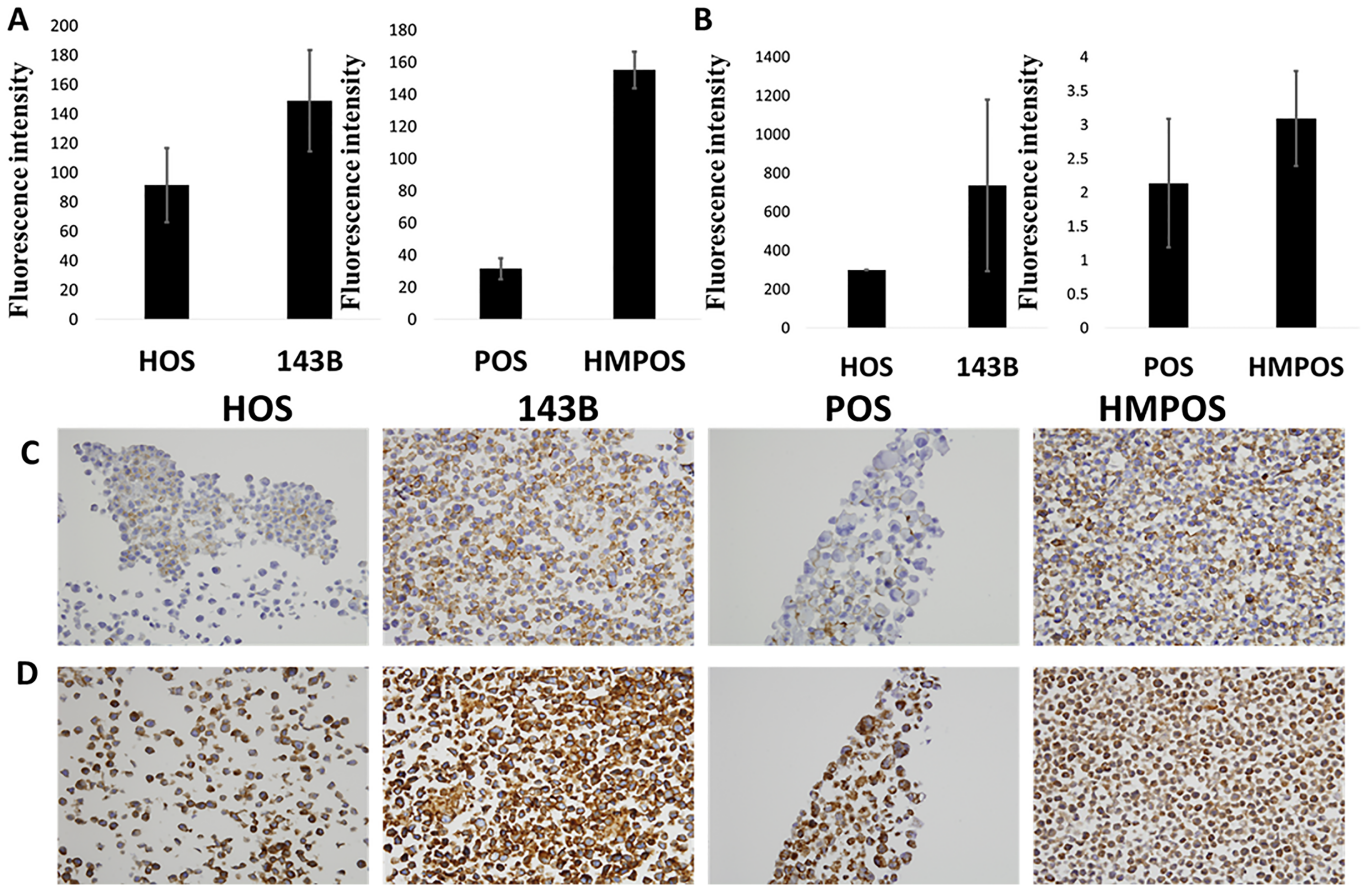
Flow cytometry fluorescence levels showing the expression of (A) CD44 and (B) CD147. Cell pellet immunohistochemistry showing the expression of (C) CD44 and (D) CD147. HOS and POS are the non- metastatic human and canine cell lines and 143B and HMPOS are the metastatic human and canine cell lines respectively. Details of quantification are indicated in SI.

Using western blots and confocal microscopy, we validated the upregulation of three targets that can also observed to be upregulated-using peptide fingerprinting. Two of these targets, CD44 and CD147, were further investigated, as they are potential druggable targets. First, flow cytometry was performed. For each sample, flow cytometry was performed in triplicates for n = 3. As observed from Fig 5A and 5B, in both human and canine cell lines, the metastatic cells showed higher levels of CD44 and CD147 as compared to the non-metastatic counterparts. Furthermore, immunohistochemical (IHC) analysis was performed for both CD44 and CD147 on formalin fixed cells for n = 3. As observed in Fig 5C and 5D, the staining of CD44 and CD147 is greater on the metastatic cell lines 143B and HMPOS as compared to their non-metastatic counterparts. This is in agreement with our western blotting, confocal microscopy and flow cytometry data. The quantification is shown in Fig G in S1 File. This also indicates that no changes in staining trends are observed due to formalin fixation, which was done for cell pellets in immunohistochemistry (IHC) but not for other biochemical techniques.

**Fig 6:**
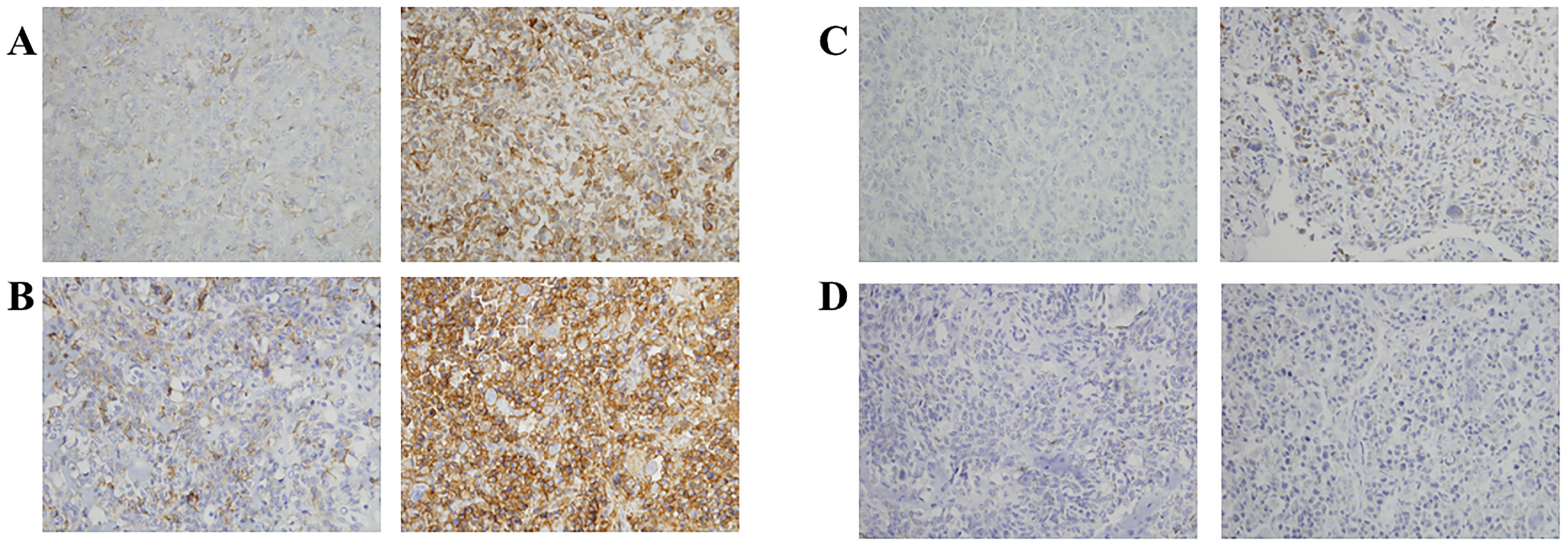
Immunohistochemistry of paired canine primary (left) and metastatic (right) in two different patient samples showing the expression of (A-B) CD44 and (C-D) CD147. p values for A and B p < 0.05 with metastatic > primary. p values for C p < 0.01 with metastatic > primary. Details of quantification are indicated in SI.

The expression of these proteins was finally tested in paired primary and metastatic OS canine tissue samples to investigate whether the findings in cell lines corresponds with spontaneously-arising osteosarcoma tumors in pet dogs. For each antibody to be tested two distinct sets of paired primary and metastatic tissue samples were evaluated for PM protein expressions. Furthermore, five sections of each tissue were stained to account for tissue heterogeneity. As observed in Fig 6A and 6B, for CD44 all the metastatic samples showed significantly higher staining intensity than their primary counterparts (A and B: p < 0.05). For CD147, the staining intensities in the patient samples also followed the trend as that of the cell lines. While samples from patient C did show higher CD147 expression in the metastatic (p<0.01), samples D showed metastatic staining greater than non-metastatic with p = 0.01. The quantification is shown in Fig H in S1 File.

## Discussion

The discovery of biomarkers and targetable receptors in osteosarcoma is key to improving therapeutic outcomes of metastatic OS disease. Herein we use high throughput peptide fingerprinting to identify potential protein level differences between metastatic and non-metastatic OS. While traditional investigations in biomarker discovery have focused on the comparison of tumorigenic versus non-tumorigenic samples, this study investigates the more subtle differences between metastatic and non-metastatic samples. Metastasis is a major challenge in OS therapy since non-metastatic OS can be treated by limb-sparing surgeries but metastatic disease has poor five-year survival rates and is often fatal. Thus, identifying metastasis biomarkers for potential therapeutics is of great importance. Additionally, the subtle changes in protein expression profile between metastatic and non-metastatic cells, provides a deeper insights into the factors that are responsible for promoting the metastasis of OS.

Furthermore, we investigated the proteome of metastatic and non-metastatic canine OS cellular membrane and compared it to the proteome of human OS cellar membrane. Canine OS is spontaneously arising and could serve as closer animal model for human OS than mice in which the disease has to be induced. Additionally, a similar trend in metastasis biomarkers for both species can help advance parallel development of therapeutics. Furthermore, our cross-species study lends more support to the differentially regulated protein species that are identified through the peptide fingerprinting studies.

The high-throughput capacity of proteomics method allows analysis of targets more rapidly than traditional biochemical methods like western blotting and flow cytometry. Herein, the semi-quantitative peptide fingerprinting method provided us with several protein candidates that were differentially expressed in the metastatic versus non-metastatic OS cell lines. While, proteomic approaches have been utilized for biomarker discovery in various cancers, its use has been limited in OS due to difficulty in protein collection from bone tissues. To circumvent this problem, a few studies have performed peptide fingerprinting on cell lines, comparing tumor cells versus osteoblasts [6,7,40]. These studies have provided valuable insight into the membrane proteome of OS cells and identified key biomarkers including NDRG1 and EPHA2. In this study, the membrane proteome of metastatic and non-metastatic OS cells were subject to peptide fingerprinting.

There are several important steps in metastasis including uncontrolled proliferation, motility, invasion and survival in vasculature that are mediated by interaction of signaling molecules with membrane proteins. Membrane proteins are known to be differentially regulated in cancerous states [41]. Membrane proteins are important components of the cell and form the largest class of drug targets. They form the gateway of the cells and control the influx and efflux of multiple signaling molecules. They are responsible for multiple functions including adhesion, cellular remodeling, proliferation and migration, several of which are significantly altered between normal and tumorigenic cells. Membrane proteins can be classified into various major classes including structural proteins, GPCRs, receptors (not including GPCRs), transport proteins, proteins responsible for immune responses and other smaller classes like enzymes.

GPCRs are a large class of PM receptors. The aberrant overexpression of GPCRs and their autocrine and paracrine activation by agonists released by tumor cells is one of the most frequent strategies used by cancer cells to develop and maintain their metastatic phenotype. While GPCRs constitute over 50% of current drug targets [42], the GPCRs targeted for cancer therapy are very few [43]. Various GPCRs including KRAS have shown to dysregulate the MAPK pathway and result in uncontrolled proliferation [44]. In our studies, in both species the metastatic cell lines showed an overall increase in GPCR content and this was also reflected in our GPCR activity assay. In this assay, the Gs class of receptors when stimulated by forskolin showed heightened cAMP production for both metastatic cell lines as compared to the non-metastatic cells.

Furthermore, proteins that formed an essential component of the ECM such as collagen were found to be differentially regulated, as degradation of the ECM and cell motility is a key step in the metastatic process. Overall collagens increased in both metastatic cell lines. Collagen regulates extracellular martrix (ECM) remodeling through matrix metalloproteinases (MMPs) [45]. It has been shown that in OS, the synthesis and activation of MMP-2 is affected by interactions between OS cells and collagen I. Proteins that promote the invasion of the vasculature, for example integrin β1, were also identified as upregulated. Interestingly, in OS, integrin α2β1 has shown to be correlated with increased invasion through its interaction with collagen 1 [46]. In the same study, cells forced to overexpress the α2 subunit was not sufficient to increase tumorigenesis implicating the involvement of the β1 subunit [46].

Proteins involved in the degradation of ECM, CD147 and MCT1, were also found to be upregulated in the metastatic cell lines. CD147 expression in the various cell lines was also validated through downstream biochemical techniques. Although no drugs are clinically available for targeting CD147, progress has been made towards the use of siRNA in targeting CD147 in human malignant melanoma [47].

Finally, a downregulation of immune complexes was observed in the metastatic cell lines as compared to the non-metastatic cell lines was also observed in our results. As cancerous cells try to evade the immune response, there is downregulation of the major histocompatibility complex. Lower expressions of MHCs, specifically MHC class I, have indicated less-favorable prognoses of various cancers [48].

In this work, we validated three proteins from different functional classes, by western blotting, flow cytometry, confocal microscopy and cell pellet IHC, as a representative set to show that results from proteomics hold true in low-throughput biochemical methods. CD147 has been discussed with respect to its importance in the metastatic process as well as possible therapeutic routes. CD44 and vimentin were the other two proteins which were significantly upregulated in both human and canine metastatic cells as compared to their non-metastatic counterparts. CD44 is a cell surface glycoprotein that has been implicated in various cancers. It is hypothesized that the poor prognosis to increased chemoresistance as mediated with its interaction with its ligand hyaluronic acid. However, the correlation of CD44 expression to prognosis and disease outcome has not been clearly established. Whereas in renal cell carcinoma a high expression level of CD44 is related to poor outcome [49], in ovarian cancer overexpression of CD44 is related to favorable prognosis [50]. Moreover, in breast cancer CD44 has been related to both tumor suppression and promotion [51]. Vimentin overexpression, on the other hand, is a marker of epithelial to mesenchymal transition in cancers of epithelial origin, like breast cancer. However, in sarcomas, the role of vimentin as a tumor marker has not been explored deeply due to its ubiquitous expression. Recent work has shown that Withaferin A targets vimentin in soft tissue sarcomas resulting in vimentin cleavage and apoptosis [52], increasing the promise for vimentin as a target in cancers of mesenchymal origin.

Finally, the results were validated in spontaneously arising osteosarcoma tumors in dog. For these studies, paired primary and lung metastasized samples from canine patients were utilized. The importance of using paired samples is to avoid any differences due to genetic differences in results. The results obtained show that *in vitro* cell culture proteomic studies followed by validation did correspond with the expression levels in canine patient samples. The CD44 results agreed very well with our small set of patient samples, the staining of CD147 in patient samples was not as intense as that in cell lines, although it was upregulated in the metastatic tumor as compared to the primary tumor.

This work is a proteomic profiling of human and canine membrane proteome and indicates that the upregulation of specific proteins follows the same trend. This has been verified in a limited number of canine paired primary and metastatic samples. However future work will focus on the verification of these targets, and other targets in a greater number of paired primary and metastatic canine tissues as well as human tissue samples. While it is anticipated that the upregulation will not be the same fold across all samples, the trends are expected to remain constant.

While cell lines only represent one part of the tumor microenvironment, tumors themselves present a significant level of heterogeneity. This microenvironment is also responsible for tumor development and progression through the interaction of the ECM, signaling molecules, immune system and other cell populations. The modeling of such environments to investigate their effects on the proteomic signature of cancer cells has been a subject of recent interest [53]. It is likely that these interaction will alter the proteomic profile of the tumor cells in both primary as well as metastatic tumors. Conversely, the presence of the tumor will also affect the environment and biomarker discovery from tumor microenvironment has also been explored [54].

To further explore the proteomic profile of cancer cells in orthotopic models, the study can be extended to animal models of the disease. While murine models are not the best models for osteosarcoma, since they do not develop spontaneous tumor and tumors have to be induced, they do provide a platform for testing out preliminary hypotheses. The cell lines used in this study have been previously shown to be a clinically relevant orthotopic model for human osteosarcoma because their tumor progression and metastasis development in mice, mimic the clinical scenario [11]. These studies will provide more evidence for the studies to be extended to companion animals that show more similar proteomic profile as humans, such as dogs, and will allow for a parallel development of therapeutics for both species.

## Conclusions

This study describes a cross-species comparison of PM proteins of metastatic and non-metastatic OS. We have shown that the global membrane proteomes of canine and human OS show significant preferential regulation of various proteins, which agrees with results in genetic expression levels. Furthermore, the cross-species comparison allows greater confirmation that dogs can be utilized as a model for human OS. It also offers a platform for the development of therapeutics for both species. We identified various differentially regulated proteins that were validated downstream by biochemical techniques including western blotting, flow cytometry and confocal microscopy. CD44, CD147 and vimentin were identified as significantly upregulated in both human and canine OS. The results were also confirmed by IHC in spontaneously arising osteosarcoma tumors samples.

## Supporting information

S1 File (DOCX)
