# Supplementary material for: Comparative proteomic investigation of metastatic and non-metastatic osteosarcoma cells of human and canine origin
Source: PLoS One. 2017 Sep 14;12(9):e0183930. doi: 10.1371/journal.pone.0183930 (PMC5598957; doi:10.1371/journal.pone.0183930)
Supplement: S1 File — (DOCX) [file pone.0183930.s001.docx]

Supporting Information

**Comparative Proteomic Investigation of Metastatic and Non-metastatic Osteosarcoma Cells of Human and Canine Origin**

Jahnabi Roy^1^, Kathryn L. Wycislo^2^, Holly Pondenis^3^, Timothy M. Fan^3^* and Aditi Das^4*^

^1^ Department of Chemistry, University of Illinois Urbana–Champaign, Urbana IL 61802, USA

^2^ Department of Pathobiology, University of Illinois Urbana–Champaign, Urbana IL 61802, USA

^3^ Department of Veterinary Clinical Medicine, University of Illinois Urbana–Champaign, Urbana IL 61802, USA

^4^ Department of Comparative Biosciences, Department of Biochemistry, Beckman Institute for Advanced Science, Division of Nutritional Sciences, Neuroscience Program and Department of Bioengineering, University of Illinois Urbana–Champaign, Urbana IL 61802, USA

*Corresponding author: Aditi Das, Timothy M. Fan

Email: [aditidas@illinois.edu](mailto:aditidas@illinois.edu) (AD), [t-fan@illinois.edu](mailto:t-fan@illinois.edu) (TMF)

**Table of Contents**

| Fig A: Workflow for proteomic analysis |  | |
| --- | --- | --- |
| Parameters for Proteomics Classifications |  | |
| Table TABLE SA-1 – TABLE SA-9 Classification of HOS cell membrane |  | |
| Table TABLE SB-1 – TABLE SB-9 Classification of 143B cell membrane |  | |
| Table TABLE SC-1 – TABLE SC-9 Classification of POS cell membrane |  | |
| Table TABLE SD-1 – TABLE SD-9 Classification of HMPOS cell membrane |  | |
| Confocal Microscopy Image Parameters |  | |
| Fig B- Relative expression of structural proteins in metastatic versus non- metastatic cells |  | |
| Fig C- Relative expression of immune complexes and immunoglobulins in metastatic versus non- metastatic cells | |  |
| Fig D- Ponceau staining as loading control for western blot |  | |
| Fig E- Western blot showing overexpression of KRAS in 143B vs HOS |  | |
| Fig F – Quantification of confocal microscopy images |  | |
| Fig G– Quantification of cell pellet IHC |  | |
| Fig H– Quantification of paired metastatic and non-metastatic IHC |  | |

**
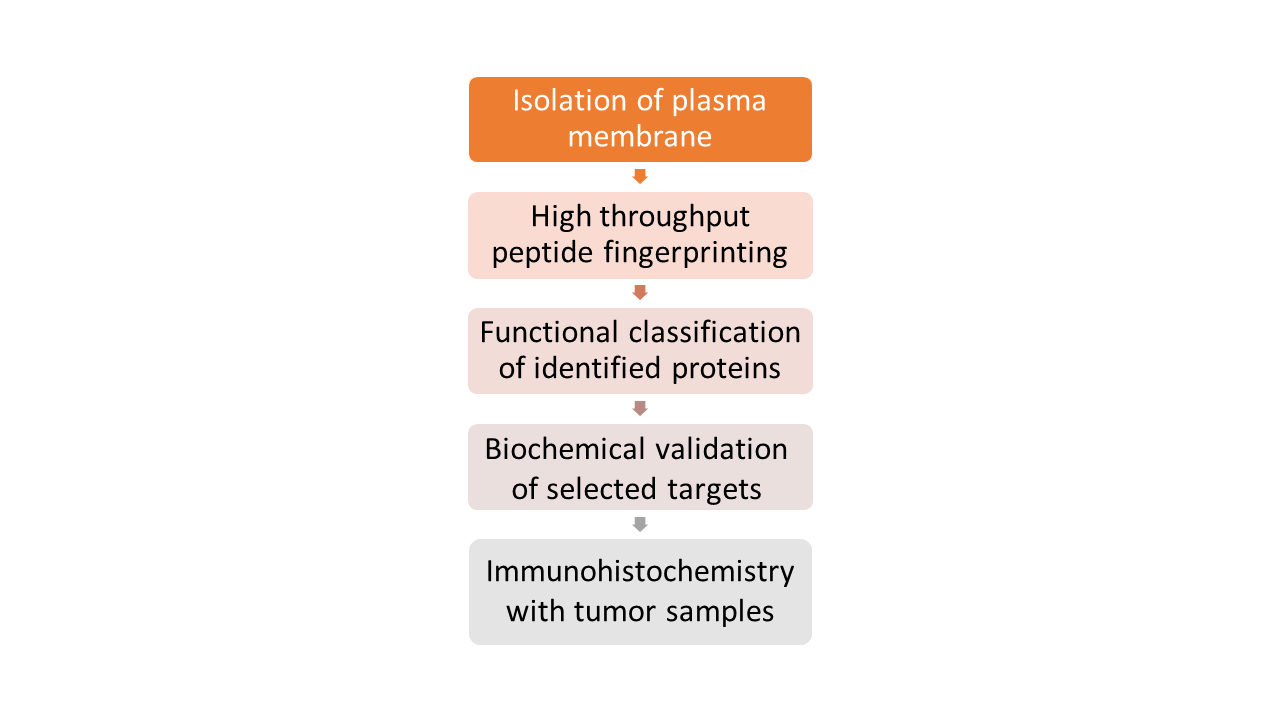
**

**Fig A:** Overall workflow for analysis of global membrane proteome of Osteosarcoma. Membrane fraction from OS cells was isolated and subjected to high-throughput peptide fingerprinting. The protein candidates were classified by function and selected protein targets were validated first biochemically and then in paired primary and metastatic canine tumor samples.

**Tables**

**Parameters for Proteomics Classifications**

| Taxonomy | Homo sapiens (human) (287,757 sequences) |
| --- | --- |
| Type of search | MS/MS Ion Search |
| Enzyme | Trypsin |
| Variable modifications | Oxidation (M) |
| Mass values | Monoisotopic |
| Protein mass | Unrestricted |
| Peptide mass tolerance | 0.3 Da |
| Fragment mass tolerance | 0.3 Da |
| Max missed cleavages | 3 |
| Instrument type | ESI-TRAP |
| Number of queries | 8,371 |
| Significance threshold p< | 0.05 |
| Max. number of families | AUTO |
| Ions score or expect cut-off | 0 |

**emPAI** or exponentially modified protein abundance index is a parameter indicating relative abundance of proteins in the mixture

**Table SA: Proteomics data of plasma membrane protein from HOS**

**
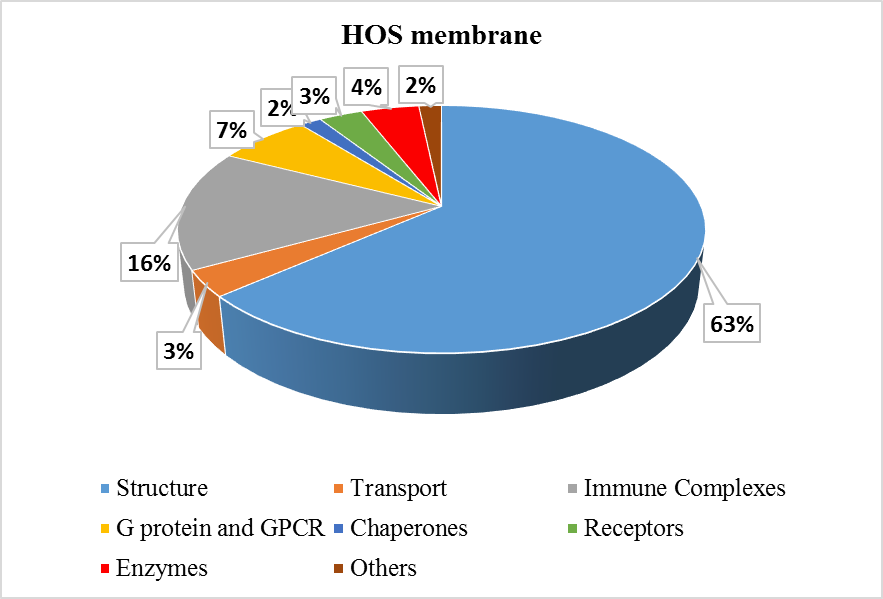
**

**Table SA-1. Overall proteomic profile**

|  | **emPAI** | **Percentage** |
| --- | --- | --- |
| Structure | 67.86 | 63.48 |
| Transport | 3.65 | 3.41 |
| Immune Complexes | 16.77 | 15.69 |
| G protein and GPCR | 7.21 | 6.74 |
| Chaperones | 1.71 | 1.60 |
| Receptors | 3.41 | 3.19 |
| Enzymes | 4.52 | 4.23 |
| Others | 1.77 | 1.66 |
| **Total** | **106.9** | **100** |

**Table SA-2: Structural Proteins Identified**

| **Accession** | **Mass** | **emPAI** | **Description** |
| --- | --- | --- | --- |
| **gi\|11935049** | 66027 | 2.07 | keratin 1 [Homo sapiens] |
| **gi\|119617057** | 57772 | 0.83 | keratin 8, isoform CRA_a [Homo sapiens] |
| **gi\|119617032** | 59874 | 0.44 | keratin 6B, isoform CRA_a [Homo sapiens] |
| **gi\|119395754** | 62340 | 0.32 | keratin, type II cytoskeletal 5 [Homo sapiens] |
| **gi\|119581148** | 57526 | 1.3 | keratin 9 (epidermolytic palmoplantar keratoderma) [Homo sapiens] |
| **gi\|12653819** | 48003 | 1.26 | Keratin 18 [Homo sapiens] |
| **gi\|1195531** | 51206 | 0.4 | type I keratin 16 [Homo sapiens] |
| **gi\|181402** | 65825 | 0.7 | epidermal cytokeratin 2 [Homo sapiens] |
| **gi\|7106439** | 49639 | 1.2 | tubulin beta-5 chain [Mus musculus] |
| **gi\|119608775** | 48794 | 1.04 | tubulin, beta 2C, isoform CRA_b [Homo sapiens] |
| **gi\|340021** | 50120 | 0.68 | alpha-tubulin [Homo sapiens] |
| **gi\|62421087** | 11517 | 0.44 | actin-like protein [Homo sapiens] |
| **gi\|4501885** | 41710 | 9.89 | actin, cytoplasmic 1 [Homo sapiens] |
| **gi\|63055057** | 41976 | 1.53 | beta-actin-like protein 2 [Homo sapiens] |
| **gi\|24119203** | 29015 | 0.56 | tropomyosin alpha-3 chain isoform 2 [Homo sapiens] |
| **gi\|17986258** | 16919 | 1.13 | myosin light polypeptide 6 isoform 1 [Homo sapiens] |
| **gi\|5031573** | 47341 | 0.2 | actin-related protein 3 isoform 1 [Homo sapiens] |
| **gi\|2605594** | 19694 | 0.55 | myosin regulatory light chain [Homo sapiens] |
| **gi\|5031595** | 19654 | 0.55 | actin-related protein 2/3 complex subunit 4 isoform a [Homo sapiens] |
| **gi\|4826659** | 30609 | 0.15 | F-actin-capping protein subunit beta isoform 1 [Homo sapiens] |
| **gi\|530417302** | 104260 | 0.23 | PREDICTED: alpha-actinin-4 isoform X1 [Homo sapiens] |
| **gi\|194097350** | 105502 | 0.23 | alpha-actinin-1 isoform a [Homo sapiens] |
| **gi\|167887751** | 49623 | 6.48 | vimentin variant 3 [Homo sapiens] |
| **gi\|12667788** | 226392 | 0.02 | myosin-9 [Homo sapiens] |
| **gi\|4757756** | 38580 | 19.39 | annexin A2 isoform 2 [Homo sapiens] |
| **gi\|157833780** | 35984 | 3.78 | Chain A, Human Annexin V With Proline Substitution By Thioproline |
| **gi\|71773329** | 75826 | 0.99 | annexin A6 isoform 1 [Homo sapiens] |
| **gi\|119582952** | 40057 | 0.72 | annexin A1, isoform CRA_c [Homo sapiens] |
| **gi\|53791219** | 277332 | 0.21 | filamin A [Homo sapiens] |
| **gi\|105990514** | 277990 | 0.07 | filamin-B isoform 2 [Homo sapiens] |
| **gi\|34234** | 31774 | 0.5 | laminin-binding protein, partial [Homo sapiens] |
| **gi\|187609338** | 20107 | 1.34 | Chain A, Crystal Structure Of The Extracellular Portion Of Hab18gCD147 |
| **gi\|119625804** | 66564 | 0.92 | moesin, isoform CRA_b [Homo sapiens] |
| **gi\|16974825** | 8483 | 0.63 | Chain A, Solution Structure Of Calcium-calmodulin N-terminal Domain |
| **gi\|7656991** | 53215 | 0.18 | coronin-1C isoform b [Homo sapiens] |
| **gi\|19920317** | 65983 | 0.3 | cytoskeleton-associated protein 4 [Homo sapiens] |
| **gi\|4826898** | 15045 | 1.32 | profilin-1 [Homo sapiens] |
| **gi\|3212355** | 11064 | 3.53 | Chain A, P11 (s100a10), Ligand Of Annexin Ii |
|  |  |  |  |
| **gi\|641958** | 228798 | 0.02 | non-muscle myosin B [Homo sapiens] |
| **gi\|15778914** | 42189 | 0.11 | Parvin, alpha [Homo sapiens] |
| **gi\|5031601** | 40923 | 0.11 | actin-related protein 2/3 complex subunit 1B [Homo sapiens] |
| **gi\|3676059** | 231477 | 0.02 | envoplakin [Homo sapiens] |
| **gi\|578798330** | 64316 | 0.07 | PREDICTED: afadin- and alpha-actinin-binding protein isoform X6 [Homo sapiens] |
| **gi\|44889481** | 124872 | 0.04 | unconventional myosin-Ib isoform 2 [Homo sapiens] |
| **gi\|1846005** | 332987 | 0.01 | collagen type XII alpha-1 [Homo sapiens] |
| **gi\|119585235** | 84713 | 0.05 | SWI/SNF related, matrix associated, actin dependent regulator of chromatin, subfamily c, member 1, isoform CRA_a [Homo sapiens] |
| **gi\|4235275** | 269661 | 0.02 | talin [Homo sapiens] |
| **gi\|306896** | 57790 | 0.08 | intercellular adhesion molecule-1 precursor [Homo sapiens] |
| **gi\|119584665** | 9744 | 0.53 | ankyrin repeat domain 28, isoform CRA_d [Homo sapiens] |
| **gi\|119587605** | 119373 | 0.04 | neural cell adhesion molecule 1, isoform CRA_a [Homo sapiens] |
| **gi\|530421342** | 193067 | 0.02 | PREDICTED: FERM and PDZ domain-containing protein 4 isoform X1 [Homo sapiens] |
| **gi\|6912534** | 29088 | 0.16 | BMP and activin membrane-bound inhibitor homolog precursor [Homo sapiens] |
| **gi\|50897294** | 51245 | 0.09 | POTE ankyrin domain family member A isoform 1 [Homo sapiens] |
| **gi\|14149805** | 38735 | 0.12 | enkurin domain-containing protein 1 [Homo sapiens] |
| **gi\|4503375** | 56594 | 0.08 | dihydropyrimidinase [Homo sapiens] |
| **gi\|29801** | 53563 | 0.18 | CD44E (epithelial form) [Homo sapiens] |
| **gi\|119598259** | 132309 | 0.03 | uveal autoantigen with coiled-coil domains and ankyrin repeats, isoform CRA_a [Homo sapiens] |

**TABLE SA-3: Transport Proteins Identified**

| **Accession** | **Mass** | **emPAI** | **Description** |
| --- | --- | --- | --- |
| **gi\|530396432** | 69258 | 0.06 | PREDICTED: two pore calcium channel protein 2 isoform X4 [Homo sapiens] |
| **gi\|62088088** | 85811 | 0.94 | transferrin receptor variant [Homo sapiens] |
| **gi\|119574954** | 34459 | 0.65 | voltage-dependent anion channel 2, isoform CRA_a [Homo sapiens] |
| **gi\|190133** | 134625 | 0.07 | plasma membrane Ca2+ pumping ATPase [Homo sapiens] |
| **gi\|1478281** | 56585 | 0.17 | neutral amino acid transporter B [Homo sapiens] |
| **gi\|119595679** | 95691 | 0.05 | potassium voltage-gated channel, KQT-like subfamily, member 2, isoform CRA_c [Homo sapiens] |
| **gi\|115583685** | 53909 | 0.08 | monocarboxylate transporter 1 [Homo sapiens] |
| **gi\|119626956** | 136757 | 0.03 | cache domain containing 1 [Homo sapiens] |
| **gi\|98986321** | 58920 | 0.08 | gliomedin [Homo sapiens] |
| **gi\|238427** | 30623 | 0.15 | Porin 31HM [human, skeletal muscle membranes, Peptide, 282 aa] |
| **gi\|222143239** | 13223 | 0.38 | Chain A, Crystal Structure Of The Px Domain Of Sorting Nexin-17 (Snx17) |
| **gi\|15012080** | 17190 | 0.28 | ACAT1 protein [Homo sapiens] |
| **gi\|549988** | 81597 | 0.05 | sulfate transporter [Homo sapiens] |
| **gi\|347948492** | 8529 | 0.62 | Chain A, Complex Of Cambr And Cam |
| **gi\|3882215** | 119783 | 0.04 | KIAA0747 protein [Homo sapiens] |

**TABLE SA-4: Immune Complexes Identified**

| **Accession** | **Mass** | **emPAI** | **Description** |
| --- | --- | --- | --- |
| **gi\|161376703** | 31480 | 0.51 | MHC class I antigen [Homo sapiens] |
| **gi\|47564005** | 31489 | 0.73 | MHC class I antigen [Homo sapiens] |
| **gi\|358423242** | 21090 | 0.84 | MHC class I antigen [Homo sapiens] |
| **gi\|333036593** | 21139 | 0.84 | MHC class I antigen [Homo sapiens] |
| **gi\|34222512** | 40410 | 0.54 | RecName: Full=HLA class I histocompatibility antigen, B-73 alpha chain; AltName: Full=MHC class I antigen B*73; Flags: Precursor |
| **gi\|55415712** | 10828 | 0.47 | MHC class II antigen [Homo sapiens] |
| **gi\|371448219** | 14569 | 0.34 | immunoglobulin G heavy chain variable region, partial [Homo sapiens] |
| **gi\|26801098** | 8458 | 0.63 | immunoglobulin heavy chain variable region [Homo sapiens] |
| **gi\|371447513** | 13673 | 0.36 | immunoglobulin G heavy chain variable region, partial [Homo sapiens] |
| **gi\|62871150** | 17043 | 0.28 | immunoglobulin alpha heavy chain variable region [Homo sapiens] |
| **gi\|304562592** | 13044 | 0.38 | immunoglobulin gamma 1 heavy chain variable region [Homo sapiens] |
| **gi\|388777938** | 13723 | 0.36 | immunoglobulin heavy chain variable region, partial [Homo sapiens] |
| **gi\|177216** | 58041 | 0.16 | 4F2 heavy chain antigen [Homo sapiens] |
| **gi\|179531** | 26172 | 0.18 | IgE-binding protein [Homo sapiens] |
| **gi\|220702506** | 54199 | 0.9 | Chain A, TapasinERP57 HETERODIMER |
| **gi\|7161035** | 10423 | 0.49 | immunoglobulin heavy chain [Homo sapiens] |
| **gi\|886258** | 65091 | 0.07 | alcam [Homo sapiens] |
| **gi\|19909527** | 38169 | 0.57 | DERP12 (dermal papilla derived protein 12) [Homo sapiens] |
| **gi\|19747283** | 12346 | 0.41 | X antigen family member 2 [Homo sapiens] |
| **gi\|627493** | 2788 | 2.82 | interferon alpha (component g) - human (fragment) |
| **gi\|70913399** | 2021 | 4.89 | T cell receptor alpha variable 7 [Homo sapiens] |

**TABLE SA-5: GPCRs and G proteins**

| **Accession** | **Mass** | **emPAI** | **Description** |
| --- | --- | --- | --- |
| **gi\|4504041** | 40425 | 0.38 | guanine nucleotide-binding protein G(i) subunit alpha-2 isoform 1 [Homo sapiens] |
| **gi\|119574084** | 39680 | 0.39 | guanine nucleotide binding protein (G protein), beta polypeptide 2-like 1, isoform CRA_h [Homo sapiens] |
| **gi\|297660220** | 16459 | 0.3 | Rho GTPase activating protein 26 variant 3 [Homo sapiens] |
| **gi\|1770396** | 42266 | 0.11 | G-protein coupled receptor (putative) [Homo sapiens] |
| **gi\|6680045** | 37353 | 0.12 | guanine nucleotide-binding protein G(I)/G(S)/G(T) subunit beta-1 [Mus musculus] |
| **gi\|1174072** | 42116 | 0.23 | G alpha-q [Homo sapiens] |
| **gi\|52353947** | 34995 | 0.13 | olfactory receptor 51F1 [Homo sapiens] |
| **gi\|187281** | 77555 | 0.4 | M4 protein [Homo sapiens] |
| **gi\|540344584** | 37576 | 0.12 | guanine nucleotide-binding protein subunit alpha-12 isoform 3 [Homo sapiens] |
| **gi\|62088744** | 29577 | 0.16 | regulator of G-protein signalling 11 isoform 1 variant [Homo sapiens] |
| **gi\|4758796** | 40517 | 0.11 | developmentally-regulated GTP-binding protein 1 [Homo sapiens] |
| **gi\|119626208** | 51201 | 0.09 | septin 11, isoform CRA_a [Homo sapiens] |
| **gi\|58257741** | 171369 | 0.03 | KIAA1219 protein [Homo sapiens] |
| **gi\|4505675** | 68448 | 0.07 | high affinity cGMP-specific 3',5'-cyclic phosphodiesterase 9A isoform a [Homo sapiens] |
| **gi\|4456467** | 77101 | 0.06 | TM7XN1 protein [Homo sapiens] |
| **gi\|3002951** | 15512 | 0.31 | breakpoint cluster region protein 1 [Homo sapiens] |
| **gi\|71296783** | 76213 | 0.06 | RAP1GAP protein [Homo sapiens] |
| **gi\|119608392** | 123307 | 0.04 | Rap guanine nucleotide exchange factor (GEF) 1, isoform CRA_b [Homo sapiens] |
| **gi\|21928311** | 35019 | 0.13 | seven transmembrane helix receptor [Homo sapiens] |
|  |  |  |  |
| **gi\|13569962** | 22157 | 1.17 | ras-related protein Rab-1B [Homo sapiens] |
| **gi\|119620329** | 28129 | 0.85 | RAB1A, member RAS oncogene family, isoform CRA_f [Homo sapiens] |
| **gi\|4506413** | 20974 | 0.5 | ras-related protein Rap-1A precursor [Homo sapiens] |
| **gi\|20147713** | 23971 | 0.43 | Ras family small GTP binding protein RALA [Homo sapiens] |
| **gi\|5031703** | 52132 | 0.09 | ras GTPase-activating protein-binding protein 1 [Homo sapiens] |
| **gi\|141797011** | 189162 | 0.07 | IQ motif containing GTPase activating protein 1 [Homo sapiens] |
| **gi\|508285** | 23553 | 0.44 | Rab5c-like protein, similar to Canis familiaris Rab5c protein, PIR Accession Number S38625 [Homo sapiens] |
| **gi\|763130** | 24559 | 0.19 | YPT3 [Homo sapiens] |
| **gi\|359807059** | 20502 | 0.23 | TBC1 domain family member 1 isoform 4 [Homo sapiens] |

**TABLE SA-6: Chaperone Proteins Identified**

| Accession | Mass | emPAI | Description |
| --- | --- | --- | --- |
|  |  |  |  |
| gi\|431822408 | 82269 | 0.89 | heat shock protein HSP 90-beta isoform c [Homo sapiens] |
| gi\|153792590 | 98099 | 0.43 | heat shock protein HSP 90-alpha isoform 1 [Homo sapiens] |
| gi\|4502643 | 57988 | 0.08 | T-complex protein 1 subunit zeta isoform a [Homo sapiens] |
| gi\|5453603 | 57452 | 0.08 | T-complex protein 1 subunit beta isoform 1 [Homo sapiens] |
| gi\|48762932 | 59583 | 0.08 | T-complex protein 1 subunit theta isoform 1 [Homo sapiens] |
| gi\|48145555 | 59291 | 0.08 | CCT7 [Homo sapiens] |
| gi\|671527 | 60292 | 0.07 | gamma subunit of CCT chaperonin [Homo sapiens] |

**TABLE SA-7: Receptors Identifed**

| Accession | Mass | emPAI | Description |
| --- | --- | --- | --- |
| gi\|19743813 | 88357 | 0.22 | integrin beta-1 isoform 1A precursor [Homo sapiens] |
| gi\|119581591 | 46372 | 0.45 | basigin (Ok blood group), isoform CRA_g [Homo sapiens] |
| gi\|119610286 | 18642 | 1.5 | progesterone receptor membrane component 1, isoform CRA_b [Homo sapiens] |
| gi\|62897779 | 62485 | 0.07 | thyroid hormone receptor interactor 10 variant [Homo sapiens] |
| gi\|291621647 | 26154 | 0.18 | membrane-associated progesterone receptor component 2 [Homo sapiens] |
| gi\|62089372 | 30214 | 0.15 | FK506 binding protein 5 variant [Homo sapiens] |
| gi\|483831 | 303844 | 0.01 | type 3 inositol 1,4,5-trisphosphate receptor [Homo sapiens] |
| gi\|472848 | 22094 | 0.21 | N-methyl-D-aspartate receptor subunit, partial [Homo sapiens] |
| gi\|703110 | 17307 | 0.28 | thyroid receptor interactor, partial [Homo sapiens] |
| gi\|62088916 | 265943 | 0.02 | Insulin-like growth factor 2 receptor variant [Homo sapiens] |
| gi\|62089376 | 70414 | 0.06 | complement component 1, q subcomponent, receptor 1 variant [Homo sapiens] |
| gi\|15192139 | 19666 | 0.24 | porimin [Homo sapiens] |
| gi\|578831638 | 204161 | 0.02 | PREDICTED: peripheral-type benzodiazepine receptor-associated protein 1 isoform X3 [Homo sapiens] |

**TABLE SA-8: Enzymes Identified**

| Accession | Mass | emPAI | Description |
| --- | --- | --- | --- |
| gi\|33946291 | 59113 | 0.08 | lysophosphatidylcholine acyltransferase 1 [Homo sapiens] |
| gi\|291463382 | 17983 | 1.58 | Chain A, Free Acetyl-Cypa Trigonal Form |
| gi\|181250 | 22597 | 1.13 | cyclophilin, partial [Homo sapiens] |
| gi\|33946291 | 59113 | 0.08 | lysophosphatidylcholine acyltransferase 1 [Homo sapiens] |
| gi\|6331328 | 115103 | 0.08 | KIAA1280 protein [Homo sapiens] |
| gi\|530390528 | 71082 | 0.06 | PREDICTED: carnitine O-acetyltransferase isoform X1 [Homo sapiens] |
| gi\|119596301 | 24546 | 0.42 | tyrosine 3-monooxygenase/tryptophan 5-monooxygenase activation protein, beta polypeptide, isoform CRA_b [Homo sapiens] |
| gi\|693933 | 47079 | 0.32 | 2-phosphopyruvate-hydratase alpha-enolase [Homo sapiens] |
| gi\|605604525 | 9922 | 0.53 | Chain A, Crystal Structure Of Phospholipase C Beta 3 In Complex With Pdz1 Of Nherf1 |
| gi\|38202211 | 143531 | 0.03 | N-acetylglucosamine-1-phosphotransferase subunits alpha/beta precursor [Homo sapiens] |
| gi\|570359824 | 37730 | 0.12 | Chain A, Crystal Structure Of The Human Cyclin G Associated Kinase (gak) |
| gi\|68051721 | 49031 | 0.09 | neutral cholesterol ester hydrolase 1 isoform b [Homo sapiens] |

**TABLE SA-9: Other Proteins Identified**

| Accession | Mass | emPAI | Description |
| --- | --- | --- | --- |
| gi\|1665773 | 78230 | 0.06 | KIAA0253 [Homo sapiens] |
| gi\|178775 | 28944 | 1.1 | proapolipoprotein, partial [Homo sapiens] |
| gi\|4505893 | 16680 | 0.29 | proteolipid protein 2 [Homo sapiens] |
| gi\|1374813 | 23368 | 0.2 | SNAP-23 [Homo sapiens] |
| gi\|28376621 | 46614 | 0.1 | SEC14-like protein 4 isoform a [Homo sapiens] |
| gi\|530393410 | 236464 | 0.02 | PREDICTED: myoferlin isoform X1 [Homo sapiens] |

**TABLE SB: Proteomics data of plasma membrane protein from 143B**

**
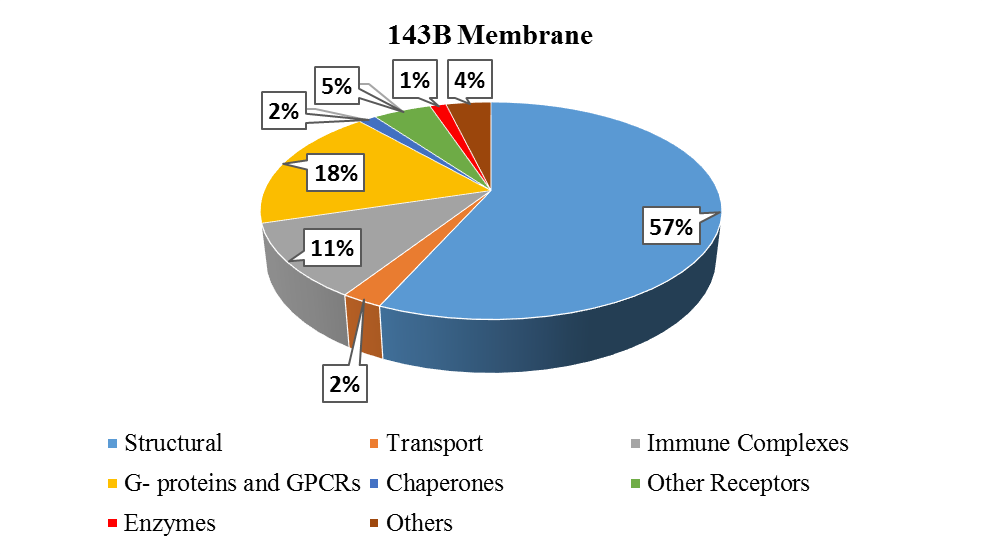
**

**TABLE SB-1. Overall proteomic profile**

|  | emPAI | Percentage (Equation SE-1) |
| --- | --- | --- |
| Structural | 87.73 | 56.68 |
| Transport | 3.81 | 2.46 |
| Immune Complexes | 17.16 | 11.09 |
| G- proteins and GPCRs | 28.33 | 18.30 |
| Chaperones | 2.27 | 1.47 |
| Other Receptors | 7.55 | 4.88 |
| Enzymes | 2.09 | 1.35 |
| Others | 5.84 | 3.77 |
| **Total** | **154.78** | **100** |

**TABLE SB-2. Structural proteins detected**

| Accession | Mass | emPAI | | Description |  |
| --- | --- | --- | --- | --- | --- |
| gi\|435476 | 62092 | 2.89 | cytokeratin 9 [Homo sapiens] | | |
| gi\|12803709 | 51619 | 1.26 | Keratin 14 [Homo sapiens] | | |
| gi\|12653819 | 48003 | 0.3 | Keratin 18 [Homo sapiens] | | |
| gi\|11935049 | 66027 | 6.72 | keratin 1 [Homo sapiens] | | |
| gi\|62414289 | 53619 | 9.57 | vimentin [Homo sapiens] | | |
| gi\|47132620 | 65393 | 2.88 | keratin, type II cytoskeletal 2 epidermal [Homo sapiens] | | |
| gi\|119617032 | 59874 | 0.76 | keratin 6B, isoform CRA_a [Homo sapiens] | | |
| gi\|119395754 | 62340 | 0.84 | keratin, type II cytoskeletal 5 [Homo sapiens] | | |
| gi\|119617057 | 57772 | 0.79 | keratin 8, isoform CRA_a [Homo sapiens] | | |
| gi\|28173564 | 58887 | 0.15 | keratin, type II cytoskeletal 73 [Homo sapiens] | | |
| gi\|73909156 | 40503 | 26.66 | Annexin A2 [Homo sapiens] | | |
| gi\|62897671 | 41694 | 8.17 | beta actin variant [Homo sapiens] | | |
| gi\|7106439 | 49639 | 1.77 | tubulin beta-5 chain [Mus musculus] | | |
| gi\|119608775 | 48794 | 1.81 | tubulin, beta 2C, isoform CRA_b [Homo sapiens] | | |
| gi\|157833780 | 35984 | 1.54 | Chain A, Human Annexin V With Proline Substitution By Thioproline | | |
| gi\|34234 | 31774 | 0.7 | laminin-binding protein, partial [Homo sapiens] | | |
| gi\|16974825 | 8483 | 10.06 | Chain A, Solution Structure Of Calcium-calmodulin N-terminal Domain | | |
| gi\|5031635 | 18491 | 2.07 | cofilin-1 [Homo sapiens] | | |
| gi\|4502101 | 38690 | 0.92 | annexin A1 [Homo sapiens] | | |
| gi\|12667788 | 226392 | 0.18 | myosin-9 [Homo sapiens] | | |
| gi\|24119203 | 29015 | 1.06 | tropomyosin alpha-3 chain isoform 2 [Homo sapiens] | | |
| gi\|17986258 | 16919 | 1.67 | myosin light polypeptide 6 isoform 1 [Homo sapiens] | | |
| gi\|179976 | 75857 | 0.48 | calelectrin [Homo sapiens] | | |
| gi\|37492 | 50126 | 0.52 | alpha-tubulin [Homo sapiens] | | |
| gi\|321400138 | 46537 | 0.31 | CD44 antigen isoform 6 precursor [Homo sapiens] | | |
| gi\|119593154 | 263754 | 0.08 | filamin A, alpha (actin binding protein 280), isoform CRA_e [Homo sapiens] | | |
| gi\|530393410 | 236464 | 0.07 | PREDICTED: myoferlin isoform X1 [Homo sapiens] | | |
| gi\|7656991 | 53215 | 0.08 | coronin-1C isoform b [Homo sapiens] | | |
| gi\|3282771 | 278018 | 0.06 | actin-binding protein homolog ABP-278 [Homo sapiens] | | |
| gi\|4826898 | 15045 | 0.73 | profilin-1 [Homo sapiens] | | |
| gi\|119584665 | 9744 | 0.52 | ankyrin repeat domain 28, isoform CRA_d [Homo sapiens] | | |
| gi\|186837 | 197937 | 0.02 | laminin B1 [Homo sapiens] | | |
| gi\|41322910 | 512292 | 0.03 | plectin isoform 1d [Homo sapiens] | | |
| gi\|205831092 | 68253 | 0.06 | RecName: Full=Putative IQ motif and ankyrin repeat domain-containing protein LOC642574 | | |
| gi\|641958 | 228798 | 0.02 | non-muscle myosin B [Homo sapiens] | | |
| gi\|3287188 | 127296 | 0.03 | ankyrin-like protein [Homo sapiens] | | |
| gi\|120660098 | 163133 | 0.03 | CAMSAP1 protein [Homo sapiens] | | |
| gi\|444738793 | 5129 | 1.16 | alternative protein CEP350 [Homo sapiens] | | |
| gi\|8885790 | 287292 | 0.03 | filamin 2 [Homo sapiens] | | |
| gi\|762885 | 81583 | 0.05 | Plakoglobin [Homo sapiens] | | |
| gi\|578821687 | 271519 | 0.02 | PREDICTED: spectrin beta chain, non-erythrocytic 2 isoform X3 [Homo sapiens] | | |
| gi\|438056 | 342550 | 0.01 | laminin M chain (merosin) [Homo sapiens] | | |
| gi\|4507115 | 54496 | 0.08 | fascin [Homo sapiens] | | |
| gi\|119576069 | 394840 | 0.01 | myosin XVA, isoform CRA_c [Homo sapiens] | | |
| gi\|6563228 | 43212 | 0.1 | rap2 interacting protein x [Homo sapiens] | | |
| gi\|4757944 | 25792 | 0.18 | CD81 antigen [Homo sapiens] | | |
| gi\|1107687 | 505963 | 0.02 | homologue of Drosophila Fat protein [Homo sapiens] | | |
| gi\|578837838 | 423562 | 0.01 | PREDICTED: dystrophin isoform X5 [Homo sapiens] | | |
| gi\|578823153 | 93390 | 0.05 | PREDICTED: tastin isoform X1 [Homo sapiens] | | |
| gi\|119574344 | 91877 | 0.05 | ankyrin repeat domain 30B, partial [Homo sapiens] | | |
| gi\|2996006 | 70660 | 0.06 | outer dense fiber protein 2/2 [Homo sapiens] | | |
| gi\|34226 | 288131 | 0.01 | laminin A chain [Homo sapiens] | | |
| gi\|530370381 | 134263 | 0.03 | PREDICTED: unconventional myosin-Ib isoform X2 [Homo sapiens] | | |
| gi\|186964 | 177492 | 0.02 | laminin B2 chain [Homo sapiens] | | |
| gi\|578819318 | 310817 | 0.01 | PREDICTED: ankyrin-3 isoform X17 [Homo sapiens] | | |
| gi\|55743096 | 193394 | 0.02 | collagen alpha-1(XIV) chain precursor [Homo sapiens] | | |

**TABLE SB-3: Transport Proteins detected**

| Accession | Mass | emPAI | Description |
| --- | --- | --- | --- |
| gi\|119574954 | 34459 | 0.84 | voltage-dependent anion channel 2, isoform CRA_a [Homo sapiens] |
| gi\|198443050 | 31818 | 0.3 | Chain A, Solution Structure Of Human Vdac-1 In Ldao Micelles |
| gi\|61744477 | 68059 | 0.28 | 4F2 cell-surface antigen heavy chain isoform b [Homo sapiens] |
| gi\|4505893 | 16680 | 0.28 | proteolipid protein 2 [Homo sapiens] |
| gi\|51094709 | 102523 | 0.04 | tweety homolog 3 (Drosophila) [Homo sapiens] |
| gi\|48255951 | 136789 | 0.03 | plasma membrane calcium-transporting ATPase 2 isoform 1 [Homo sapiens] |
| gi\|5733504 | 27630 | 0.16 | voltage-dependent anion channel VDAC3 [Homo sapiens] |
| gi\|1478281 | 56585 | 0.16 | neutral amino acid transporter B [Homo sapiens] |
| gi\|5730102 | 106258 | 0.04 | short transient receptor potential channel 6 [Homo sapiens] |
| gi\|4502281 | 31492 | 0.14 | sodium/potassium-transporting ATPase subunit beta-3 [Homo sapiens] |
| gi\|115583685 | 53909 | 0.17 | monocarboxylate transporter 1 [Homo sapiens] |
| gi\|9957467 | 269786 | 0.02 | ATP-binding cassette sub-family A member 2 [Homo sapiens] |
| gi\|38516 | 20468 | 0.23 | caveolin [Homo sapiens] |
| gi\|347948492 | 8529 | 0.61 | Chain A, Complex Of Cambr And Cam |
| gi\|62087606 | 50068 | 0.09 | sorting nexin 1 isoform a variant [Homo sapiens] |
| gi\|2337920 | 29827 | 0.15 | syntaxin 7 [Homo sapiens] |
| gi\|25777643 | 59949 | 0.07 | small conductance calcium-activated potassium channel protein 1 [Homo sapiens] |
| gi\|51339295 | 126843 | 0.07 | cation channel sperm-associated protein subunit beta precursor [Homo sapiens] |
| gi\|4507297 | 68692 | 0.06 | syntaxin-binding protein 1 isoform a [Homo sapiens] |
| gi\|169790839 | 59534 | 0.07 | excitatory amino acid transporter 1 isoform 1 [Homo sapiens] |

**TABLE SB-4 : Immune Complex proteins detected**

| Accession | Mass | emPAI | Description |
| --- | --- | --- | --- |
| gi\|432139040 | 12890 | 0.38 | immunoglobulin heavy chain variable region, partial [Homo sapiens] |
| gi\|290560013 | 11815 | 3.04 | Chain B, Crystal Structure Of Mhc Class I Hla-A2.1 Bound To A Photocleavable Peptide |
| gi\|4324088 | 10594 | 0.47 | immunoglobulin lambda light chain variable region [Homo sapiens] |
| gi\|307496 | 1390 | 9.07 | T cell receptor beta chain [Homo sapiens] |
| gi\|333036593 | 21139 | 0.22 | MHC class I antigen [Homo sapiens] |
| gi\|151188217 | 40719 | 0.11 | killer-cell Ig-like receptor [Homo sapiens] |
| gi\|17986005 | 29943 | 0.15 | major histocompatibility complex, class II, DR beta 3 precursor [Homo sapiens] |
| gi\|611962048 | 10753 | 0.46 | immunoglobulin heavy chain variable region, partial [Homo sapiens] |
| gi\|247425243 | 13679 | 0.35 | immunoglobulin heavy chain variable region [Homo sapiens] |
| gi\|15680023 | 27975 | 0.16 | B-cell receptor-associated protein 31 [Homo sapiens] |
| gi\|4038122 | 4086 | 1.57 | T-cell receptor beta chain [Homo sapiens] |
| gi\|226316189 | 21056 | 0.22 | MHC class I antigen [Homo sapiens] |
| gi\|224962016 | 21226 | 0.48 | MHC class II antigen [Homo sapiens] |
| gi\|119573139 | 39827 | 0.11 | SLAM family member 9, isoform CRA_a [Homo sapiens] |
| gi\|26985944 | 13046 | 0.37 | immunoglobulin IgG1 heavy chain [Homo sapiens] |
|  |  |  |  |

**TABLE SB- 5: GPCRs and G- proteins detected**

| Accession | Mass | emPAI | Description |
| --- | --- | --- | --- |
| gi\|4506413 | 20974 | 0.81 | ras-related protein Rap-1A precursor [Homo sapiens] |
| gi\|327195100 | 33961 | 0.45 | UBE2L3/KRAS fusion protein [Homo sapiens] |
| gi\|119574084 | 39680 | 0.53 | guanine nucleotide binding protein (G protein), beta polypeptide 2-like 1, isoform CRA_h [Homo sapiens] |
| gi\|508285 | 23553 | 0.43 | Rab5c-like protein, similar to Canis familiaris Rab5c protein, PIR Accession Number S38625 [Homo sapiens] |
| gi\|13569962 | 22157 | 1.56 | ras-related protein Rab-1B [Homo sapiens] |
| gi\|33946329 | 23552 | 0.43 | ras-related protein Ral-A precursor [Homo sapiens] |
| gi\|1174149 | 23447 | 1.04 | small GTP binding protein Rab7 [Homo sapiens] |
| gi\|14249144 | 24473 | 0.67 | ras-related protein Rab-11B [Rattus norvegicus] |
| gi\|5729850 | 40506 | 0.37 | guanine nucleotide-binding protein G(k) subunit alpha [Homo sapiens] |
| gi\|21361884 | 24199 | 0.19 | ras-related protein Rab-2B isoform 1 [Homo sapiens] |
| gi\|5031703 | 52132 | 0.18 | ras GTPase-activating protein-binding protein 1 [Homo sapiens] |
| gi\|5031703 | 52132 | 0.18 | ras GTPase-activating protein-binding protein 1 [Homo sapiens] |
| gi\|297660220 | 16459 | 0.29 | Rho GTPase activating protein 26 variant 3 [Homo sapiens] |
| gi\|311697329 | 3564 | 1.93 | KRAS protein [Homo sapiens] |
| gi\|4885287 | 7314 | 0.73 | guanine nucleotide-binding protein G(I)/G(S)/G(O) subunit gamma-5 precursor [Homo sapiens] |
| gi\|540344584 | 37576 | 0.12 | guanine nucleotide-binding protein subunit alpha-12 isoform 3 [Homo sapiens] |
| gi\|3292965 | 1071 | 15.8 | m1 muscarinic acetylcholine receptor protein [Homo sapiens] |
| gi\|53828729 | 34724 | 0.13 | olfactory receptor 7D2 [Homo sapiens] |
| gi\|334278900 | 35700 | 0.12 | olfactory receptor 4N4 [Homo sapiens] |
| gi\|19338916 | 36399 | 0.12 | G protein-coupled receptor SNSR5 [Homo sapiens] |
| gi\|2500069 | 21555 | 0.21 | RecName: Full=Ras-related protein Rab-31; AltName: Full=Ras-related protein Rab-22B [Homo sapiens] |
| gi\|3327062 | 221821 | 0.02 | KIAA0624 protein [Homo sapiens] |
| gi\|4139784 | 24393 | 0.19 | Chain A, Canine Gdp-Ran Q69l Mutant |
| gi\|390635651 | 110782 | 0.04 | ras-related protein Rab-44 [Homo sapiens] |
| gi\|21928341 | 34874 | 0.13 | seven transmembrane helix receptor [Homo sapiens] |
| gi\|530416765 | 113467 | 0.04 | PREDICTED: ras-interacting protein 1 isoform X1 [Homo sapiens] |
| gi\|62088744 | 29577 | 0.15 | regulator of G-protein signalling 11 isoform 1 variant [Homo sapiens] |
| gi\|359807059 | 20502 | 0.22 | TBC1 domain family member 1 isoform 4 [Homo sapiens] |
| gi\|4836765 | 77704 | 0.06 | G-protein-coupled receptor [Homo sapiens] |
| gi\|13122463 | 37605 | 0.12 | G protein-coupled receptor [Homo sapiens] |
| gi\|9625037 | 21295 | 0.22 | rho-related GTP-binding protein RhoG precursor [Mus musculus] |
| gi\|51036603 | 8001 | 0.66 | guanine nucleotide-binding protein G(I)/G(S)/G(O) subunit gamma-12 precursor [Homo sapiens] |
| gi\|15293749 | 23836 | 0.19 | olfactory receptor [Homo sapiens] |

**TABLE SB-6: Chaperone proteins detected**

| Accession | Mass | emPAI | Description |
| --- | --- | --- | --- |
| gi\|431822408 | 82269 | 0.95 | heat shock protein HSP 90-beta isoform c [Homo sapiens] |
| gi\|153792590 | 98099 | 0.47 | heat shock protein HSP 90-alpha isoform 1 [Homo sapiens] |
| gi\|62089036 | 57725 | 0.16 | chaperonin containing TCP1, subunit 6A isoform a variant [Homo sapiens] |
| gi\|5453603 | 57452 | 0.16 | T-complex protein 1 subunit beta isoform 1 [Homo sapiens] |
| gi\|1136741 | 58465 | 0.16 | KIAA0002 [Homo sapiens] |
| gi\|5453607 | 59329 | 0.15 | T-complex protein 1 subunit eta isoform a [Homo sapiens] |
| gi\|671527 | 60292 | 0.07 | gamma subunit of CCT chaperonin [Homo sapiens] |
| gi\|36796 | 60356 | 0.15 | t-complex polypeptide 1 [Homo sapiens] |

**TABLE SB-7: Other Receptor proteins detected**

| Accession | Mass | emPAI | Description |
| --- | --- | --- | --- |
| gi\|1477388 | 132282 | 0.07 | metabotropic glutamate receptor 1 alpha [Homo sapiens] |
| gi\|119581591 | 46372 | 0.2 | basigin (Ok blood group), isoform CRA_g [Homo sapiens] |
| gi\|187609338 | 20107 | 0.51 | Chain A, Crystal Structure Of The Extracellular Portion Of Hab18gCD147 |
| gi\|19743813 | 88357 | 0.47 | integrin beta-1 isoform 1A precursor [Homo sapiens] |
| gi\|124942 | 129214 | 0.14 | RecName: Full=Integrin alpha-2; AltName: Full=CD49 antigen-like family member B; AltName: Full=Collagen receptor; AltName: Full=Platelet membrane glycoprotein Ia; Short=GPIa; AltName: Full=VLA-2 subunit alpha; AltName: CD_antigen=CD49b; Flags: Precu |
| gi\|6424942 | 96019 | 0.05 | ALG-2 interacting protein 1 [Homo sapiens] |
| gi\|5729875 | 21658 | 0.21 | membrane-associated progesterone receptor component 1 isoform 1 [Homo sapiens] |
| gi\|4530577 | 65546 | 0.07 | LISCH protein [Homo sapiens] |
| gi\|21928311 | 35019 | 0.13 | seven transmembrane helix receptor [Homo sapiens] |
| gi\|62089376 | 70414 | 0.06 | complement component 1, q subcomponent, receptor 1 variant [Homo sapiens] |
| gi\|119611708 | 110644 | 0.04 | protein tyrosine phosphatase, receptor type, C, isoform CRA_c [Homo sapiens] |
| gi\|47077659 | 72805 | 0.06 | FLJ00268 protein [Homo sapiens] |
| gi\|1374813 | 23368 | 0.43 | SNAP-23 [Homo sapiens] |
| gi\|20072835 | 59945 | 0.07 | ACSL1 protein, partial [Homo sapiens] |
| gi\|5360115 | 89012 | 0.05 | NY-REN-45 antigen [Homo sapiens] |
| gi\|257743046 | 43424 | 0.1 | C-C chemokine receptor type 3 isoform 2 [Homo sapiens] |
| gi\|70913399 | 2021 | 4.89 | T cell receptor alpha variable 7 [Homo sapiens] |

**TABLE SB-8: Enzymes detected**

| Accession | Mass |  | emPAI | Description |
| --- | --- | --- | --- | --- |
| gi\|189095938 | 11754 |  | 0.42 | Chain A, Crystal Structure Of E60a Mutant Of Fkbp12 |
| gi\|530375065 | 51152 |  | 0.09 | PREDICTED: neutral cholesterol ester hydrolase 1 isoform X1 [Homo sapiens] |
| gi\|22209028 | 31786 |  | 0.3 | Thioredoxin-related transmembrane protein 1 [Homo sapiens] |
| gi\|4505977 | 32553 |  | 0.14 | lipid phosphate phosphohydrolase 2 isoform 1 [Homo sapiens] |
| gi\|530405604 | 56246 |  | 0.08 | PREDICTED: death-associated protein kinase 2 isoform X1 [Homo sapiens] |
| gi\|4505783 | 124805 |  | 0.03 | phosphorylase b kinase regulatory subunit beta isoform a [Homo sapiens] |
| gi\|1666423 | 161493 |  | 0.03 | FMI protein [Homo sapiens] |
| gi\|1486363 | 41988 |  | 0.11 | extracellular signal regulated kinase [Homo sapiens] |
| gi\|35360 | 65350 |  | 0.07 | PDC-E2 precursor (AA -54 to 561) [Homo sapiens] |
| gi\|516516 | 117320 |  | 0.04 | neuronal kinesin heavy chain [Homo sapiens] |
| gi\|181944 | 108185 |  | 0.04 | protein-tyrosine kinase [Homo sapiens] |
| gi\|16306598 | 153533 |  | 0.03 | von Willebrand factor-cleaving protease precursor [Homo sapiens] |
| gi\|5031697 | 143634 |  | 0.03 | probable phospholipid-transporting ATPase IC [Homo sapiens] |
| gi\|282847398 | 147344 |  | 0.03 | mitogen-activated protein kinase kinase kinase 15 [Homo sapiens] |
| gi\|134104091 | 31726 |  | 0.14 | Chain A, Crystal Structure Of Human Pyridoxal 5'-Phosphate Phosphatase |
| gi\|837261 | 88581 |  | 0.05 | ERK5 [Homo sapiens] |
| gi\|62897321 | 34328 |  | 0.13 | steroid dehydrogenase homolog [Homo sapiens] |
| gi\|3450828 | 33499 |  | 0.13 | retinal short-chain dehydrogenase/reductase retSDR1 [Homo sapiens] |
| gi\|124007195 | 73247 |  | 0.06 | RecName: Full=Heparan-alpha-glucosaminide N-acetyltransferase; AltName: Full=Transmembrane protein 76 [Homo sapiens] |
| gi\|13242866 | 31413 |  | 0.14 | type 3 iodothyronine deiodinase [Homo sapiens] |

**TABLE SB-9: Other membrane proteins detected**

| Accession | Mass | emPAI | | Description |
| --- | --- | --- | --- | --- |
| gi\|13129092 | 26194 | 0.38 | transmembrane protein 109 precursor [Homo sapiens] | |
| gi\|5821140 | 40308 | 0.37 | ASY [Homo sapiens] | |
| gi\|345198270 | 23750 | 0.69 | tumor protein D54 isoform j [Homo sapiens] | |
| gi\|337930 | 27386 | 0.84 | scar protein [Homo sapiens] | |
| gi\|23712 | 45220 | 0.32 | myoblast antigen 24.1D5 [Homo sapiens] | |
| gi\|1160963 | 83616 | 0.29 | transmembrane protein [Homo sapiens] | |
| gi\|662994 | 72707 | 0.06 | GPI-anchored protein p137 [Homo sapiens] | |
| gi\|5729718 | 46003 | 0.1 | trophoblast glycoprotein precursor [Homo sapiens] | |
| gi\|578830259 | 58187 | 0.08 | PREDICTED: brain-specific angiogenesis inhibitor 1-associated protein 2 isoform X11 [Homo sapiens] | |
| gi\|7767178 | 19180 | 0.24 | Chain A, Apolipoprotein E3 (Apoe3) Truncation Mutant 165 | |
| gi\|6912582 | 30361 | 0.15 | peflin [Homo sapiens] | |
| gi\|6002678 | 45242 | 0.1 | colon carcinoma related protein [Homo sapiens] | |
| gi\|19923969 | 25820 | 0.18 | coiled-coil domain-containing protein 124 [Homo sapiens] | |
| gi\|39725636 | 27260 | 0.17 | transmembrane emp24 domain-containing protein 9 precursor [Homo sapiens] | |
| gi\|19920317 | 65983 | 0.14 | cytoskeleton-associated protein 4 [Homo sapiens] | |
| gi\|1161384 | 384046 | 0.01 | BRCA2 [Homo sapiens] | |
| gi\|40789001 | 108477 | 0.04 | KIAA0964 protein [Homo sapiens] | |
| gi\|4240313 | 149996 | 0.03 | KIAA0912 protein [Homo sapiens] | |
| gi\|30089664 | 575806 | 0.01 | ABC A13 [Homo sapiens] | |
| gi\|27735113 | 50114 | 0.09 | EF-hand calcium-binding domain-containing protein 3 isoform b [Homo sapiens] | |
| gi\|28680 | 21594 | 0.21 | amphiglycan [Homo sapiens] | |
| gi\|235397 | 24680 | 0.18 | HMFG, partial [Homo sapiens] | |
| gi\|15012003 | 17359 | 0.27 | Family with sequence similarity 162, member A [Homo sapiens] | |
| gi\|1616918 | 46323 | 0.1 | membrane protein with histidine rich charge clusters [Homo sapiens] | |
| gi\|6331328 | 115103 | 0.04 | KIAA1280 protein [Homo sapiens] | |
| gi\|29648540 | 14892 | 0.32 | MLL5 [Homo sapiens] | |
| gi\|20521880 | 198894 | 0.02 | KIAA1305 protein [Homo sapiens] | |
| gi\|7706322 | 28051 | 0.16 | UPF0568 protein C14orf166 [Homo sapiens] | |
| gi\|471270576 | 23424 | 0.2 | Chain L, Crystal Structure Of Tnf-alpha In Complex With Infliximab Fab Fragment | |
| gi\|71891687 | 139575 | 0.03 | KIAA0931 protein [Homo sapiens] | |
| gi\|223462187 | 266458 | 0.02 | SPG11 protein [Homo sapiens] | |

**TABLE SC: Proteomics data of plasma membrane protein from POS**

**
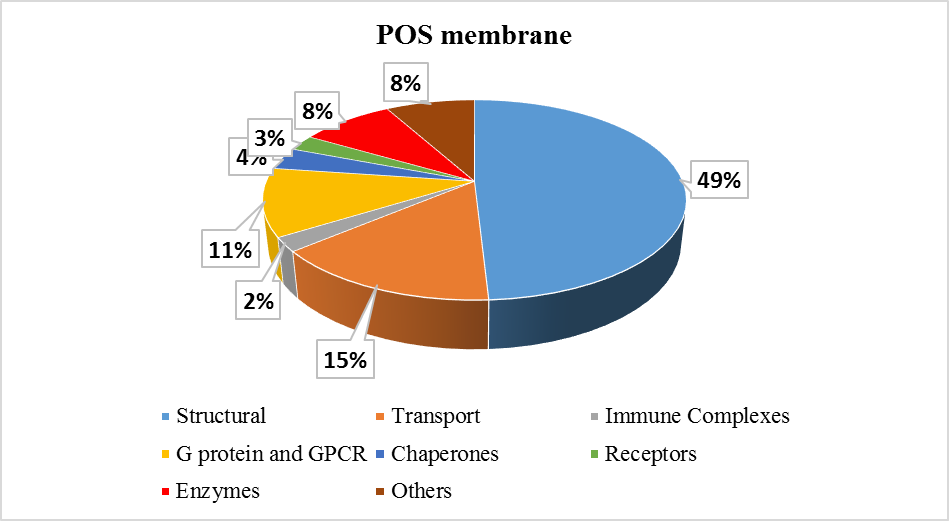
**

**TABLE SC-1. Overall proteomic profile**

|  | **emPAI** | **Percentage** |
| --- | --- | --- |
| Structural | 36.45 | 49.11 |
| Transport | 11.00 | 14.82 |
| Immune Complexes | 1.68 | 2.26 |
| G protein and GPCR | 8.16 | 10.99 |
| Chaperones | 2.68 | 3.61 |
| Receptors | 1.91 | 2.57 |
| Enzymes | 6.32 | 8.52 |
| Others | 6.02 | 8.11 |
| **Total** | **74.22** | **100** |

**TABLE SC-2 Structural Proteins Identified**

| **Accession** | **Mass** | **emPAI** | **Description** |
| --- | --- | --- | --- |
| **gi\|4501885** | 41710 | 6.01 | actin, cytoplasmic 1 [Homo sapiens] |
| **gi\|6755901** | 50104 | 1.56 | tubulin alpha-1A chain [Mus musculus] |
| **gi\|73997851** | 49990 | 0.54 | PREDICTED: tubulin alpha-8 chain [Canis lupus familiaris] |
| **gi\|61740600** | 57676 | 1.83 | keratin, type I cytoskeletal 10 [Canis lupus familiaris] |
| **gi\|545510666** | 54047 | 0.27 | PREDICTED: keratin, type I cytoskeletal 42 [Canis lupus familiaris] |
| **gi\|62122767** | 76308 | 0.12 | keratin, type I cytoskeletal 9 [Canis lupus familiaris] |
| **gi\|50950177** | 38630 | 4.26 | annexin A2 [Canis lupus familiaris] |
| **gi\|7106439** | 49639 | 2.65 | tubulin beta-5 chain [Mus musculus] |
| **gi\|5174735** | 49799 | 2.06 | tubulin beta-4B chain [Homo sapiens] |
| **gi\|345803407** | 49835 | 0.54 | PREDICTED: tubulin beta-6 chain isoform 3 [Canis lupus familiaris] |
| **gi\|50979272** | 63751 | 1.4 | keratin, type II cytoskeletal 1 [Canis lupus familiaris] |
| **gi\|345791904** | 62778 | 0.41 | PREDICTED: keratin, type II cytoskeletal 5 isoform X1 [Canis lupus familiaris] |
| **gi\|545545102** | 63592 | 0.5 | PREDICTED: keratin, type II cytoskeletal 2 epidermal isoform X1 [Canis lupus familiaris] |
| **gi\|359323093** | 54684 | 0.48 | PREDICTED: keratin, type II cytoskeletal 8 [Canis lupus familiaris] |
| **gi\|345791839** | 62832 | 0.23 | PREDICTED: keratin, type II cytoskeletal 6A isoformX2 [Canis lupus familiaris] |
| **gi\|545545370** | 63946 | 0.22 | PREDICTED: keratin, type II cytoskeletal 73 isoform X1 [Canis lupus familiaris] |
| **gi\|74008809** | 280450 | 0.26 | PREDICTED: filamin-A isoform 2 [Canis lupus familiaris] |
| **gi\|559098393** | 53565 | 3.22 | vimentin [Canis lupus familiaris] |
| **gi\|558695394** | 38602 | 1.42 | annexin A1 [Canis lupus familiaris] |
| **gi\|73953627** | 75842 | 0.87 | PREDICTED: annexin A6 isoformX2 [Canis lupus familiaris] |
| **gi\|160425231** | 226328 | 0.23 | myosin-9 [Canis lupus familiaris] |
| **gi\|345802274** | 223986 | 0.04 | PREDICTED: myosin-11 isoformX29 [Canis lupus familiaris] |
| **gi\|5031635** | 18491 | 0.98 | cofilin-1 [Homo sapiens] |
| **gi\|545558304** | 69724 | 0.54 | PREDICTED: moesin isoform X3 [Canis lupus familiaris] |
| **gi\|74005014** | 131810 | 0.14 | PREDICTED: unconventional myosin-Ib isoformX2 [Canis lupus familiaris] |
| **gi\|50978940** | 129321 | 0.14 | collagen alpha-2(I) chain precursor [Canis lupus familiaris] |
| **gi\|73947726** | 104337 | 0.09 | PREDICTED: alpha-actinin-4 isoformX6 [Canis lupus familiaris] |
| **gi\|74005014** | 131810 | 0.14 | PREDICTED: unconventional myosin-Ib isoformX2 [Canis lupus familiaris] |
| **gi\|73969959** | 65382 | 0.58 | PREDICTED: cytoskeleton-associated protein 4 [Canis lupus familiaris] |
| **gi\|545494454** | 47490 | 0.72 | PREDICTED: lactadherin [Canis lupus familiaris] |
| **gi\|55742853** | 35790 | 0.13 | annexin A4 [Canis lupus familiaris] |
| **gi\|57106334** | 48209 | 0.09 | PREDICTED: keratin, type I cytoskeletal 18 isoform 1 [Canis lupus familiaris] |
| **gi\|17986258** | 16919 | 0.28 | myosin light polypeptide 6 isoform 1 [Homo sapiens] |
| **gi\|359320831** | 269595 | 0.07 | PREDICTED: talin-1 isoform X1 [Canis lupus familiaris] |
| **gi\|157151714** | 342453 | 0.01 | collagen alpha-3(VI) chain precursor [Canis lupus familiaris] |
| **gi\|460417294** | 41977 | 0.11 | actin-related protein 3 isoform 2 [Homo sapiens] |
|  |  |  |  |
| **gi\|13937393** | 46861 | 0.1 | actin-like protein 6B [Mus musculus] |
| **gi\|545552032** | 114397 | 0.04 | PREDICTED: SWI/SNF-related matrix-associated actin-dependent regulator of chromatin subfamily A containing DEAD/H box 1 isoform X7 [Canis lupus familiaris] |
| **gi\|545530710** | 68641 | 0.13 | PREDICTED: LOW QUALITY PROTEIN: kinesin light chain 2 [Canis lupus familiaris] |
| **gi\|545507020** | 59610 | 0.07 | PREDICTED: cochlin [Canis lupus familiaris] |
| **gi\|73997258** | 106132 | 0.04 | PREDICTED: calsyntenin-3 isoform 1 [Canis lupus familiaris] |
| **gi\|545551547** | 78081 | 0.06 | PREDICTED: collagen alpha-2(VI) chain [Canis lupus familiaris] |
| **gi\|545517725** | 149690 | 0.03 | PREDICTED: collagen alpha-1(XV) chain isoform X1 [Canis lupus familiaris] |
| **gi\|545529126** | 126396 | 0.03 | PREDICTED: collagen alpha-1(I) chain [Canis lupus familiaris] |
| **gi\|545499057** | 41024 | 0.23 | PREDICTED: ankyrin repeat domain-containing protein 65 isoform X2 [Canis lupus familiaris] |
| **gi\|545519655** | 339256 | 0.03 | PREDICTED: collagen alpha-1(XII) chain isoform X2 [Canis lupus familiaris] |
| **gi\|545550190** | 52694 | 0.08 | PREDICTED: ankyrin repeat and death domain-containing protein 1A isoform X2 [Canis lupus familiaris] |
| **gi\|73966959** | 53672 | 0.08 | PREDICTED: vitronectin isoform 2 [Canis lupus familiaris] |
| **gi\|545521155** | 534043 | 0.04 | PREDICTED: plectin isoform X9 [Canis lupus familiaris] |
| **gi\|73961895** | 20485 | 0.23 | PREDICTED: myosin regulatory light polypeptide 9-like isoformX1 [Canis lupus familiaris] |
| **gi\|73986060** | 243902 | 0.02 | PREDICTED: unconventional myosin-IXb isoform 1 [Canis lupus familiaris] |
| **gi\|345778515** | 114475 | 0.04 | PREDICTED: collagen alpha-1(XIX) chain isoform X4 [Canis lupus familiaris] |
| **gi\|73953207** | 36599 | 0.12 | PREDICTED: annexin A8 isoformX1 [Canis lupus familiaris] |
| **gi\|545503404** | 174479 | 0.03 | PREDICTED: collagen alpha-1(XXIV) chain isoform X2 [Canis lupus familiaris] |
| **gi\|73994333** | 117807 | 0.04 | PREDICTED: FERM, RhoGEF and pleckstrin domain-containing protein 2 isoform X3 [Canis lupus familiaris] |
| **gi\|545531759** | 99866 | 0.04 | PREDICTED: protein inturned isoform X3 [Canis lupus familiaris] |
| **gi\|57095096** | 80886 | 0.05 | PREDICTED: lebercilin isoform X11 [Canis lupus familiaris] |
| **gi\|545519914** | 632508 | 0.01 | PREDICTED: midasin isoform X1 [Canis lupus familiaris] |
| **gi\|73981496** | 32964 | 0.14 | PREDICTED: F-actin-capping protein subunit alpha-1 isoform 2 [Canis lupus familiaris] |
| **gi\|359322210** | 111999 | 0.08 | PREDICTED: microtubule-associated protein 1S isoform X1 [Canis lupus familiaris] |
| **gi\|545492158** | 195634 | 0.02 | PREDICTED: LOW QUALITY PROTEIN: protocadherin gamma-A12 [Canis lupus familiaris] |
| **gi\|359322609** | 151053 | 0.03 | PREDICTED: nephrocystin-3 [Canis lupus familiaris] |
| **gi\|345789814** | 142795 | 0.03 | PREDICTED: collagen alpha-1(XX) chain [Canis lupus familiaris] |
| **gi\|545501026** | 56949 | 0.08 | PREDICTED: BAI1-associated protein 2-like 1 isoformX1 [Canis lupus familiaris] |
| **gi\|545533749** | 203686 | 0.02 | PREDICTED: laminin subunit beta-2 isoform X1 [Canis lupus familiaris] |
| **gi\|545539045** | 144440 | 0.03 | PREDICTED: cohesin subunit SA-1 [Canis lupus familiaris] |
| **gi\|545529816** | 113484 | 0.04 | PREDICTED: ankyrin repeat and BTB/POZ domain-containing protein 2 isoformX2 [Canis lupus familiaris] |
| **gi\|545541617** | 77260 | 0.06 | PREDICTED: cytoskeleton-associated protein 2 isoform X2 [Canis lupus familiaris] |
| **gi\|73949468** | 130850 | 0.03 | PREDICTED: protocadherin-12 [Canis lupus familiaris] |
| **gi\|545492234** | 473796 | 0.02 | PREDICTED: LOW QUALITY PROTEIN: basement membrane-specific heparan sulfate proteoglycan core protein [Canis lupus familiaris] |
| **gi\|73957734** | 84663 | 0.05 | PREDICTED: procollagen-lysine,2-oxoglutarate 5-dioxygenase 3 isoform 1 [Canis lupus familiaris] |
| **gi\|545529571** | 85651 | 0.05 | PREDICTED: CD44 antigen isoform X1 [Canis lupus familiaris] |
| **gi\|545489377** | 58668 | 0.16 | PREDICTED: collagen alpha-1(III) chain-like [Canis lupus familiaris] |
| **gi\|545530382** | 45606 | 0.1 | PREDICTED: lymphocyte-specific protein 1 isoform X1 [Canis lupus familiaris] |
| **gi\|73954163** | 480212 | 0.01 | PREDICTED: protocadherin Fat 2 [Canis lupus familiaris] |
| **gi\|545486940** | 78614 | 0.06 | PREDICTED: extracellular matrix protein 2 isoform X1 [Canis lupus familiaris] |
| **gi\|545549404** | 39867 | 0.11 | PREDICTED: ankyrin repeat domain-containing protein 63 [Canis lupus familiaris] |
| **gi\|165973990** | 222838 | 0.04 | myosin-7 [Canis lupus familiaris] |
| **gi\|545503105** | 61681 | 0.07 | PREDICTED: palmdelphin [Canis lupus familiaris] |
| **gi\|307938297** | 78906 | 0.06 | nephrocystin-1 [Canis lupus familiaris] |
| **gi\|95925865** | 87375 | 0.05 | pinin 1 [Canis lupus familiaris] |
| **gi\|545531227** | 579498 | 0.01 | PREDICTED: LOW QUALITY PROTEIN: neuroblast differentiation-associated protein AHNAK [Canis lupus familiaris] |
| **gi\|545551689** | 208083 | 0.02 | PREDICTED: protein Shroom3 isoform X1 [Canis lupus familiaris] |
| **gi\|545525131** | 310349 | 0.01 | PREDICTED: LOW QUALITY PROTEIN: protocadherin-23-like [Canis lupus familiaris] |
| **gi\|345796528** | 46416 | 0.1 | PREDICTED: neuroserpin isoform X3 [Canis lupus familiaris] |
| **gi\|4757952** | 21245 | 0.22 | cell division control protein 42 homolog isoform 1 precursor [Homo sapiens] |
| **gi\|304376314** | 240303 | 0.02 | tenascin precursor [Canis lupus familiaris] |
| **gi\|345801801** | 172246 | 0.03 | PREDICTED: leucine-rich repeat-containing protein 7 isoform X2 [Canis lupus familiaris] |
| **gi\|74000367** | 271428 | 0.02 | PREDICTED: talin-2 isoformX1 [Canis lupus familiaris] |

**TABLE SC-3: Transport Proteins Identified**

| **Accession** | **Mass** | **emPAI** | | **Description** |
| --- | --- | --- | --- | --- |
| **gi\|545528359** | 112738 | 0.64 | PREDICTED: sodium/potassium-transporting ATPase subunit alpha-1 isoform X1 [Canis lupus familiaris] | |
| **gi\|345797872** | 114359 | 0.08 | PREDICTED: sodium/potassium-transporting ATPase subunit alpha-4 isoform X2 [Canis lupus familiaris] | |
| **gi\|73953093** | 31559 | 0.52 | PREDICTED: voltage-dependent anion-selective channel protein 2 isoform 2 [Canis lupus familiaris] | |
| **gi\|345807347** | 134128 | 0.03 | PREDICTED: plasma membrane calcium-transporting ATPase 3 isoform X36 [Canis lupus familiaris] | |
| **gi\|126723018** | 30722 | 2.38 | voltage-dependent anion-selective channel protein 1 [Oryctolagus cuniculus] | |
| **gi\|74008194** | 32996 | 1.47 | PREDICTED: ADP/ATP translocase 2 isoform 2 [Canis lupus familiaris] | |
| **gi\|545559996** | 32512 | 1.5 | PREDICTED: ADP/ATP translocase 3 [Canis lupus familiaris] | |
| **gi\|73965153** | 83507 | 0.05 | PREDICTED: vesicle-fusing ATPase isoformX1 [Canis lupus familiaris] | |
| **gi\|325301273** | 18932 | 0.95 | translocon-associated protein subunit delta precursor [Canis lupus familiaris] | |
| **gi\|545560056** | 17447 | 0.27 | PREDICTED: proteolipid protein 2 [Canis lupus familiaris] | |
| **gi\|73963665** | 24765 | 0.19 | PREDICTED: transmembrane emp24 domain-containing protein 10 isoform 1 [Canis lupus familiaris] | |
| **gi\|6002950** | 66639 | 0.07 | triadin isoform 3 [Canis lupus familiaris] | |
| **gi\|545504506** | 278817 | 0.02 | PREDICTED: voltage-dependent R-type calcium channel subunit alpha-1E isoformX3 [Canis lupus familiaris] | |
| **gi\|73959904** | 61702 | 0.07 | PREDICTED: potassium voltage-gated channel subfamily A member 10 [Canis lupus familiaris] | |
| **gi\|545489180** | 93799 | 0.05 | PREDICTED: transmembrane channel-like 1 [Canis lupus familiaris] | |
| **gi\|30410788** | 39650 | 0.11 | tumor suppressor candidate 3 isoform a precursor [Homo sapiens] | |
| **gi\|73967844** | 94035 | 0.05 | PREDICTED: leucine-rich repeat-containing protein 8A isoformX1 [Canis lupus familiaris] | |
| **gi\|545516959** | 73609 | 0.12 | PREDICTED: polycystic kidney disease 2-like 2 protein isoform X2 [Canis lupus familiaris] | |
| **gi\|4557469** | 104486 | 0.04 | AP-2 complex subunit beta isoform b [Homo sapiens] | |
| **gi\|345781116** | 111392 | 0.04 | PREDICTED: anoctamin-4 isoformX1 [Canis lupus familiaris] | |
| **gi\|73998421** | 94181 | 0.05 | PREDICTED: metal transporter CNNM2 isoformX4 [Canis lupus familiaris] | |
| **gi\|545547011** | 22720 | 0.2 | PREDICTED: V-type proton ATPase subunit E 1 isoform X2 [Canis lupus familiaris] | |
| **gi\|22759019** | 141456 | 0.03 | multidrug resistance p-glycoprotein [Canis lupus familiaris] | |
| **gi\|73979747** | 43639 | 0.1 | PREDICTED: V-type proton ATPase subunit C 2 isoformX1 [Canis lupus familiaris] | |
| **gi\|545531548** | 121210 | 0.04 | PREDICTED: anoctamin-1 [Canis lupus familiaris] | |
| **gi\|345801671** | 26040 | 0.18 | PREDICTED: transmembrane emp24 domain-containing protein 5 isoform 2 [Canis lupus familiaris] | |
| **gi\|359321467** | 136023 | 0.03 | PREDICTED: anion exchange protein 2 isoform 1 [Canis lupus familiaris] | |
| **gi\|345782667** | 11210 | 0.45 | PREDICTED: protein S100-A10 isoform 1 [Canis lupus familiaris] | |
| **gi\|73997059** | 47923 | 0.09 | PREDICTED: ATP-sensitive inward rectifier potassium channel 8 isoformX1 [Canis lupus familiaris] | |
| **gi\|545537221** | 52108 | 0.09 | PREDICTED: synaptotagmin-9 [Canis lupus familiaris] | |
| **gi\|6753074** | 49623 | 0.09 | AP-2 complex subunit mu [Mus musculus] | |
| **gi\|345800844** | 45731 | 0.1 | PREDICTED: vesicle amine transport protein 1 homolog (T. californica)-like [Canis lupus familiaris] | |
| **gi\|545491848** | 44646 | 0.1 | PREDICTED: NIPA-like protein 3 isoform X1 [Canis lupus familiaris] | |
| **gi\|73983332** | 61656 | 0.07 | PREDICTED: solute carrier family 22 member 9 isoformX1 [Canis lupus familiaris] | |
| **gi\|73953291** | 36136 | 0.13 | PREDICTED: graves disease carrier protein isoform X2 [Canis lupus familiaris] | |
| **gi\|559098415** | 90911 | 0.05 | sodium/hydrogen exchanger 1 [Canis lupus familiaris] | |
| **gi\|270288810** | 107786 | 0.04 | anoctamin-5 precursor [Canis lupus familiaris] | |
| **gi\|6755588** | 23300 | 0.2 | synaptosomal-associated protein 25 isoform a [Mus musculus] | |
| **gi\|345795474** | 141471 | 0.03 | PREDICTED: trafficking protein particle complex subunit 10 isoform X2 [Canis lupus familiaris] | |
| **gi\|545494271** | 78141 | 0.06 | PREDICTED: sodium-dependent phosphate transport protein 2B isoform X2 [Canis lupus familiaris] | |
| **gi\|55741729** | 220559 | 0.02 | sodium channel protein type 10 subunit alpha [Canis lupus familiaris] | |
| **gi\|73979590** | 128727 | 0.03 | PREDICTED: trafficking protein particle complex subunit 11 [Canis lupus familiaris] | |
| **gi\|73979536** | 31206 | 0.15 | PREDICTED: neuronal membrane glycoprotein M6-a isoform 1 [Canis lupus familiaris] | |
| **gi\|545510114** | 184849 | 0.02 | PREDICTED: ATP-binding cassette sub-family A member 6 [Canis lupus familiaris] | |

**TABLE SC-4: Immune Complex Proteins Identified**

| **Accession** | **Mass** | **emPAI** |  | **Description** |
| --- | --- | --- | --- | --- |
| **gi\|122135** | 40437 | 0.11 |  | RecName: Full=DLA class I histocompatibility antigen, A9/A9 alpha chain; Flags: Precursor |
| **gi\|208342202** | 15306 | 0.32 |  | immunoglobulin heavy chain variable region, partial [Canis lupus familiaris] |
| **gi\|545493610** | 148085 | 0.03 |  | PREDICTED: pro-interleukin-16 isoform X2 [Canis lupus familiaris] |
| **gi\|350543372** | 10740 | 0.47 |  | MHC class II antigen [Canis lupus familiaris] |
| **gi\|345783307** | 72501 | 0.06 |  | PREDICTED: T-cell differentiation antigen CD6 isoform X4 [Canis lupus familiaris] |
| **gi\|545509559** | 20851 | 0.22 |  | PREDICTED: CMRF35-like molecule 7-like [Canis lupus familiaris] |
| **gi\|74007037** | 64149 | 0.07 |  | PREDICTED: melanoma-associated antigen D2 isoformX2 [Canis lupus familiaris] |
| **gi\|6572519** | 32245 | 0.14 |  | truncated B7-2 protein [Canis lupus familiaris] |
| **gi\|57112869** | 28042 | 0.16 |  | PREDICTED: B-cell receptor-associated protein 31 isoform X3 [Canis lupus familiaris] |
| **gi\|545514193** | 87364 | 0.05 |  | PREDICTED: interleukin-1 receptor-associated kinase 3 [Canis lupus familiaris] |
| **gi\|70794790** | 96206 | 0.05 |  | interleukin-12 receptor subunit beta-2 precursor [Canis lupus familiaris] |

**TABLE SC-5: GPCRs and G proteins identified**

| **Accession** | **Mass** | **emPAI** | | **Description** |
| --- | --- | --- | --- | --- |
| **gi\|5174447** | 35055 | 1.08 | guanine nucleotide-binding protein subunit beta-2-like 1 [Homo sapiens] | |
| **gi\|50979222** | 40520 | 0.37 | guanine nucleotide-binding protein G(i) subunit alpha-2 [Canis lupus familiaris] | |
| **gi\|545506467** | 50860 | 0.18 | PREDICTED: LOW QUALITY PROTEIN: guanine nucleotide-binding protein G(olf) subunit alpha [Canis lupus familiaris] | |
| **gi\|55742672** | 37120 | 0.12 | guanine nucleotide-binding protein G(I)/G(S)/G(T) subunit beta-3 [Canis lupus familiaris] | |
| **gi\|345777861** | 36294 | 0.12 | PREDICTED: olfactory receptor 13F1-like [Canis lupus familiaris] | |
| **gi\|545510010** | 33160 | 0.14 | PREDICTED: guanine nucleotide-binding protein subunit alpha-13 isoform X1 [Canis lupus familiaris] | |
| **gi\|74009012** | 328348 | 0.01 | PREDICTED: probable G-protein coupled receptor 112 [Canis lupus familiaris] | |
| **gi\|545523201** | 34864 | 0.13 | PREDICTED: olfactory receptor 2T27-like [Canis lupus familiaris] | |
| **gi\|73985077** | 21585 | 0.48 | PREDICTED: PRA1 family protein 3 [Canis lupus familiaris] | |
| **gi\|72535184** | 20414 | 0.23 | ADP-ribosylation factor-like protein 1 [Sus scrofa] | |
| **gi\|545509639** | 71038 | 0.06 | PREDICTED: septin-9 [Canis lupus familiaris] | |
| **gi\|77736007** | 20515 | 0.23 | ADP-ribosylation factor 4 [Bos taurus] | |
| **gi\|545545958** | 144009 | 0.03 | PREDICTED: DENN domain-containing protein 5B [Canis lupus familiaris] | |
| **gi\|73985028** | 124393 | 0.04 | PREDICTED: SLIT-ROBO Rho GTPase-activating protein 3 isoformX2 [Canis lupus familiaris] | |
| **gi\|73966150** | 257794 | 0.02 | PREDICTED: probable G-protein coupled receptor 179 isoform X3 [Canis lupus familiaris] | |
| **gi\|511914243** | 26132 | 0.18 | PREDICTED: GTPase KRas isoform X1 [Mustela putorius furo] | |
| **gi\|6981476** | 20467 | 0.23 | GTP-binding protein Rheb precursor [Rattus norvegicus] | |
| **gi\|345793806** | 46359 | 0.1 | PREDICTED: probable G-protein coupled receptor 151 [Canis lupus familiaris] | |
| **gi\|545539728** | 215508 | 0.02 | PREDICTED: ral GTPase-activating protein subunit alpha-2 isoform X1 [Canis lupus familiaris] | |
| **gi\|345807071** | 38241 | 0.12 | PREDICTED: probable G-protein coupled receptor 82 [Canis lupus familiaris] | |
| **gi\|359320566** | 51114 | 0.09 | PREDICTED: rab-3A-interacting protein isoform X6 [Canis lupus familiaris] | |
| **gi\|73983774** | 58829 | 0.08 | PREDICTED: atlastin-3 isoform X4 [Canis lupus familiaris] | |
| **gi\|345788827** | 38635 | 0.12 | PREDICTED: growth hormone-regulated TBC protein 1 [Canis lupus familiaris] | |
| **gi\|545522667** | 101092 | 0.04 | PREDICTED: rap guanine nucleotide exchange factor 5 isoform X2 [Canis lupus familiaris] | |
| **gi\|545529658** | 59449 | 0.07 | PREDICTED: synembryn-A [Canis lupus familiaris] | |
| **gi\|545541279** | 45440 | 0.1 | PREDICTED: histamine H3 receptor, partial [Canis lupus familiaris] | |
| **gi\|545497487** | 129263 | 0.03 | PREDICTED: rho GTPase-activating protein 20 isoform X1 [Canis lupus familiaris] | |
| **gi\|545516132** | 139898 | 0.03 | PREDICTED: TBC1 domain family member 9B isoform X1 [Canis lupus familiaris] | |
| **gi\|545555224** | 101745 | 0.04 | PREDICTED: rap guanine nucleotide exchange factor 4 isoform X1 [Canis lupus familiaris] | |
| **gi\|545548334** | 71664 | 0.06 | PREDICTED: rho GTPase-activating protein 22 [Canis lupus familiaris] | |
| **gi\|194339213** | 9761 | 0.53 | dynanmin 1 protein [Canis lupus familiaris] | |
| **gi\|345793422** | 139573 | 0.03 | PREDICTED: LOW QUALITY PROTEIN: probable G-protein coupled receptor 158 [Canis lupus familiaris] | |
| **gi\|73971093** | 54255 | 0.08 | PREDICTED: protein C9orf72 isoformX2 [Canis lupus familiaris] | |
| **gi\|545516757** | 51263 | 0.09 | PREDICTED: septin-8 isoform X1 [Canis lupus familiaris] | |
| **gi\|545543197** | 53123 | 0.08 | PREDICTED: septin-2 isoform X1 [Canis lupus familiaris] | |
| **gi\|8926588** | 48722 | 0.09 | endothelin B receptor [Canis lupus familiaris] | |
|  |  |  |  | |
| **gi\|131804** | 22555 | 0.76 | RecName: Full=Ras-related protein Rab-10 [Canis lupus familiaris] | |
| **gi\|13569962** | 22157 | 0.77 | ras-related protein Rab-1B [Homo sapiens] | |
| **gi\|50979156** | 23505 | 0.2 | ras-related protein Rab-7a [Canis lupus familiaris] | |
| **gi\|50979062** | 23441 | 0.2 | ras-related protein Rab-5C [Canis lupus familiaris] | |
| **gi\|4506413** | 20974 | 0.22 | ras-related protein Rap-1A precursor [Homo sapiens] | |
| **gi\|4758984** | 24378 | 0.19 | ras-related protein Rab-11A isoform 1 [Homo sapiens] | |
| **gi\|345785574** | 103197 | 0.04 | PREDICTED: ras-interacting protein 1 isoform 1 [Canis lupus familiaris] | |
| **gi\|545548447** | 143013 | 0.03 | PREDICTED: LOW QUALITY PROTEIN: protein very KIND [Canis lupus familiaris] | |
| **gi\|545525085** | 55435 | 0.08 | PREDICTED: ras association domain-containing protein 9 [Canis lupus familiaris] | |
| **gi\|9845511** | 21436 | 0.22 | ras-related C3 botulinum toxin substrate 1 isoform Rac1 [Homo sapiens] | |

**TABLE SC-6: Chaperone proteins identified**

| **Accession** | **Mass** | **emPAI** | **Description** |
| --- | --- | --- | --- |
| **gi\|545508859** | 72881 | 0.42 | PREDICTED: heat shock protein HSP 90-alpha, partial [Canis lupus familiaris] |
| **gi\|545500762** | 58230 | 0.25 | PREDICTED: T-complex protein 1 subunit zeta [Canis lupus familiaris] |
| **gi\|359323746** | 59559 | 0.24 | PREDICTED: LOW QUALITY PROTEIN: T-complex protein 1 subunit epsilon isoform 1 [Canis lupus familiaris] |
| **gi\|159794954** | 76432 | 0.66 | Chain A, Structure Of Full Length Grp94 With Amp-Pnp Bound |
| **gi\|545550950** | 77756 | 0.06 | PREDICTED: T-complex protein 1 subunit theta isoform X2 [Canis lupus familiaris] |
| **gi\|345802573** | 60589 | 0.07 | PREDICTED: T-complex protein 1 subunit gamma isoform 1 [Canis lupus familiaris] |
| **gi\|73980527** | 59383 | 0.07 | PREDICTED: T-complex protein 1 subunit eta isoform 2 [Canis lupus familiaris] |
| **gi\|57032236** | 60216 | 0.07 | PREDICTED: T-complex protein 1 subunit alpha isoformX1 [Canis lupus familiaris] |
| **gi\|73968673** | 57395 | 0.16 | PREDICTED: T-complex protein 1 subunit beta isoformX1 [Canis lupus familiaris] |

**ST- Receptors identified**

| **Accession** | **Mass** | **emPAI** | | **Description** |
| --- | --- | --- | --- | --- |
| **gi\|3641357** | 11736 | 0.42 | interferon gamma precursor [Canis lupus familiaris] | |
| **gi\|545534382** | 88794 | 0.05 | PREDICTED: EGF-like module-containing mucin-like hormone receptor-like 2-like isoform X1 [Canis lupus familiaris] | |
| **gi\|73970497** | 114071 | 0.04 | PREDICTED: semaphorin-6A isoformX2 [Canis lupus familiaris] | |
| **gi\|73998651** | 92273 | 0.05 | PREDICTED: semaphorin-4G isoform X4 [Canis lupus familiaris] | |
| **gi\|545536637** | 36244 | 0.12 | PREDICTED: LOW QUALITY PROTEIN: gastrin/cholecystokinin type B receptor, partial [Canis lupus familiaris] | |
| **gi\|73993667** | 53592 | 0.08 | PREDICTED: scavenger receptor class A member 5 isoformX1 [Canis lupus familiaris] | |
| **gi\|545518755** | 96015 | 0.05 | PREDICTED: metabotropic glutamate receptor 4 [Canis lupus familiaris] | |
| **gi\|50979238** | 39187 | 0.12 | C5a anaphylatoxin chemotactic receptor 1 [Canis lupus familiaris] | |
| **gi\|545552715** | 125167 | 0.07 | PREDICTED: ephrin type-A receptor 3 [Canis lupus familiaris] | |
| **gi\|73962317** | 227388 | 0.02 | PREDICTED: thyroid receptor-interacting protein 11 isoform X2 [Canis lupus familiaris] | |
| **gi\|359322039** | 312091 | 0.01 | PREDICTED: inositol 1,4,5-trisphosphate receptor type 1 isoform X1 [Canis lupus familiaris] | |
| **gi\|73966275** | 63426 | 0.07 | PREDICTED: insulin-like growth factor 2 mRNA binding protein 1 isoformX2 [Canis lupus familiaris] | |
| **gi\|545543633** | 51208 | 0.09 | PREDICTED: LOW QUALITY PROTEIN: hydroxycarboxylic acid receptor 3 [Canis lupus familiaris] | |
| **gi\|545517466** | 222263 | 0.02 | PREDICTED: protein unc-13 homolog B isoform X2 [Canis lupus familiaris] | |
| **gi\|545548378** | 118345 | 0.04 | PREDICTED: VPS10 domain-containing receptor SorCS1 [Canis lupus familiaris] | |
| **gi\|545522902** | 50679 | 0.09 | PREDICTED: corticotropin-releasing factor receptor 2 isoform X3 [Canis lupus familiaris] | |
| **gi\|359318678** | 564218 | 0.01 | PREDICTED: ryanodine receptor 1 isoform 1 [Canis lupus familiaris] | |
| **gi\|359319428** | 107139 | 0.04 | PREDICTED: glutamate receptor ionotropic, kainate 4 [Canis lupus familiaris] | |
| **gi\|73959181** | 165177 | 0.03 | PREDICTED: glutamate receptor ionotropic, NMDA 2A isoform X3 [Canis lupus familiaris] | |
| **gi\|545506274** | 81668 | 0.11 | PREDICTED: collectin-12 [Canis lupus familiaris] | |
| **gi\|545555520** | 130753 | 0.03 | PREDICTED: sperm-specific antigen 2, partial [Canis lupus familiaris] | |
| **gi\|73987612** | 29284 | 0.16 | PREDICTED: basigin [Canis lupus familiaris] | |
| **gi\|345796108** | 87112 | 0.1 | PREDICTED: integrin beta-5 [Canis lupus familiaris] | |
| **gi\|545555528** | 118416 | 0.04 | PREDICTED: integrin alpha-V isoform 2 [Canis lupus familiaris] | |

**ST- 3-8 : Enzymes identified**

| **Accession** | **Mass** | **emPAI** | **Description** |
| --- | --- | --- | --- |
| **gi\|73958481** | 39478 | 1.65 | PREDICTED: fructose-bisphosphate aldolase A isoformX2 [Canis lupus familiaris] |
| **gi\|73947982** | 62798 | 0.07 | PREDICTED: glucose-6-phosphate isomerase isoformX1 [Canis lupus familiaris] |
| **gi\|308082020** | 26698 | 0.17 | triosephosphate isomerase [Canis lupus familiaris] |
| **gi\|345800677** | 48956 | 0.19 | PREDICTED: LOW QUALITY PROTEIN: alpha-enolase isoform 1 [Canis lupus familiaris] |
| **gi\|359321459** | 72351 | 0.06 | PREDICTED: protein disulfide-isomerase A4 isoform 3 [Canis lupus familiaris] |
| **gi\|73980965** | 92301 | 0.05 | PREDICTED: mannosyl-oligosaccharide glucosidase [Canis lupus familiaris] |
| **gi\|71043798** | 44292 | 0.21 | cathepsin D precursor [Canis lupus familiaris] |
| **gi\|73965482** | 56737 | 0.08 | PREDICTED: glycylpeptide N-tetradecanoyltransferase 1 isoformX1 [Canis lupus familiaris] |
| **gi\|545505244** | 76224 | 0.06 | PREDICTED: calpain-2 catalytic subunit [Canis lupus familiaris] |
| **gi\|545529547** | 134832 | 0.03 | PREDICTED: membrane-associated guanylate kinase, WW and PDZ domain-containing protein 2 [Canis lupus familiaris] |
| **gi\|359319350** | 34044 | 0.28 | PREDICTED: cyclin-dependent kinase 1 isoformX1 [Canis lupus familiaris] |
| **gi\|11345462** | 20301 | 0.23 | signal peptidase complex subunit 3 [Homo sapiens] |
| **gi\|73973859** | 150034 | 0.06 | PREDICTED: inhibitor of Bruton tyrosine kinase isoform X5 [Canis lupus familiaris] |
| **gi\|5002350** | 42651 | 0.11 | alkaline phosphatase [Canis lupus familiaris] |
| **gi\|73949883** | 123762 | 0.07 | PREDICTED: phosphorylase b kinase regulatory subunit beta isoformX2 [Canis lupus familiaris] |
| **gi\|545552304** | 61205 | 0.07 | PREDICTED: calcium/calmodulin-dependent protein kinase type II subunit delta isoform X2 [Canis lupus familiaris] |
| **gi\|359323733** | 239523 | 0.02 | PREDICTED: basic helix-loop-helix domain-containing protein KIAA2018 homolog isoform X1 [Canis lupus familiaris] |
| **gi\|545513543** | 77724 | 0.06 | PREDICTED: sulfhydryl oxidase 2 [Canis lupus familiaris] |
| **gi\|74007716** | 136827 | 0.03 | PREDICTED: phosphorylase b kinase regulatory subunit alpha, skeletal muscle isoform isoformX5 [Canis lupus familiaris] |
| **gi\|73975797** | 40015 | 0.11 | PREDICTED: serum paraoxonase/arylesterase 1 isoform 2 [Canis lupus familiaris] |
| **gi\|545486104** | 155818 | 0.03 | PREDICTED: PH domain leucine-rich repeat-containing protein phosphatase 1 [Canis lupus familiaris] |
| **gi\|545507539** | 169948 | 0.03 | PREDICTED: leucine-rich repeat-containing protein 9-like isoform X1 [Canis lupus familiaris] |
| **gi\|345780013** | 101930 | 0.04 | PREDICTED: staphylococcal nuclease domain-containing protein 1 [Canis lupus familiaris] |
| **gi\|73950291** | 573081 | 0.02 | PREDICTED: E3 ubiquitin-protein ligase UBR4 isoformX2 [Canis lupus familiaris] |
| **gi\|545508966** | 50665 | 0.09 | PREDICTED: casein kinase I isoform delta isoform X1 [Canis lupus familiaris] |
| **gi\|545559938** | 63644 | 0.07 | PREDICTED: glucose-6-phosphate 1-dehydrogenase isoform X1 [Canis lupus familiaris] |
| **gi\|74005468** | 104700 | 0.04 | PREDICTED: GPI inositol-deacylase isoform X2 [Canis lupus familiaris] |
| **gi\|73979309** | 29068 | 0.16 | PREDICTED: phosphatidate phosphatase PPAPDC1B isoform X2 [Canis lupus familiaris] |
| **gi\|345777094** | 88480 | 0.05 | PREDICTED: transmembrane protease serine 6 isoform 2 [Canis lupus familiaris] |
| **gi\|345782357** | 87215 | 0.05 | PREDICTED: vitamin K-dependent gamma-carboxylase isoform X2 [Canis lupus familiaris] |
| **gi\|545492172** | 215322 | 0.02 | PREDICTED: microtubule-associated serine/threonine-protein kinase 4 [Canis lupus familiaris] |
| **gi\|545486178** | 181384 | 0.02 | PREDICTED: mitogen-activated protein kinase kinase kinase 4 [Canis lupus familiaris] |
| **gi\|345782971** | 16804 | 0.29 | PREDICTED: oligosaccharyltransferase complex subunit OSTC-like [Canis lupus familiaris] |
| **gi\|312283574** | 45486 | 0.1 | hydroxysteroid dehydrogenase-like protein 2 [Canis lupus familiaris] |
| **gi\|73986266** | 36011 | 0.13 | PREDICTED: very-long-chain enoyl-CoA reductase isoform 2 [Canis lupus familiaris] |
| **gi\|359322989** | 107203 | 0.04 | PREDICTED: serine/threonine-protein kinase ULK1 isoform X2 [Canis lupus familiaris] |
| **gi\|545541538** | 29373 | 0.16 | PREDICTED: cathepsin Z, partial [Canis lupus familiaris] |
| **gi\|73976741** | 51421 | 0.09 | PREDICTED: adenylyl cyclase-associated protein 1 isoformX2 [Canis lupus familiaris] |
| **gi\|545550634** | 60860 | 0.07 | PREDICTED: carbohydrate sulfotransferase 14 [Canis lupus familiaris] |
| **gi\|73969530** | 83522 | 0.05 | PREDICTED: protein kinase C epsilon type isoformX1 [Canis lupus familiaris] |
| **gi\|545528333** | 523742 | 0.01 | PREDICTED: baculoviral IAP repeat-containing protein 6 [Canis lupus familiaris] |
| **gi\|545531571** | 84382 | 0.05 | PREDICTED: N-acetylated alpha-linked acidic dipeptidase-like 1 [Canis lupus familiaris] |
| **gi\|545535937** | 45964 | 0.1 | PREDICTED: endonuclease domain-containing 1 protein, partial [Canis lupus familiaris] |
| **gi\|545511447** | 81119 | 0.05 | PREDICTED: myeloperoxidase [Canis lupus familiaris] |
| **gi\|74005739** | 132463 | 0.03 | PREDICTED: tubulin polyglutamylase TTLL4 isoform X3 [Canis lupus familiaris] |
| **gi\|545532775** | 97866 | 0.04 | PREDICTED: LOW QUALITY PROTEIN: tubulin monoglycylase TTLL3 [Canis lupus familiaris] |
| **gi\|73990288** | 42108 | 0.11 | PREDICTED: caspase-14-like [Canis lupus familiaris] |
| **gi\|399317** | 130240 | 0.03 | RecName: Full=Adenylate cyclase type 6; AltName: Full=ATP pyrophosphate-lyase 6; AltName: Full=Adenylate cyclase type VI; AltName: Full=Adenylyl cyclase 6; AltName: Full=Ca(2+)-inhibitable adenylyl cyclase |
| **gi\|545512439** | 160241 | 0.03 | PREDICTED: patatin-like phospholipase domain containing 7 isoformX1 [Canis lupus familiaris] |
| **gi\|545554163** | 127509 | 0.03 | PREDICTED: phospholipase D1 [Canis lupus familiaris] |
| **gi\|74006997** | 27138 | 0.37 | PREDICTED: 3-hydroxyacyl-CoA dehydrogenase type-2 isoform 1 [Canis lupus familiaris] |
| **gi\|558515998** | 88111 | 0.05 | carnitine O-palmitoyltransferase 1, liver isoform [Canis lupus familiaris] |
| **gi\|73983265** | 138671 | 0.03 | PREDICTED: 1-phosphatidylinositol 4,5-bisphosphate phosphodiesterase beta-3 isoformX2 [Canis lupus familiaris] |
| **gi\|212276080** | 258715 | 0.02 | 1-phosphatidylinositol-4,5-bisphosphate phosphodiesterase epsilon-1 [Canis lupus familiaris] |
| **gi\|545510617** | 49679 | 0.09 | PREDICTED: 2',3'-cyclic-nucleotide 3'-phosphodiesterase isoform X1 [Canis lupus familiaris] |
| **gi\|545529305** | 88247 | 0.05 | PREDICTED: calcium-independent phospholipase A2-gamma isoform X2 [Canis lupus familiaris] |
| **gi\|359320296** | 61456 | 0.07 | PREDICTED: GPI transamidase component PIG-S [Canis lupus familiaris] |

**Table SC-9: Other Proteins Identified**

| **Accession** | **Mass** | **emPAI** | **Description** |
| --- | --- | --- | --- |
| **gi\|345806081** | 72238 | 1.59 | PREDICTED: 78 kDa glucose-regulated protein isoform 5 [Canis lupus familiaris] |
| **gi\|73971240** | 38546 | 0.74 | PREDICTED: stomatin (EPB72)-like 2 isoform 1 [Canis lupus familiaris] |
| **gi\|345800374** | 42267 | 0.22 | PREDICTED: plasminogen activator inhibitor 1 RNA-binding protein isoform 1 [Canis lupus familiaris] |
| **gi\|335286672** | 22363 | 0.46 | PREDICTED: transgelin-2 isoformX1 [Sus scrofa] |
| **gi\|73968397** | 20542 | 0.23 | PREDICTED: protein canopy homolog 2 isoformX1 [Canis lupus familiaris] |
| **gi\|545543609** | 46653 | 0.1 | PREDICTED: LOW QUALITY PROTEIN: ADP-ribosylation-like factor 6 interacting protein 4 [Canis lupus familiaris] |
| **gi\|73994424** | 154240 | 0.03 | PREDICTED: protein strawberry notch homolog 1 isoformX2 [Canis lupus familiaris] |
| **gi\|134031952** | 60903 | 0.07 | suppressor of tumorigenicity 7 protein isoform 1 [Mus musculus] |
| **gi\|50979076** | 31956 | 0.14 | translocon-associated protein subunit alpha precursor [Canis lupus familiaris] |
| **gi\|73945930** | 58023 | 0.25 | PREDICTED: protein ERGIC-53 isoform X2 [Canis lupus familiaris] |
| **gi\|73989504** | 44247 | 0.34 | PREDICTED: lysosome-associated membrane glycoprotein 1 isoformX1 [Canis lupus familiaris] |
| **gi\|57096757** | 103880 | 0.04 | PREDICTED: transmembrane and TPR repeat-containing protein 3 isoform X2 [Canis lupus familiaris] |
| **gi\|345793179** | 35777 | 0.13 | PREDICTED: regulator of microtubule dynamics protein 1 isoform X3 [Canis lupus familiaris] |
| **gi\|545511167** | 43137 | 0.1 | PREDICTED: alpha-sarcoglycan isoform X1 [Canis lupus familiaris] |
| **gi\|545536806** | 98933 | 0.04 | PREDICTED: protein MRVI1 isoform X1 [Canis lupus familiaris] |
| **gi\|545492524** | 75786 | 0.06 | PREDICTED: erythrocyte membrane protein band 4.1 like 4A isoform X1 [Canis lupus familiaris] |
| **gi\|545526766** | 385163 | 0.01 | PREDICTED: CUB and sushi domain-containing protein 1 [Canis lupus familiaris] |
| **gi\|73996813** | 73879 | 0.06 | PREDICTED: dendrin isoform X4 [Canis lupus familiaris] |
| **gi\|345791006** | 137207 | 0.03 | PREDICTED: LOW QUALITY PROTEIN: probable tumor suppressor protein MN1 [Canis lupus familiaris] |
| **gi\|359319183** | 44586 | 0.1 | PREDICTED: arrestin domain-containing protein 4 [Canis lupus familiaris] |
| **gi\|345780090** | 451207 | 0.01 | PREDICTED: A-kinase anchor protein 9 isoform X3 [Canis lupus familiaris] |
| **gi\|545549726** | 278230 | 0.02 | PREDICTED: spatacsin isoform X1 [Canis lupus familiaris] |
| **gi\|545489348** | 274118 | 0.03 | PREDICTED: WD repeat-containing protein 87 [Canis lupus familiaris] |
| **gi\|73982258** | 34069 | 0.13 | PREDICTED: thioredoxin-related transmembrane protein 2 isoform 1 [Canis lupus familiaris] |
| **gi\|73968452** | 55145 | 0.08 | PREDICTED: transmembrane protein 194A [Canis lupus familiaris] |
| **gi\|545503669** | 129290 | 0.07 | PREDICTED: nodal modulator 1 [Canis lupus familiaris] |
| **gi\|545520844** | 125995 | 0.03 | PREDICTED: arf-GAP with SH3 domain, ANK repeat and PH domain-containing protein 1 isoform X1 [Canis lupus familiaris] |
| **gi\|545537743** | 75354 | 0.12 | PREDICTED: sciellin isoform X9 [Canis lupus familiaris] |
| **gi\|545540568** | 101633 | 0.04 | PREDICTED: disks large-associated protein 4 isoform X10 [Canis lupus familiaris] |
| **gi\|545555959** | 122466 | 0.04 | PREDICTED: partitioning defective 3 homolog B isoform X6 [Canis lupus familiaris] |
| **gi\|545491435** | 63031 | 0.07 | PREDICTED: alpha-taxilin [Canis lupus familiaris] |
| **gi\|545486029** | 53932 | 0.08 | PREDICTED: transmembrane protein 200A [Canis lupus familiaris] |
| **gi\|57104550** | 55115 | 0.08 | PREDICTED: growth/differentiation factor 5 [Canis lupus familiaris] |
| **gi\|545511186** | 111858 | 0.04 | PREDICTED: LOW QUALITY PROTEIN: MYCBP-associated protein [Canis lupus familiaris] |
| **gi\|73950747** | 27566 | 0.17 | PREDICTED: transmembrane protein 51 isoform X6 [Canis lupus familiaris] |
| **gi\|545534941** | 120365 | 0.04 | PREDICTED: A disintegrin and metalloproteinase with thrombospondin motifs 10 isoform X2 [Canis lupus familiaris] |
| **gi\|73969963** | 74150 | 0.06 | PREDICTED: DCC-interacting protein 13-beta isoformX2 [Canis lupus familiaris] |
| **gi\|545495448** | 29205 | 0.16 | PREDICTED: surfactant protein A1 isoform 1 [Canis lupus familiaris] |
| **gi\|545551868** | 400894 | 0.01 | PREDICTED: WD repeat and FYVE domain-containing protein 3 isoformX1 [Canis lupus familiaris] |

**TABLE SD: Proteomics data of plasma membrane protein from HMPOS**

**
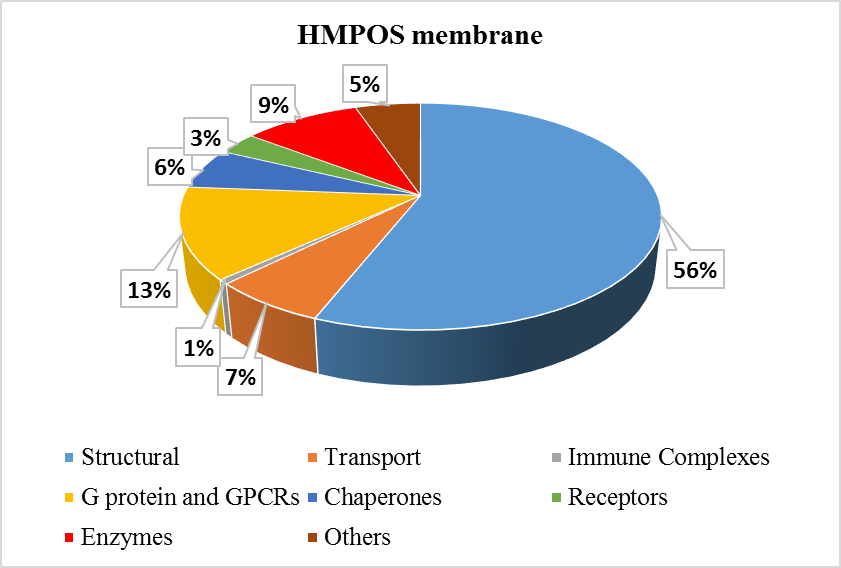
**

**TABLE SD-1. Overall proteomic profile**

|  | **emPAI** | **Percentage** |
| --- | --- | --- |
| Structural | 41.72 | 56.01 |
| Transport | 4.91 | 6.59 |
| Immune Complexes | 0.46 | 0.62 |
| G protein and GPCRs | 9.7 | 13.02 |
| Chaperones | 4.39 | 5.89 |
| Receptors | 2.41 | 3.24 |
| Enzymes | 7 | 9.40 |
| Others | 3.9 | 5.24 |
| **Total** | **74.49** | **100** |

**TABLE SD-2: Structural Proteins Identified**

| **Accession** | **Mass** | **emPAI** | **Description** |
| --- | --- | --- | --- |
| **gi\|50950177** | 38630 | 6.43 | annexin A2 [Canis lupus familiaris] |
| **gi\|61740600** | 57676 | 1.46 | keratin, type I cytoskeletal 10 [Canis lupus familiaris] |
| **gi\|545510666** | 54047 | 0.89 | PREDICTED: keratin, type I cytoskeletal 42 [Canis lupus familiaris] |
| **gi\|359279911** | 52279 | 0.94 | keratin 14 [Canis lupus familiaris] |
| **gi\|73965817** | 51649 | 0.95 | PREDICTED: keratin, type I cytoskeletal 16 [Canis lupus familiaris] |
| **gi\|359279916** | 43858 | 0.48 | keratin, type I cytoskeletal 19 [Canis lupus familiaris] |
| **gi\|545538333** | 96812 | 0.09 | PREDICTED: programmed cell death 6-interacting protein isoform X2 [Canis lupus familiaris] |
| **gi\|73974726** | 532579 | 0.21 | PREDICTED: plectin isoformX1 [Canis lupus familiaris] |
| **gi\|4501885** | 41710 | 2.82 | actin, cytoplasmic 1 [Homo sapiens] |
| **gi\|4501883** | 41982 | 1.05 | actin, aortic smooth muscle [Homo sapiens] |
| **gi\|545490700** | 37169 | 1 | PREDICTED: actin, beta-like 2 isoform X1 [Canis lupus familiaris] |
| **gi\|73973308** | 332597 | 0.44 | PREDICTED: collagen alpha-1(XII) chain isoformX1 [Canis lupus familiaris] |
| **gi\|7106439** | 49639 | 1.84 | tubulin beta-5 chain [Mus musculus] |
| **gi\|21361322** | 49554 | 1.39 | tubulin beta-4A chain isoform 3 [Homo sapiens] |
| **gi\|21746161** | 49921 | 1.18 | tubulin beta-2B chain [Mus musculus] |
| **gi\|50979272** | 63751 | 0.72 | keratin, type II cytoskeletal 1 [Canis lupus familiaris] |
| **gi\|345791904** | 62778 | 0.41 | PREDICTED: keratin, type II cytoskeletal 5 isoform X1 [Canis lupus familiaris] |
| **gi\|359323093** | 54684 | 0.37 | PREDICTED: keratin, type II cytoskeletal 8 [Canis lupus familiaris] |
| **gi\|345791839** | 62832 | 0.41 | PREDICTED: keratin, type II cytoskeletal 6A isoformX2 [Canis lupus familiaris] |
| **gi\|50979264** | 64527 | 0.31 | keratin, type II cytoskeletal 2 epidermal [Canis lupus familiaris] |
| **gi\|545545402** | 51588 | 0.18 | PREDICTED: keratin, type II cytoskeletal 7 [Canis lupus familiaris] |
| **gi\|545545370** | 63946 | 0.14 | PREDICTED: keratin, type II cytoskeletal 73 isoform X1 [Canis lupus familiaris] |
| **gi\|34740335** | 50120 | 1.17 | tubulin alpha-1B chain [Mus musculus] |
| **gi\|160425231** | 226328 | 0.21 | myosin-9 [Canis lupus familiaris] |
| **gi\|558695394** | 38602 | 0.75 | annexin A1 [Canis lupus familiaris] |
| **gi\|57100553** | 35921 | 0.82 | PREDICTED: annexin A5 [Canis lupus familiaris] |
| **gi\|74004777** | 138356 | 0.21 | PREDICTED: collagen alpha-1(III) chain isoform 2 [Canis lupus familiaris] |
| **gi\|359320831** | 269595 | 0.07 | PREDICTED: talin-1 isoform X1 [Canis lupus familiaris] |
| **gi\|5031635** | 18491 | 0.58 | cofilin-1 [Homo sapiens] |
| **gi\|74005014** | 131810 | 0.03 | PREDICTED: unconventional myosin-Ib isoformX2 [Canis lupus familiaris] |
| **gi\|545558304** | 69724 | 0.06 | PREDICTED: moesin isoform X3 [Canis lupus familiaris] |
| **gi\|460417294** | 41977 | 0.11 | actin-related protein 3 isoform 2 [Homo sapiens] |
| **gi\|545497795** | 19402 | 0.24 | PREDICTED: profilin-1 [Canis lupus familiaris] |
| **gi\|301774268** | 33202 | 0.14 | PREDICTED: tropomyosin alpha-3 chain-like isoform 1 [Ailuropoda melanoleuca] |
| **gi\|74008809** | 280450 | 0.06 | PREDICTED: filamin-A isoform 2 [Canis lupus familiaris] |
| **gi\|545494454** | 47490 | 0.1 | PREDICTED: lactadherin [Canis lupus familiaris] |
| **gi\|345782840** | 191681 | 0.02 | PREDICTED: laminin subunit beta-4 [Canis lupus familiaris] |
| **gi\|345799205** | 288872 | 0.02 | PREDICTED: LOW QUALITY PROTEIN: PDZ domain-containing protein 2 [Canis lupus familiaris] |
| **gi\|545497238** | 45641 | 0.1 | PREDICTED: LOW QUALITY PROTEIN: adhesion molecule, interacts with CXADR antigen 1 [Canis lupus familiaris] |
| **gi\|115947174** | 222863 | 0.02 | myosin-4 [Canis lupus familiaris] |
| **gi\|545510084** | 21109 | 0.22 | PREDICTED: myosin heavy chain IB-like [Canis lupus familiaris] |
| **gi\|17986258** | 16919 | 0.65 | myosin light polypeptide 6 isoform 1 [Homo sapiens] |
| **gi\|73969653** | 212837 | 0.04 | PREDICTED: girdin isoformX2 [Canis lupus familiaris] |
| **gi\|545533702** | 55298 | 0.08 | PREDICTED: amphoterin-induced protein 3 [Canis lupus familiaris] |
| **gi\|545557020** | 109812 | 0.04 | PREDICTED: contactin-2 [Canis lupus familiaris] |
| **gi\|545531544** | 208611 | 0.02 | PREDICTED: LOW QUALITY PROTEIN: SH3 and multiple ankyrin repeat domains protein 2 [Canis lupus familiaris] |
| **gi\|359322210** | 111999 | 0.04 | PREDICTED: microtubule-associated protein 1S isoform X1 [Canis lupus familiaris] |
| **gi\|73968528** | 44390 | 0.1 | PREDICTED: dynactin subunit 2 isoformX3 [Canis lupus familiaris] |
| **gi\|73987361** | 33365 | 0.14 | PREDICTED: calponin-2 isoformX1 [Canis lupus familiaris] |
| **gi\|545555092** | 390848 | 0.01 | PREDICTED: xin actin-binding repeat-containing protein 2 isoform X1 [Canis lupus familiaris] |
| **gi\|545544126** | 120433 | 0.04 | PREDICTED: unconventional myosin-Ih [Canis lupus familiaris] |
| **gi\|5031571** | 44732 | 0.1 | actin-related protein 2 isoform b [Homo sapiens] |
| **gi\|73947726** | 104337 | 0.04 | PREDICTED: alpha-actinin-4 isoformX6 [Canis lupus familiaris] |
| **gi\|545502948** | 55287 | 0.08 | PREDICTED: amphoterin-induced protein 1 [Canis lupus familiaris] |
| **gi\|545520371** | 200465 | 0.02 | PREDICTED: laminin subunit alpha-4 [Canis lupus familiaris] |
| **gi\|545493680** | 57725 | 0.08 | PREDICTED: ankyrin repeat domain-containing protein 34C [Canis lupus familiaris] |
| **gi\|545541615** | 77347 | 0.06 | PREDICTED: cytoskeleton-associated protein 2 isoform X1 [Canis lupus familiaris] |
| **gi\|545550658** | 214431 | 0.02 | PREDICTED: unconventional myosin-Vc [Canis lupus familiaris] |
| **gi\|345784082** | 555378 | 0.01 | PREDICTED: uncharacterized protein KIAA1109 [Canis lupus familiaris] |
| **gi\|545508415** | 106733 | 0.04 | PREDICTED: LOW QUALITY PROTEIN: nesprin-3 [Canis lupus familiaris] |
| **gi\|62122767** | 76308 | 0.06 | keratin, type I cytoskeletal 9 [Canis lupus familiaris] |
| **gi\|545485411** | 134816 | 0.03 | PREDICTED: jouberin isoform X1 [Canis lupus familiaris] |
| **gi\|356640238** | 43975 | 0.1 | CKLF-like MARVEL transmembrane domain-containing protein 1 [Canis lupus familiaris] |
| **gi\|345788489** | 345423 | 0.01 | PREDICTED: LOW QUALITY PROTEIN: protocadherin-16 [Canis lupus familiaris] |
| **gi\|545510084** | 21109 | 0.22 | PREDICTED: myosin heavy chain IB-like [Canis lupus familiaris] |
| **gi\|359323649** | 117106 | 0.04 | PREDICTED: SWI/SNF-related matrix-associated actin-dependent regulator of chromatin subfamily A containing DEAD/H box 1 isoform X1 [Canis lupus familiaris] |
|  |  |  |  |
| **gi\|545489749** | 257974 | 0.02 | PREDICTED: supervillin isoform X3 [Canis lupus familiaris] |
| **gi\|95925865** | 87375 | 0.05 | pinin 1 [Canis lupus familiaris] |
| **gi\|73953627** | 75842 | 0.33 | PREDICTED: annexin A6 isoformX2 [Canis lupus familiaris] |
| **gi\|545490274** | 16537 | 0.29 | PREDICTED: endothelial cell surface expressed chemotaxis and apoptosis regulator isoform X2 [Canis lupus familiaris] |
| **gi\|2499844** | 38043 | 0.12 | RecName: Full=CD44 antigen; AltName: Full=Extracellular matrix receptor-III; Short=ECMR-III; AltName: Full=GP90 lymphocyte homing/adhesion receptor; AltName: Full=HUTCH-I; AltName: Full=Hermes antigen; AltName: Full=Hyaluronate receptor; AltName: Fu |

**TABLE SD-3: Transport Proteins Identified**

| **Accession** | **Mass** | **emPAI** | **Description** |
| --- | --- | --- | --- |
| **gi\|73953093** | 31559 | 1.26 | PREDICTED: voltage-dependent anion-selective channel protein 2 isoform 2 [Canis lupus familiaris] |
| **gi\|126723018** | 30722 | 0.32 | voltage-dependent anion-selective channel protein 1 [Oryctolagus cuniculus] |
| **gi\|345805068** | 90259 | 0.05 | PREDICTED: transient receptor potential cation channel subfamily V member 3 isoform X2 [Canis lupus familiaris] |
| **gi\|73997059** | 47923 | 0.09 | PREDICTED: ATP-sensitive inward rectifier potassium channel 8 isoformX1 [Canis lupus familiaris] |
| **gi\|545528359** | 112738 | 0.47 | PREDICTED: sodium/potassium-transporting ATPase subunit alpha-1 isoform X1 [Canis lupus familiaris] |
| **gi\|73963665** | 24765 | 0.68 | PREDICTED: transmembrane emp24 domain-containing protein 10 isoform 1 [Canis lupus familiaris] |
| **gi\|354459073** | 30749 | 0.15 | voltage-dependent anion-selective channel protein 3 isoform 1 [Canis lupus familiaris] |
| **gi\|345787913** | 67489 | 0.07 | PREDICTED: sodium-coupled monocarboxylate transporter 2 isoform 1 [Canis lupus familiaris] |
| **gi\|545495952** | 43843 | 0.1 | PREDICTED: magnesium transporter NIPA4 [Canis lupus familiaris] |
| **gi\|73997059** | 47923 | 0.09 | PREDICTED: ATP-sensitive inward rectifier potassium channel 8 isoformX1 [Canis lupus familiaris] |
| **gi\|545495894** | 175252 | 0.05 | PREDICTED: probable phospholipid-transporting ATPase VB isoform X1 [Canis lupus familiaris] |
| **gi\|5031569** | 42587 | 0.11 | alpha-centractin [Homo sapiens] |
| **gi\|74008791** | 71836 | 0.06 | PREDICTED: sodium- and chloride-dependent neutral and basic amino acid transporter B(0+) [Canis lupus familiaris] |
| **gi\|73945930** | 58023 | 0.08 | PREDICTED: protein ERGIC-53 isoform X2 [Canis lupus familiaris] |
| **gi\|545560056** | 17447 | 0.28 | PREDICTED: proteolipid protein 2 [Canis lupus familiaris] |
| **gi\|545497820** | 149551 | 0.03 | PREDICTED: LOW QUALITY PROTEIN: misshapen-like kinase 1 [Canis lupus familiaris] |
| **gi\|345792321** | 15429 | 0.31 | PREDICTED: vesicle transport protein GOT1B [Canis lupus familiaris] |
| **gi\|345805068** | 90259 | 0.05 | PREDICTED: transient receptor potential cation channel subfamily V member 3 isoform X2 [Canis lupus familiaris] |
| **gi\|545531227** | 579498 | 0.02 | PREDICTED: LOW QUALITY PROTEIN: neuroblast differentiation-associated protein AHNAK [Canis lupus familiaris] |
| **gi\|73983713** | 85720 | 0.05 | PREDICTED: vacuolar protein sorting-associated protein 51 homolog [Canis lupus familiaris] |
| **gi\|73979536** | 31206 | 0.15 | PREDICTED: neuronal membrane glycoprotein M6-a isoform 1 [Canis lupus familiaris] |
| **gi\|545522646** | 121825 | 0.04 | PREDICTED: LOW QUALITY PROTEIN: multidrug resistance protein 1 [Canis lupus familiaris] |
| **gi\|50978854** | 61427 | 0.07 | excitatory amino acid transporter 4 [Canis lupus familiaris] |
| **gi\|50979327** | 50151 | 0.09 | equilibrative nucleoside transporter 1 [Canis lupus familiaris] |
| **gi\|545539571** | 29641 | 0.16 | PREDICTED: sodium/potassium-transporting ATPase subunit beta-3, partial [Canis lupus familiaris] |
| **gi\|73956324** | 58186 | 0.08 | PREDICTED: non-specific lipid-transfer protein isoformX3 [Canis lupus familiaris] |

**TABLE SD-3: Immune Complexes identified**

| **Accession** | **Mass** | **emPAI** | **Description** |
| --- | --- | --- | --- |
| **gi\|345807015** | 79859 | 0.06 | PREDICTED: interleukin 1 receptor accessory protein-like 1 [Canis lupus familiaris] |
| **gi\|3334903** | 47844 | 0.09 | endothelin receptor B [Canis lupus familiaris] |
| **gi\|545548378** | 118345 | 0.04 | PREDICTED: VPS10 domain-containing receptor SorCS1 [Canis lupus familiaris] |
| **gi\|345787413** | 66034 | 0.07 | PREDICTED: coiled-coil domain-containing protein 36 isoform X1 [Canis lupus familiaris] |
| **gi\|345797884** | 43717 | 0.1 | PREDICTED: V-set and immunoglobulin domain-containing protein 8 isoform X1 [Canis lupus familiaris] |
| **gi\|545515158** | 45807 | 0.1 | PREDICTED: LIM and senescent cell antigen-like domains 1 isoform X5 [Canis lupus familiaris] |

**ST- 4-5: GPCRs and G proteins identified**

| **Accession** | **Mass** | **emPAI** | **Description** |
| --- | --- | --- | --- |
| **gi\|74009012** | 328348 | 0.03 | PREDICTED: probable G-protein coupled receptor 112 [Canis lupus familiaris] |
| **gi\|545546307** | 47526 | 0.1 | PREDICTED: probable G-protein coupled receptor 19 isoform X2 [Canis lupus familiaris] |
| **gi\|345807071** | 38241 | 0.12 | PREDICTED: probable G-protein coupled receptor 82 [Canis lupus familiaris] |
| **gi\|345783859** | 35013 | 0.13 | PREDICTED: olfactory receptor 4C11-like [Canis lupus familiaris] |
| **gi\|54607157** | 52344 | 0.09 | 5-hydroxytryptamine receptor 2A [Canis lupus familiaris] |
| **gi\|305410870** | 21597 | 0.48 | membrane-associated progesterone receptor component 1 [Canis lupus familiaris] |
| **gi\|545531462** | 41785 | 0.11 | PREDICTED: LOW QUALITY PROTEIN: olfactory receptor 5AK2 [Canis lupus familiaris] |
| **gi\|345783868** | 35060 | 0.13 | PREDICTED: olfactory receptor 4C16-like [Canis lupus familiaris] |
| **gi\|545525474** | 51582 | 0.09 | PREDICTED: muscarinic acetylcholine receptor M2 isoform X1 [Canis lupus familiaris] |
| **gi\|545506467** | 50860 | 0.09 | PREDICTED: LOW QUALITY PROTEIN: guanine nucleotide-binding protein G(olf) subunit alpha [Canis lupus familiaris] |
| **gi\|5174447** | 35055 | 0.44 | guanine nucleotide-binding protein subunit beta-2-like 1 [Homo sapiens] |
| **gi\|6680045** | 37353 | 0.12 | guanine nucleotide-binding protein G(I)/G(S)/G(T) subunit beta-1 [Mus musculus] |
| **gi\|5174447** | 35055 | 0.44 | guanine nucleotide-binding protein subunit beta-2-like 1 [Homo sapiens] |
| **gi\|73985077** | 21585 | 0.22 | PREDICTED: PRA1 family protein 3 [Canis lupus familiaris] |
| **gi\|4506365** | 23531 | 0.2 | ras-related protein Rab-2A isoform a [Homo sapiens] |
| **gi\|4502201** | 20684 | 0.51 | ADP-ribosylation factor 1 [Homo sapiens] |
| **gi\|6680045** | 37353 | 0.12 | guanine nucleotide-binding protein G(I)/G(S)/G(T) subunit beta-1 [Mus musculus] |
| **gi\|545532122** | 103408 | 0.04 | PREDICTED: rab3 GTPase-activating protein catalytic subunit isoform X7 [Canis lupus familiaris] |
| **gi\|359322679** | 79430 | 0.06 | PREDICTED: LOW QUALITY PROTEIN: rho GTPase-activating protein 40 [Canis lupus familiaris] |
| **gi\|345783868** | 35060 | 0.13 | PREDICTED: olfactory receptor 4C16-like [Canis lupus familiaris] |
| **gi\|4758796** | 40517 | 0.11 | developmentally-regulated GTP-binding protein 1 [Homo sapiens] |
| **gi\|73964747** | 23379 | 0.2 | PREDICTED: rho GDP-dissociation inhibitor 1 isoform 2 [Canis lupus familiaris] |
| **gi\|545514178** | 82795 | 0.05 | PREDICTED: arf-GAP with GTPase, ANK repeat and PH domain-containing protein 2 [Canis lupus familiaris] |
| **gi\|345783859** | 35013 | 0.13 | PREDICTED: olfactory receptor 4C11-like [Canis lupus familiaris] |
| **gi\|545546307** | 47526 | 0.1 | PREDICTED: probable G-protein coupled receptor 19 isoform X2 [Canis lupus familiaris] |
| **gi\|73991970** | 166752 | 0.03 | PREDICTED: ral GTPase-activating protein subunit beta isoformX1 [Canis lupus familiaris] |
| **gi\|345807071** | 38241 | 0.12 | PREDICTED: probable G-protein coupled receptor 82 [Canis lupus familiaris] |
| **gi\|545531462** | 41785 | 0.11 | PREDICTED: LOW QUALITY PROTEIN: olfactory receptor 5AK2 [Canis lupus familiaris] |
| **gi\|545506467** | 50860 | 0.09 | PREDICTED: LOW QUALITY PROTEIN: guanine nucleotide-binding protein G(olf) subunit alpha [Canis lupus familiaris] |
| **gi\|73997540** | 127936 | 0.03 | PREDICTED: ELKS/Rab6-interacting/CAST family member 1 isoformX1 [Canis lupus familiaris] |
| **gi\|345780300** | 53816 | 0.08 | PREDICTED: beta-chimaerin isoform 2 [Canis lupus familiaris] |
| **gi\|545546746** | 51893 | 0.09 | PREDICTED: tubby-related protein 3 isoform X4 [Canis lupus familiaris] |
| **gi\|545516757** | 51263 | 0.09 | PREDICTED: septin-8 isoform X1 [Canis lupus familiaris] |
| **gi\|545545931** | 101735 | 0.04 | PREDICTED: FYVE, RhoGEF and PH domain-containing protein 4 isoform X2 [Canis lupus familiaris] |
| **gi\|73951551** | 189049 | 0.02 | PREDICTED: IQ motif containing GTPase activating protein 1 [Canis lupus familiaris] |
| **gi\|73965285** | 93254 | 0.05 | PREDICTED: axin-2 isoformX1 [Canis lupus familiaris] |
| **gi\|545509639** | 71038 | 0.06 | PREDICTED: septin-9 [Canis lupus familiaris] |
| **gi\|3334903** | 47844 | 0.09 | endothelin receptor B [Canis lupus familiaris] |
|  |  |  |  |
| **gi\|131804** | 22555 | 0.76 | RecName: Full=Ras-related protein Rab-10 [Canis lupus familiaris] |
| **gi\|4506413** | 20974 | 0.5 | ras-related protein Rap-1A precursor [Homo sapiens] |
| **gi\|50979062** | 23441 | 0.2 | ras-related protein Rab-5C [Canis lupus familiaris] |
| **gi\|50979156** | 23505 | 0.72 | ras-related protein Rab-7a [Canis lupus familiaris] |
| **gi\|345799503** | 52039 | 0.18 | PREDICTED: ras GTPase-activating protein-binding protein 1 isoform 4 [Canis lupus familiaris] |
| **gi\|50979150** | 23644 | 0.2 | ras-related protein Rab-5A [Canis lupus familiaris] |
| **gi\|545489193** | 52983 | 0.08 | PREDICTED: SHC-transforming protein 3 [Canis lupus familiaris] |

**TABLE SD-6 Chaperone Proteins Identified**

| **Accession** | **Mass** | **emPAI** | **Description** |
| --- | --- | --- | --- |
| **gi\|57032236** | 60216 | 0.54 | PREDICTED: T-complex protein 1 subunit alpha isoformX1 [Canis lupus familiaris] |
| **gi\|359320591** | 58002 | 0.45 | PREDICTED: T-complex protein 1 subunit delta isoform X1 [Canis lupus familiaris] |
| **gi\|545500762** | 58230 | 0.45 | PREDICTED: T-complex protein 1 subunit zeta [Canis lupus familiaris] |
| **gi\|345805760** | 53551 | 0.38 | PREDICTED: T-complex protein 1 subunit zeta-2 isoform X3 [Canis lupus familiaris] |
| **gi\|345802573** | 60589 | 0.33 | PREDICTED: T-complex protein 1 subunit gamma isoform 1 [Canis lupus familiaris] |
| **gi\|545550950** | 77756 | 0.18 | PREDICTED: T-complex protein 1 subunit theta isoform X2 [Canis lupus familiaris] |
| **gi\|73968673** | 57395 | 0.35 | PREDICTED: T-complex protein 1 subunit beta isoformX1 [Canis lupus familiaris] |
| **gi\|359323746** | 59559 | 0.16 | PREDICTED: LOW QUALITY PROTEIN: T-complex protein 1 subunit epsilon isoform 1 [Canis lupus familiaris] |
| **gi\|545558786** | 48739 | 0.09 | PREDICTED: LOW QUALITY PROTEIN: t-complex 11 family, X-linked 2 [Canis lupus familiaris] |
| **gi\|126352614** | 83185 | 0.6 | heat shock protein HSP 90-beta [Equus caballus] |
| **gi\|545508859** | 72881 | 0.52 | PREDICTED: heat shock protein HSP 90-alpha, partial [Canis lupus familiaris] |

**TABLE SD-7 Receptors Identified**

| **Accession** | **Mass** | **emPAI** | **Description** |
| --- | --- | --- | --- |
| **gi\|50978812** | 86594 | 0.11 | transferrin receptor protein 1 [Canis lupus familiaris] |
| **gi\|50979256** | 122944 | 0.04 | platelet-derived growth factor receptor beta precursor [Canis lupus familiaris] |
| **gi\|345806764** | 118927 | 0.04 | PREDICTED: toll-like receptor 8 isoform X1 [Canis lupus familiaris] |
| **gi\|50979256** | 122944 | 0.04 | platelet-derived growth factor receptor beta precursor [Canis lupus familiaris] |
| **gi\|73987612** | 29284 | 0.34 | PREDICTED: basigin [Canis lupus familiaris] |
| **gi\|545509559** | 20851 | 0.23 | PREDICTED: CMRF35-like molecule 7-like [Canis lupus familiaris] |
| **gi\|73979339** | 56856 | 0.08 | PREDICTED: TELO2-interacting protein 2 isoform X2 [Canis lupus familiaris] |
| **gi\|545548378** | 118345 | 0.04 | PREDICTED: VPS10 domain-containing receptor SorCS1 [Canis lupus familiaris] |
| **gi\|305410870** | 21597 | 0.48 | membrane-associated progesterone receptor component 1 [Canis lupus familiaris] |
| **gi\|545501026** | 56949 | 0.08 | PREDICTED: BAI1-associated protein 2-like 1 isoformX1 [Canis lupus familiaris] |
| **gi\|54607157** | 52344 | 0.09 | 5-hydroxytryptamine receptor 2A [Canis lupus familiaris] |
| **gi\|545546046** | 64112 | 0.07 | PREDICTED: transmembrane 7 superfamily member 3 isoform X1 [Canis lupus familiaris] |
| **gi\|545528700** | 27220 | 0.17 | PREDICTED: hemojuvelin isoform X3 [Canis lupus familiaris] |
| **gi\|345777328** | 131191 | 0.03 | PREDICTED: LOW QUALITY PROTEIN: reticulon-4 isoform 1 [Canis lupus familiaris] |
| **gi\|545507543** | 165246 | 0.03 | PREDICTED: leucine-rich repeat-containing protein 9-like isoform X3 [Canis lupus familiaris] |
| **gi\|57089795** | 32521 | 0.14 | PREDICTED: vesicle-associated membrane protein-associated protein A isoform 1 [Canis lupus familiaris] |
| **gi\|74008133** | 117410 | 0.04 | PREDICTED: angiomotin isoformX1 [Canis lupus familiaris] |
| **gi\|545518973** | 88005 | 0.05 | PREDICTED: leucine-rich repeat and fibronectin type-III domain-containing protein 2 [Canis lupus familiaris] |
| **gi\|73966275** | 63426 | 0.07 | PREDICTED: insulin-like growth factor 2 mRNA binding protein 1 isoformX2 [Canis lupus familiaris] |
| **gi\|545514112** | 60102 | 0.07 | PREDICTED: receptor-type tyrosine-protein phosphatase R isoform X1 [Canis lupus familiaris] |
| **gi\|545548312** | 89310 | 0.05 | PREDICTED: receptor-type tyrosine-protein phosphatase epsilon isoform X5 [Canis lupus familiaris] |
| **gi\|359318678** | 564218 | 0.01 | PREDICTED: ryanodine receptor 1 isoform 1 [Canis lupus familiaris] |
| **gi\|545531508** | 146992 | 0.03 | PREDICTED: receptor-type tyrosine-protein phosphatase eta [Canis lupus familiaris] |
| **gi\|545531508** | 146992 | 0.03 | PREDICTED: receptor-type tyrosine-protein phosphatase eta [Canis lupus familiaris] |
| **gi\|345796108** | 87112 | 0.05 | PREDICTED: integrin beta-5 [Canis lupus familiaris] |

**TABLE SD-8 Enzymes Identified**

| **Accession** | **Mass** | **emPAI** | **Description** |
| --- | --- | --- | --- |
| **gi\|545535631** | 133147 | 0.03 | PREDICTED: LOW QUALITY PROTEIN: non-receptor tyrosine-protein kinase TYK2 [Canis lupus familiaris] |
| **gi\|73980394** | 48268 | 0.87 | PREDICTED: protein disulfide-isomerase A6 [Canis lupus familiaris] |
| **gi\|73964749** | 57381 | 1.12 | PREDICTED: protein disulfide-isomerase [Canis lupus familiaris] |
| **gi\|345800677** | 48956 | 0.55 | PREDICTED: LOW QUALITY PROTEIN: alpha-enolase isoform 1 [Canis lupus familiaris] |
| **gi\|545547462** | 87343 | 0.22 | PREDICTED: delta-1-pyrroline-5-carboxylate synthase isoform X3 [Canis lupus familiaris] |
| **gi\|74000476** | 23761 | 0.43 | PREDICTED: peptidyl-prolyl cis-trans isomerase B isoform 2 [Canis lupus familiaris] |
| **gi\|545539262** | 87576 | 0.22 | PREDICTED: procollagen-lysine,2-oxoglutarate 5-dioxygenase 2 isoform X2 [Canis lupus familiaris] |
| **gi\|359321459** | 72351 | 0.13 | PREDICTED: protein disulfide-isomerase A4 isoform 3 [Canis lupus familiaris] |
| **gi\|545512792** | 124956 | 0.04 | PREDICTED: protein phosphatase 1 regulatory subunit 26 isoform X1 [Canis lupus familiaris] |
| **gi\|345802152** | 68262 | 0.07 | PREDICTED: serine/threonine-protein kinase PLK1 [Canis lupus familiaris] |
| **gi\|345799467** | 71888 | 0.06 | PREDICTED: tyrosine-protein kinase ITK/TSK [Canis lupus familiaris] |
| **gi\|73954910** | 52795 | 0.09 | PREDICTED: serine/threonine-protein kinase Chk1 [Canis lupus familiaris] |
| **gi\|545548312** | 89310 | 0.05 | PREDICTED: receptor-type tyrosine-protein phosphatase epsilon isoform X5 [Canis lupus familiaris] |
| **gi\|545557037** | 116529 | 0.04 | PREDICTED: serine/threonine-protein kinase MARK1 [Canis lupus familiaris] |
| **gi\|545555517** | 3901076 | 0 | PREDICTED: LOW QUALITY PROTEIN: titin [Canis lupus familiaris] |
| **gi\|73980965** | 92301 | 0.05 | PREDICTED: mannosyl-oligosaccharide glucosidase [Canis lupus familiaris] |
| **gi\|6755588** | 23300 | 0.2 | synaptosomal-associated protein 25 isoform a [Mus musculus] |
| **gi\|545521006** | 120316 | 0.04 | PREDICTED: focal adhesion kinase 1 isoform X3 [Canis lupus familiaris] |
| **gi\|308082020** | 26698 | 0.17 | triosephosphate isomerase [Canis lupus familiaris] |
| **gi\|345803586** | 35294 | 0.13 | PREDICTED: retinol dehydrogenase 11 isoform X3 [Canis lupus familiaris] |
| **gi\|345804359** | 31495 | 0.15 | PREDICTED: thioredoxin-related transmembrane protein 1 [Canis lupus familiaris] |
| **gi\|345782889** | 204305 | 0.02 | PREDICTED: LOW QUALITY PROTEIN: histone-lysine N-methyltransferase 2E isoform 1 [Canis lupus familiaris] |
| **gi\|545489174** | 204718 | 0.02 | PREDICTED: proprotein convertase subtilisin/kexin type 5 [Canis lupus familiaris] |
| **gi\|545535534** | 158607 | 0.03 | PREDICTED: membrane-associated guanylate kinase, WW and PDZ domain-containing protein 1 [Canis lupus familiaris] |
| **gi\|57110216** | 43734 | 0.1 | PREDICTED: p21-activated protein kinase-interacting protein 1 [Canis lupus familiaris] |
| **gi\|74006997** | 27138 | 0.17 | PREDICTED: 3-hydroxyacyl-CoA dehydrogenase type-2 isoform 1 [Canis lupus familiaris] |
| **gi\|545514789** | 72610 | 0.06 | PREDICTED: TGF-beta-activated kinase 1 and MAP3K7-binding protein 1 isoformX1 [Canis lupus familiaris] |
| **gi\|73966592** | 133435 | 0.03 | PREDICTED: myotubularin-related protein 4 isoformX1 [Canis lupus familiaris] |
| **gi\|73958481** | 39478 | 0.12 | PREDICTED: fructose-bisphosphate aldolase A isoformX2 [Canis lupus familiaris] |
| **gi\|545544113** | 42798 | 0.11 | PREDICTED: mevalonate kinase isoform X1 [Canis lupus familiaris] |
| **gi\|532164718** | 54008 | 0.08 | serine/threonine-protein phosphatase 2A 56 kDa regulatory subunit epsilon isoform isoform b [Homo sapiens] |
| **gi\|73967304** | 152838 | 0.03 | PREDICTED: carboxypeptidase D isoform 2 [Canis lupus familiaris] |
| **gi\|347889344** | 97560 | 0.05 | thyrotropin-releasing hormone-degrading ectoenzyme [Canis lupus familiaris] |
| **gi\|545497443** | 77070 | 0.06 | PREDICTED: serine/threonine-protein phosphatase 2A 65 kDa regulatory subunit A beta isoform isoform X4 [Canis lupus familiaris] |
| **gi\|545535631** | 133147 | 0.03 | PREDICTED: LOW QUALITY PROTEIN: non-receptor tyrosine-protein kinase TYK2 [Canis lupus familiaris] |
| **gi\|74002818** | 57933 | 0.08 | PREDICTED: serine/threonine-protein kinase PAK 2 isoform 1 [Canis lupus familiaris] |
| **gi\|73975797** | 40015 | 0.11 | PREDICTED: serum paraoxonase/arylesterase 1 isoform 2 [Canis lupus familiaris] |
| **gi\|73978267** | 40232 | 0.11 | PREDICTED: glycosyltransferase 8 domain-containing protein 2 isoform X4 [Canis lupus familiaris] |
| **gi\|345790993** | 51995 | 0.09 | PREDICTED: adenosine monophosphate-protein transferase FICD isoform X3 [Canis lupus familiaris] |
| **gi\|545511519** | 105714 | 0.04 | PREDICTED: E3 ubiquitin-protein ligase TRIM37 isoform X3 [Canis lupus familiaris] |
| **gi\|545508117** | 148451 | 0.03 | PREDICTED: tubulin polyglutamylase TTLL5 isoform X1 [Canis lupus familiaris] |
| **gi\|545516121** | 77140 | 0.06 | PREDICTED: glutamine--fructose-6-phosphate aminotransferase [isomerizing] 2 [Canis lupus familiaris] |
| **gi\|73970573** | 45905 | 0.1 | PREDICTED: protein-lysine 6-oxidase isoformX1 [Canis lupus familiaris] |
| **gi\|73983265** | 138671 | 0.03 | PREDICTED: 1-phosphatidylinositol 4,5-bisphosphate phosphodiesterase beta-3 isoformX2 [Canis lupus familiaris] |
| **gi\|74008407** | 40859 | 0.11 | PREDICTED: palmitoyltransferase ZDHHC9 isoform 2 [Canis lupus familiaris] |
| **gi\|57098955** | 44852 | 0.1 | PREDICTED: ceramide synthase 2 isoform 1 [Canis lupus familiaris] |
| **gi\|545546189** | 73137 | 0.06 | PREDICTED: 1-phosphatidylinositol 4,5-bisphosphate phosphodiesterase zeta-1 isoform X1 [Canis lupus familiaris] |
| **gi\|359323381** | 134236 | 0.07 | PREDICTED: 1-phosphatidylinositol 4,5-bisphosphate phosphodiesterase beta-2 isoform X1 [Canis lupus familiaris] |
| **gi\|171184453** | 17118 | 0.28 | prostaglandin E synthase [Canis lupus familiaris] |
| **gi\|545558971** | 77661 | 0.06 | PREDICTED: long-chain-fatty-acid--CoA ligase 4 isoform X5 [Canis lupus familiaris] |
| **gi\|545529305** | 88247 | 0.05 | PREDICTED: calcium-independent phospholipase A2-gamma isoform X2 [Canis lupus familiaris] |
| **gi\|359323612** | 82513 | 0.05 | PREDICTED: lanosterol synthase isoform 2 [Canis lupus familiaris] |

**TABLE SD-9: Other Proteins Identified**

| **Accession** | **Mass** | **emPAI** | **Description** |
| --- | --- | --- | --- |
| **gi\|73969959** | 65382 | 1.52 | PREDICTED: cytoskeleton-associated protein 4 [Canis lupus familiaris] |
| **gi\|345779293** | 27846 | 0.17 | PREDICTED: transmembrane protein 51 [Canis lupus familiaris] |
| **gi\|73971240** | 38546 | 0.25 | PREDICTED: stomatin (EPB72)-like 2 isoform 1 [Canis lupus familiaris] |
| **gi\|73983875** | 26187 | 0.18 | PREDICTED: transmembrane protein 109 isoform X4 [Canis lupus familiaris] |
| **gi\|73972968** | 114692 | 0.04 | PREDICTED: GLTSCR1-like isoform X3 [Canis lupus familiaris] |
| **gi\|345792510** | 118933 | 0.04 | PREDICTED: LOW QUALITY PROTEIN: kinesin family member 11 [Canis lupus familiaris] |
| **gi\|71043798** | 44292 | 0.1 | cathepsin D precursor [Canis lupus familiaris] |
| **gi\|545497651** | 120425 | 0.04 | PREDICTED: myb-binding protein 1A [Canis lupus familiaris] |
| **gi\|73984530** | 44862 | 0.21 | PREDICTED: transmembrane protein 43 isoform 1 [Canis lupus familiaris] |
| **gi\|545508686** | 36342 | 0.13 | PREDICTED: inverted formin-2 isoform X1 [Canis lupus familiaris] |
| **gi\|345777168** | 195291 | 0.02 | PREDICTED: GRIP and coiled-coil domain-containing protein 2 [Canis lupus familiaris] |
| **gi\|4502549** | 16827 | 0.29 | calmodulin [Homo sapiens] |
| **gi\|1353187** | 47337 | 0.1 | RecName: Full=Zona pellucida sperm-binding protein 3; AltName: Full=Sperm receptor; AltName: Full=Zona pellucida glycoprotein 3; Short=Zp-3; AltName: Full=Zona pellucida protein C; Contains: RecName: Full=Processed zona pellucida sperm-binding prote |
| **gi\|545492234** | 473796 | 0.01 | PREDICTED: LOW QUALITY PROTEIN: basement membrane-specific heparan sulfate proteoglycan core protein [Canis lupus familiaris] |
| **gi\|345800374** | 42267 | 0.11 | PREDICTED: plasminogen activator inhibitor 1 RNA-binding protein isoform 1 [Canis lupus familiaris] |
| **gi\|545511186** | 111858 | 0.04 | PREDICTED: LOW QUALITY PROTEIN: MYCBP-associated protein [Canis lupus familiaris] |
| **gi\|545502406** | 43027 | 0.11 | PREDICTED: clusterin-associated protein 1 isoform X2 [Canis lupus familiaris] |
| **gi\|73977162** | 78669 | 0.06 | PREDICTED: neurochondrin isoform 2 [Canis lupus familiaris] |
| **gi\|359321386** | 149095 | 0.03 | PREDICTED: LOW QUALITY PROTEIN: WD repeat-containing protein 65 [Canis lupus familiaris] |
| **gi\|73972924** | 37815 | 0.12 | PREDICTED: protein YIPF3 isoform 1 [Canis lupus familiaris] |
| **gi\|545488074** | 63561 | 0.07 | PREDICTED: coiled-coil domain-containing protein 61 isoform X1 [Canis lupus familiaris] |
| **gi\|545489280** | 127559 | 0.03 | PREDICTED: LOW QUALITY PROTEIN: liprin-alpha-3 [Canis lupus familiaris] |
| **gi\|545520271** | 61863 | 0.07 | PREDICTED: butyrophilin subfamily 1 member A1-like [Canis lupus familiaris] |
| **gi\|345786928** | 153318 | 0.03 | PREDICTED: LOW QUALITY PROTEIN: calcium-dependent secretion activator 1 isoform 3 [Canis lupus familiaris] |
| **gi\|73998896** | 129755 | 0.03 | PREDICTED: PDZ domain-containing protein 8 isoform X1 [Canis lupus familiaris] |

**Parameters for Confocal Microscopy:**

CH1: DAPI staining, CH2: secondary antibody staining

1. **CD44**

HOS and 143B

CH1- λ=405, P= 4.5, HV=150 OFFSET= 20

CH2- λ=561, P= 5.6, HV=41 OFFSET= -3

POS and HMPOS

CH1- λ=405, P= 25, HV=165 OFFSET= 3

CH2- λ=4561, P= 2.3, HV=93 OFFSET= -26

1. **CD147**

HOS and 143B

CH1- λ=405, P= 5.5, HV=143 OFFSET= 20

CH2- λ=488, P= 1.1, HV=51 OFFSET= 0

POS and HMPOS

CH1- λ=405, P= 100, HV=82 OFFSET= -5

CH2- λ=488, P= 2.1, HV=10 OFFSET= 0

1. **Vimentin**

HOS and 143B

CH1- λ=405, P= 6.4, HV=142 OFFSET= 0

CH2- λ=488, P= 0.7, HV=24 OFFSET= 0

POS and HMPOS

CH1- λ=405, P= 4.5, HV=114 OFFSET= 0

CH2- λ=488, P= 1.1, HV=11 OFFSET= 0

**
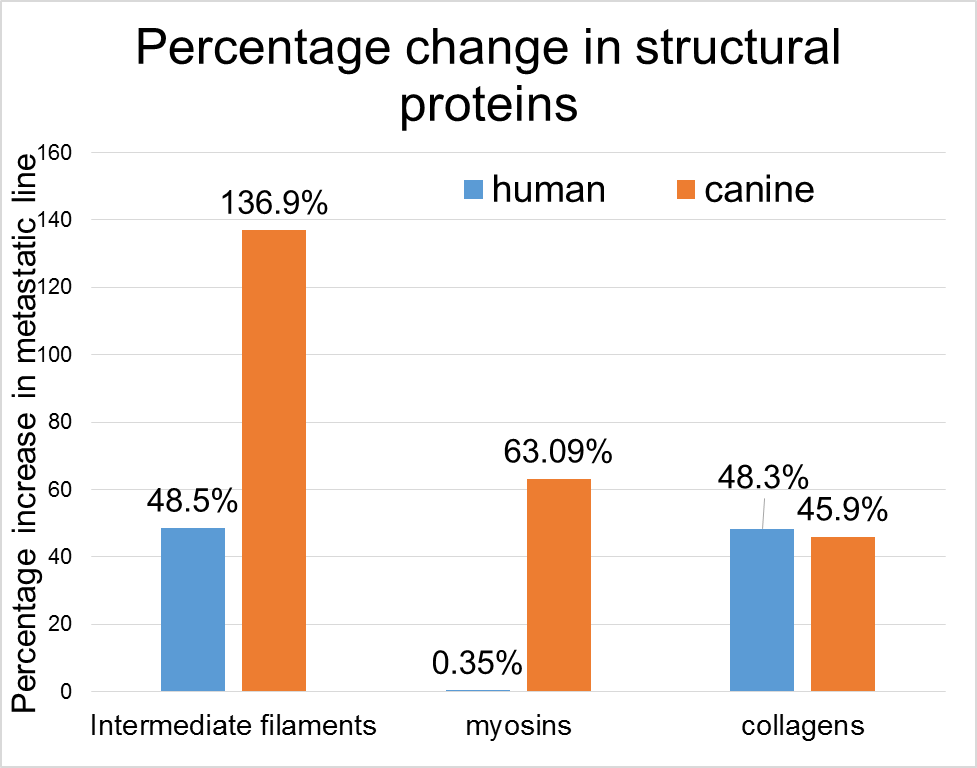
**

**Fig B-** Relative expression of structural proteins in metastatic versus non- metastatic cells as observed by peptide fingerprinting. The percentage increase in the expression in the metastatic cells as compared to the corresponding non-metastatic cells is represented.


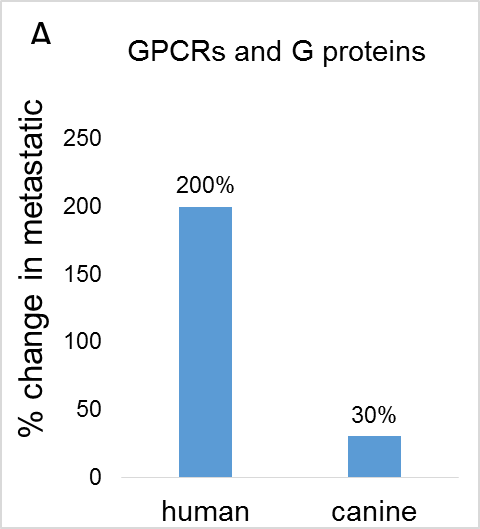

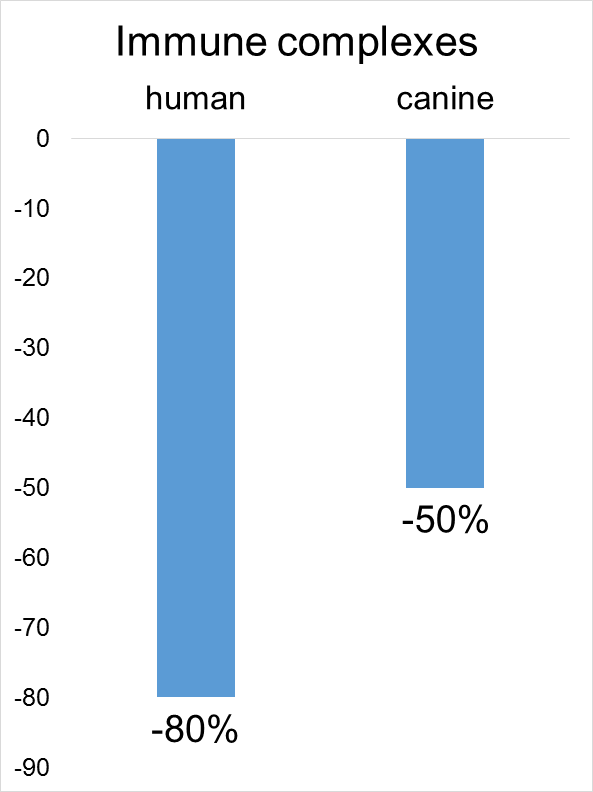


B

**Fig C-Change in expression of (A) Immune complexes and (B) GPCRs in metastatic versus corresponding non-metastatic cells**

**
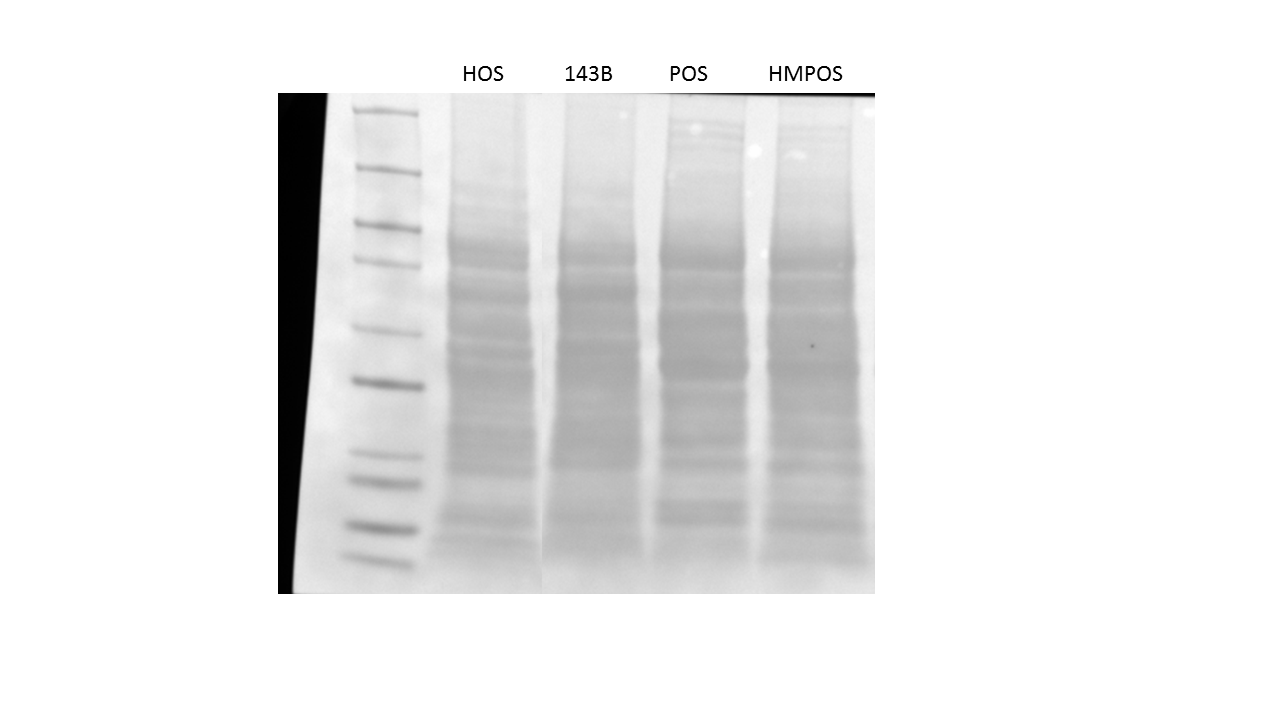
**

**Fig D:** Ponceau staining as a loading control for western blot showing equal loading of protein. Total area under each cell line is quantified in ImageLab and used to normalize the quantification of western blot staining intensity. This is performed since majority of the membrane proteins identified have been linked to cancerous states and differential regulation and thus can’t be appropriate loading controls. Additionally, actin or GAPDH are not membrane proteins and cannot be utilized to standardize these either.


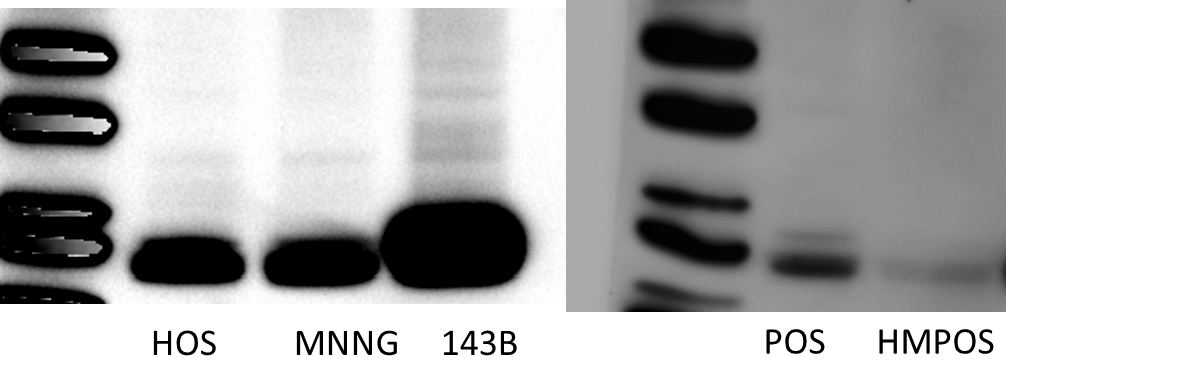


**Fig E-** Western blot showing overexpression of KRAS in 143B vs HOS. 143B shows greater expression of KRAS than HOS and MNNG (non-metastatic human OS cell line).

**
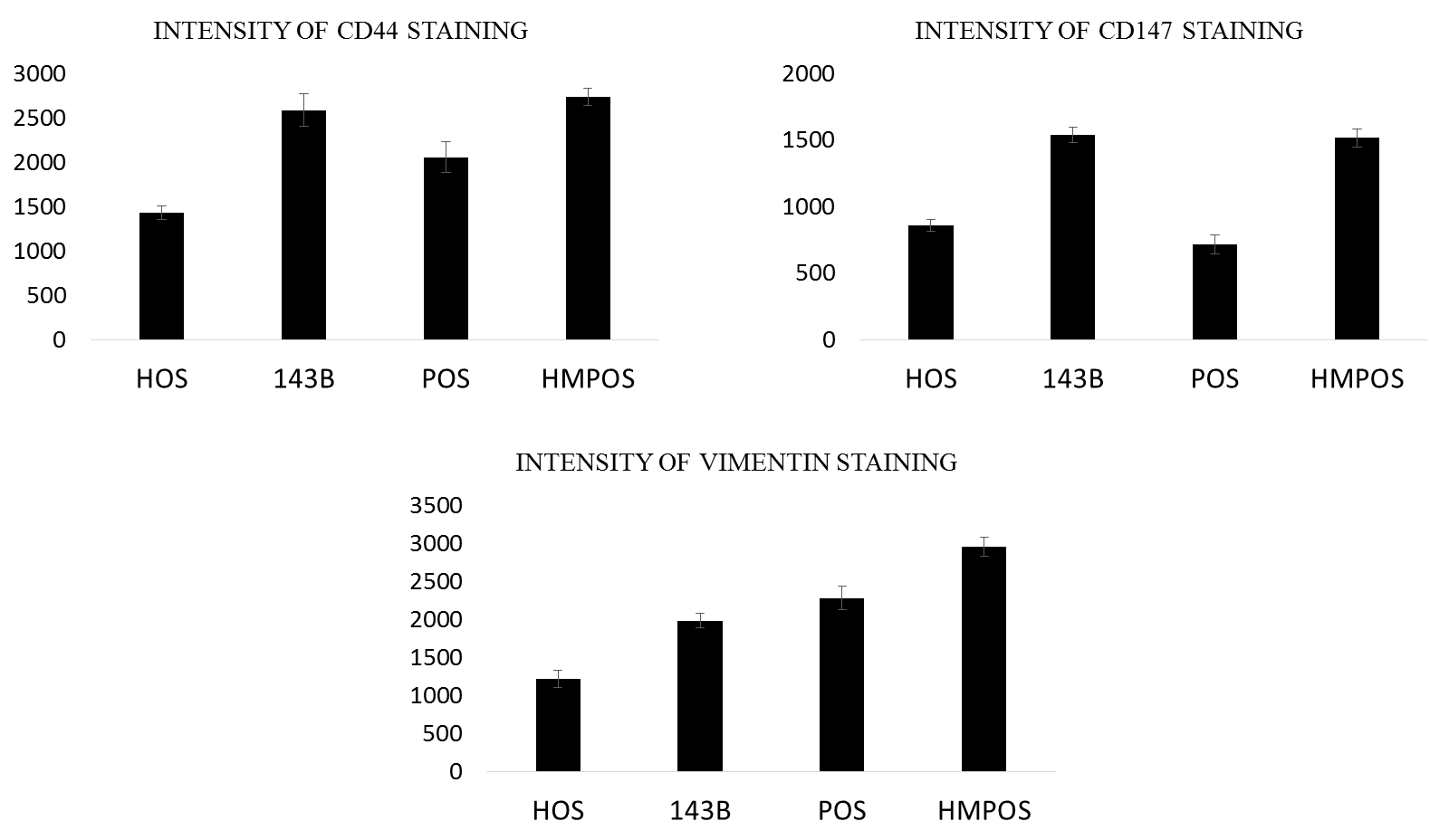
**

**Fig F:** Fig shows the ImageJ quantification of confocal microscopy images. Quantification was performed by measuring staining intensity of 20 random cells each in 2 different slides. The images are Fig 4.


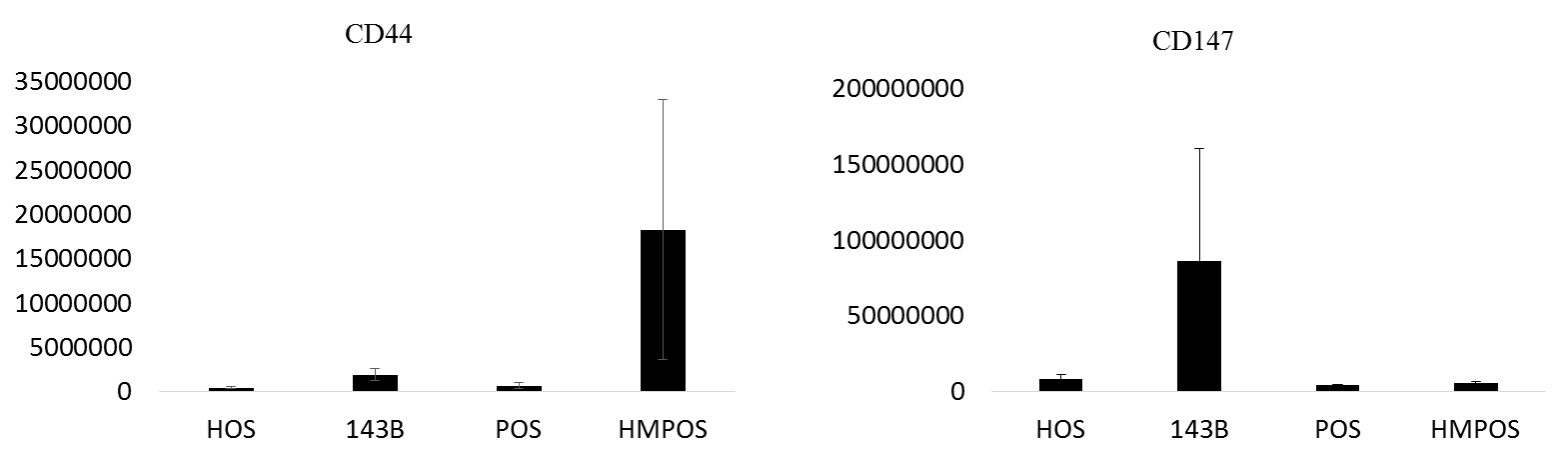


**Fig G:** Image shows quantification of cell pellet immunohistochemistry. Images were quantified by using ImageJ and Adobe Photoshop. The images corresponding are Fig 5C and D. p values are as follows: CD44 human (HOS and 143B) p=0.07, canine (POS and HMPOS) p=0.17. CD147 human (HOS and 143B) p=0.2, canine (POS and HMPOS) p=0.17.


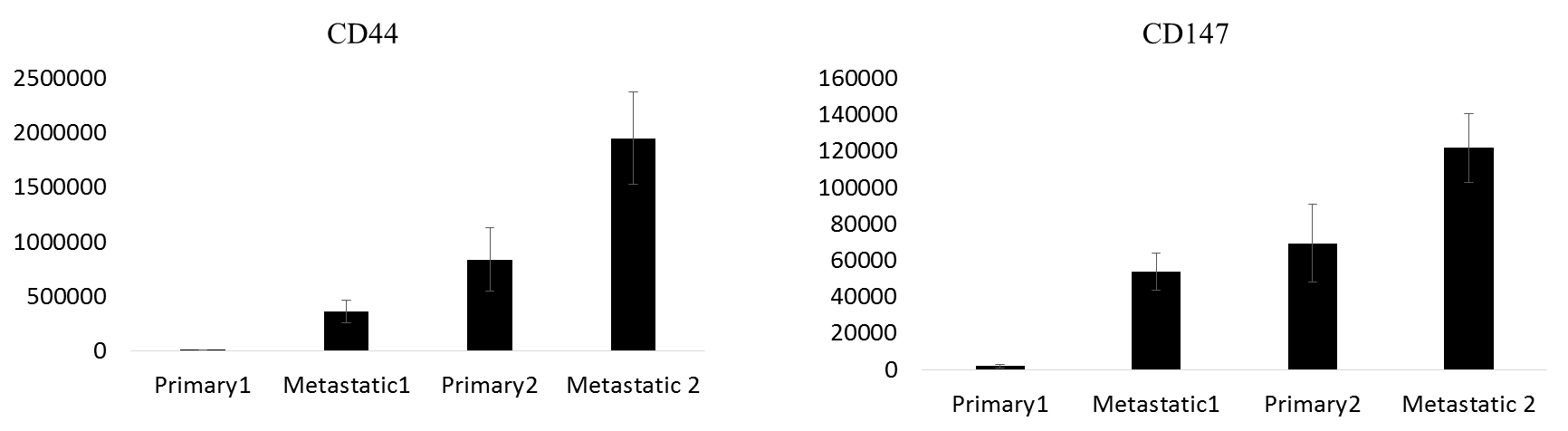


**Fig H:** Image shows quantification of paired primary and metastatic tissue immunohistochemistry. Images were quantified by using ImageJ and Adobe Photoshop. The images corresponding are Fig 6. p values are as follows: CD44 primary and metastatic 1 p=0.01, primary and metastatic 2 p=0.03. CD147 primary and metastatic 1 p=0.003, canine primary and metastatic 2 p=0.01.

**I. Experimental Design and Statistical Rationale:**

- Authors provided a subsection in the Experimental Methods section with the header 'Experimental Design and Statistical Rationale'
- The sample size (n) (for each experiment), as a number (ranges are not acceptable):

1. Sample size n=1. This is since specific targets are validated downstream by 4 separate biochemical techniques with each having n=3 at least.

- The rationale for that (n) choice (statistical power of detection or similar)

1. N=1 is chosen simply to get a list of targets and specific targets of interest will be validated downstream

- Numbers and types of controls employed

1. There are no specific controls since there is only a comparison between 2 cell lines. As an internal control sodium-potassium ATPase has been used which has been mentioned in main text as well as in results table.

- The number of replicates acquired, including a clear distinction between biological (independent data points), process and technical replicates, and rationale for that choice

1. No biological replicates were performed for peptide fingerprinting since specific targets are validated downstream by 4 separate biochemical techniques with each having n=3 at least.

- The criteria for the inclusion or exclusion of data points (if relevant)

1. Only membrane proteins as identified by Uniprot were utilized for characterization and inferences. Contaminations from intracellular proteins was neglected since that was not the focus of the study.

- A description of the statistical methods used for analysis

1. No statistical methods were used for analysis except direct comparison of emPAI values across the results.

- Justification for the statistical methods used for analysis (e.g., has the dataset a normal distribution? etc.)

1. No statistical methods were used for analysis except direct comparison of emPAI values across the results.

**II. Search Parameters and Acceptance Criteria (MS/MS and/or PMF data):**

- Name of peaklist-generating software and release version (number or date)

1. Mascot software

- Name of the search engine and release version (number or date)

1. Mascot software

- Name of sequence database/spectral library searched and release version/date

1. Mascot

- If the database was generated in-house, source of sequences and software used to compile it

N/A

- # of entries in the database (or subset of database) actually searched

1. 8,371

- Specificity of all proteases used to generate peptides

1. Trypsin was used for cleavage.

- # of missed and/or non-specific cleavages permitted

1. 3 missed cleavages permitted

- List of all fixed modification(s) (including residue specificity) considered N/A
- List of all variable modification(s) (including residue specificity) considered N/A
- Mass tolerance for precursor ions

1. 0.3 Da

- Mass tolerance for fragment ions (not required for PMF data)

1. 0.3 Da

- Known contaminants excluded (primarily for PMF data) N/A
- Threshold score/Expectation value for accepting individual spectra

1. P < 0.05

- Justification of the threshold score/expectation value employed

1. A p <0.05 was used since since p<0.05 is used for a 95% confidence rate in most publication standards

- Estimation of false discovery rate (FDR) (for large datasets) and how calculated N/A

**III. Peptide and Protein Identification**

- For all peptide sequences assigned:
- List (in tabular form) of all peptide sequences, including any deviations from expected cleavage specificity

1. Results in uploaded database as mentioned below

- Precursor charge and mass/charge (m/z) for each assignment

1. Results in uploaded database as mentioned below

- All modifications observed

1. Results in uploaded database as mentioned below

- # of matched and unmatched masses (for PMF data) N/A
- Peptide Identification Score(s)

1. Results in uploaded database as mentioned below

- For all protein identifications:
- Accession # (and database from which it is derived)

1. Table TABLE SA- TABLE SD

- # of distinct peptides assigned for each protein

1. Results in uploaded database as mentioned below

- % coverage of each protein assigned (or derived protein identification probability)

1. Results in uploaded database as mentioned below

- Single peptide and PMF identifications, annotated spectra are provided for each protein:
- In a publicly accessible database (and database reference/entry number is provided)

1. Results in uploaded database as mentioned below

**VI. Quantification**

- Quantification measurements for each peptide and protein

# Quantitation: emPAI protocol

The **E**xponentially **M**odified **P**rotein **A**bundance **I**ndex (emPAI) offers approximate, label-free, relative quantitation of the proteins in a mixture based on protein coverage by the peptide matches in a database search result. Developed by Ishihama and colleagues, the key publication is [Ishihama, Y., et al., Exponentially modified protein abundance index (emPAI) for estimation of absolute protein amount in proteomics by the number of sequenced peptides per protein, Molecular & Cellular Proteomics 4 1265-1272 (2005)](http://dx.doi.org/10.1074/mcp.M500061-MCP200)

Unlike the other quantitation protocols, the information required for emPAI is always present in a search result, and there are no parameter settings, so emPAI is "always on", as long as the MS/MS search contains at least 100 spectra.

The formula is very simple: 
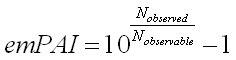


Where *N_observed_* is the number of experimentally observed peptides and *N_observable_* is the calculated number of observable peptides for each protein. The tricky bit is deciding what to include and what to exclude in these two counts.

### The number of observed peptides

The count of observed peptides only includes peptide matches with scores at or above the homology threshold, or the identity threshold, if there is no homology threshold. Ishihama et. al. obtained best proportionality for a standard protein mixture by counting unique parent ions, including different charge states from the same peptide sequence. Mascot 2.4 and earlier followed this same rule, which works well for singly and doubly charged data. However, if peptide matches exist in a number of charge states, such as 2+, 3+, 4+, 5+ and 6+, the rule causes emPAI to be overestimated. Mascot 2.5 and later count unique parent ions only once, regardless of charge state. The difference from the original rule for singly and doubly charged data is negligible compared to the other sources of certainty, as described below.

### The number of observable peptides

To estimate the number of observable peptides, Ishihama et. al. performed explicit *in silico*digests of the protein sequences. The peptide list was then filtered to exclude peptides outside the mass spectrometer scan range and the observed nano-LC retention time range.

For reasons of speed, we prefer to make a calculated estimate of the number of observable peptides based on the protein mass, the average amino acid composition of the database, and the enzyme specificity. The error of doing this is negligible compared with other sources of uncertainty:

- It isn’t practical to filter by retention time, because this information is usually unavailable
- The mass range of the instrument has to be estimated from the range of precursors found in the data set
- Mass range filtering is by Mr, rather than m/z
- The digest is assumed to be a limit digest
- No obvious way to extend the calculation to semi-specific or non-specific digests

In the [supplementary material](http://www.mcponline.org/cgi/content/full/M500061-MCP200/DC1) for Ishihama et. al., there is a worked example for human serum albumin which resulted in a count of 34 for the observable peptides in the Mr range 700 to 2800 and the retention time range 40 to 150 minutes. The enzyme was strict trypsin and no missed cleavages were allowed. The number of peptides estimated by the routine used here is 35.

- Description of how raw mass spectrometric data was processed to yield quantification data
  1. Same as above
- Analytical reliability described (technical replicates and statistical treatments)

1. Sample size n=1. This is since specific targets are validated downstream by 4 separate biochemical techniques with each having n=3 at least.

- Biological reliability described (biological replicates, independent experiments, statistical analyses)

1. The results validated by downstream biochemical techniques show that the results of the peptide fingerprinting are valid.

- Description of any adjustments for systematic errors N/A
- How random error issues were addressed (outliers, exlusion limits etc) N/A
- Estimates of uncertainty for individual proteins N/A
- How the identity of the analyte was verified (in non-database identifications) N/A
- How quantification of multiple isoforms in the same sample was handled N/A
- For spectral counting:
- # of peptides or # of spectra used for the quantification
- Inclusion of modified, semi-tryptic or shared peptides (from different isoforms) N/A

**VII. Raw Data Submission**

- The raw mass spectrometric data has been deposited. (For further information on this requirement, please consult this Editorial.) Yes
- The location and identifying information (url of repository, deposit ID, user name, hash code/identifier, password) are:
- 143B
- <http://msviewer.ucsf.edu/prospector/cgi-bin/mssearch.cgi?report_title=MS-Viewer&search_key=lmo3l6zsmm&search_name=msviewer>
- search term: **lmo3l6zsmm**
- **HOS**
- <http://msviewer.ucsf.edu/prospector/cgi-bin/mssearch.cgi?report_title=MS-Viewer&search_key=wzerq0bzek&search_name=msviewer>
- search term: **wzerq0bzek**
- **POS**
- <http://msviewer.ucsf.edu/prospector/cgi-bin/mssearch.cgi?report_title=MS-Viewer&search_key=muz3bvsexy&search_name=msviewer>
- search term:**muz3bvsexy**
- **HMPOS**
- <http://msviewer.ucsf.edu/prospector/cgi-bin/mssearch.cgi?report_title=MS-Viewer&search_key=jnsbabu0oe&search_name=msviewer>
- search term: **jnsbabu0oe**
